# Psychological impacts of “screen time” and “green time” for children and adolescents: A systematic scoping review

**DOI:** 10.1371/journal.pone.0237725

**Published:** 2020-09-04

**Authors:** Tassia K. Oswald, Alice R. Rumbold, Sophie G. E. Kedzior, Vivienne M. Moore

**Affiliations:** 1 School of Public Health, Faculty of Health & Medical Sciences, The University of Adelaide, Adelaide, South Australia, Australia; 2 Robinson Research Institute, Faculty of Health & Medical Sciences, The University of Adelaide, Adelaide, South Australia, Australia; 3 SAHMRI Women and Kids, South Australian Health and Medical Research Institute, Adelaide, South Australia, Australia; Institute of Physiology and Basic Medicine, RUSSIAN FEDERATION

## Abstract

Technological developments in recent decades have increased young people’s engagement with screen-based technologies (screen time), and a reduction in young people’s contact with nature (green time) has been observed concurrently. This combination of high screen time and low green time may affect mental health and well-being. The aim of this systematic scoping review was to collate evidence assessing associations between screen time, green time, and psychological outcomes (including mental health, cognitive functioning, and academic achievement) for young children (<5 years), schoolchildren (5–11 years), early adolescents (12–14 years), and older adolescents (15–18 years). Original quantitative studies were identified in four databases (PubMed, PsycInfo, Scopus, Embase), resulting in 186 eligible studies. A third of included studies were undertaken in Europe and almost as many in the United States. The majority of studies were cross-sectional (62%). In general, high levels of screen time appeared to be associated with unfavourable psychological outcomes while green time appeared to be associated with favourable psychological outcomes. The ways screen time and green time were conceptualised and measured were highly heterogeneous, limiting the ability to synthesise the literature. The preponderance of cross-sectional studies with broadly similar findings, despite heterogeneous exposure measures, suggested results were not artefacts. However, additional high-quality longitudinal studies and randomised controlled trials are needed to make a compelling case for causal relationships. Different developmental stages appeared to shape which exposures and outcomes were salient. Young people from low socioeconomic backgrounds may be disproportionately affected by high screen time and low green time. Future research should distinguish between passive and interactive screen activities, and incidental versus purposive exposure to nature. Few studies considered screen time and green time together, and possible reciprocal psychological effects. However, there is preliminary evidence that green time could buffer consequences of high screen time, therefore nature may be an under-utilised public health resource for youth psychological well-being in a high-tech era.

## 1 Introduction

### 1.1 Background

The prevalence of mental illness among children and adolescents is increasing globally [1–3]. In particular, depression and anxiety are leading causes of reduced quality of life among children and adolescents [4–8]. Experiences of depressive and anxiety symptoms in childhood and adolescence are associated with an elevated risk of poor mental health in adulthood [8–12], suggesting enduring consequences of compromised mental health while young for well-being and functioning across the lifespan.

Given these lifelong impacts, there is a pressing need to identify and address upstream determinants of mental health, focussing on the prevention of mental illness alongside the promotion of mental well-being. This scoping review focuses on two emerging determinants of interest: time spent engaging with screen-based technologies, referred to as ‘screen time’ (ST), and exposure to or time spent in nature, referred to as ‘green time’ (GT).

With rapid technological developments making access to electronic devices and their presence in our lives pervasive, concern is mounting about the psychological impact of prolonged ST, particularly in children and adolescents [13–18]. A decade ago in a U.S. sample of 8-to-18 year olds, the average ‘total screen time’ was reported at 7.5 hours a day [19] and was highest in 11-to-14 year old adolescents (9 hours). This greatly exceeds recreational ST guidelines of 2 hours or less per day [20]. With widespread integration of digital technologies in school curricula [21], ST is no longer confined to recreational use, making it an inevitable part of young peoples’ lives.

Moderate ST can be beneficial for young people in a connected world [22] as it may afford them with opportunities to enhance existing relationships, forge new connections, engage in safe identity exploration, aid in academic pursuits, and provide access to information about the world beyond their immediate surroundings [23]. However, from a developmental neurobiology perspective, excessive ST may be detrimental to young people as ST stimulates neurobiological systems such as the hypothalamic-pituitary-adrenal (HPA) axis [24] and dopaminergic circuitries [25]. Childhood and adolescence are sensitive periods in which these systems develop and change [26–29], making them particularly vulnerable to insult. From a lifestyle and social perspective, it has been argued that excessive ST also displaces important protective behaviours for mental health such as physical activity [30, 31], getting adequate sleep [32], in-person social interactions [33], and academic activities [34].

As ST has increased, regular engagement with natural environments has concurrently decreased among young people. Children in high-income countries are now experiencing significantly lower levels of contact with nature, or GT, than previous generations [35, 36]. For example, twelve-year-old children in the U.S. report spending an average of less than 6 hours per week outdoors [37], which is less than the average daily screen time for young people. Similarly, in England fewer than a quarter of children reported regularly visiting their local ‘patch of nature’ and less than one in ten children reported regularly playing in wild places, compared to half of all children in the previous generation [38]. Likewise, in a survey of Australian adults, 73% reported playing outdoors more often than indoors when they were children, compared to only 13% of their own children today [39]. Young people’s time spent in nature has been strongly influenced by rapid urbanisation which in many nations has reduced access to both urban greenspaces and private gardens [40].

Benefits of natural environments could be gained through increased physical activity [41, 42] and social connections experienced in greenspaces like parks [41, 43]. Natural areas also tend to be less crowded, with reduced air and noise pollution, which is beneficial for overall health [41]. Furthermore, time spent exposed to natural sunlight helps to regulate circadian rhythms, encouraging healthy sleep-wake cycles and improved sleep [44], which is key for psychological well-being. Several theories within evolutionary and environmental psychology propose that engagement with natural environments is *directly* beneficial for human health and well-being [45, 46]. Notably, Kaplan’s Attention Restoration Theory postulates that nature has specific restorative effects on cognitive functioning [46, 47] and Ulrich’s Stress Reduction Theory contends that nature induces positive affect through reduced stress [45].

Experiences of ST and GT appear to influence psychological well-being in contrasting ways. Screen-based technologies are stimulating and extensive use can potentially displace important protective behaviours, thus they may be detrimental to psychological well-being. Conversely, natural environments may facilitate attention restoration and stress reduction, and support a range of behaviours that promote psychological well-being. As such, it may be argued that the combination of increased ST and decreased GT may be harmful for young people’s mental well-being, and increasing GT may serve as an important ameliorator of ST, to promote mental well-being in an inevitably high-tech era. This may be particularly important for children and adolescents from low socioeconomic backgrounds, who typically engage in greater amounts of ST [48–51] and are also less likely to live in green neighbourhoods [52]. However, research investigating the psychological impacts of ST or GT typically considers ST and GT in isolation and fails to delineate the reciprocal effects of high technology use and low contact with nature on mental health and cognitive outcomes [53]. Such research could give us a greater understanding of 21^st^ century drivers of youth well-being and guide recommendations regarding ST and GT for optimal psychological functioning. We are not aware of any previous review that has attempted to collate evidence about the effects of both of these exposures on child and adolescent psychological outcomes.

This scoping review has four aims:

1. To describe the international literature and evidence regarding the impact of ST and/or GT on psychological outcomes in children and adolescents;
2. To explore the basis for inference about causal links between ST, GT, and psychological outcomes for children and adolescents;
3. To explore the extent to which findings hold, or vary, across the spectrum of socioeconomic status;
4. To investigate the extent to which studies have attempted to delineate the reciprocal effects of ST and GT on psychological outcomes in children and adolescents.

### 1.2 Key definitions

The literature exploring the effects of ST and GT on psychological outcomes in children and adolescents is plagued with inconsistent terminology and calls for clarification and consistency. Therefore, this review will operationalise the following constructs as defined below:

#### 1.2.1 Screen time

*Screen time* (ST) refers to time spent engaging with visual screen-based technologies such as televisions, computers/laptops, videogames, smart phones, tablets/iPads, and handheld electronic or gaming devices. Using the Internet, social media, or communicating via text message are all activities which are included in the definition of ST. Solely auditory activities, such as talking on a phone and listening to music, are not included.

#### 1.2.2 Green time

For the purposes of this review, *green time* (GT) is broadly defined as time spent in, or exposure to, natural environments, elements, or content. This can be further specified as (a) *incidental exposure* to green space and/or natural elements, as measured by residential greenness or level of greenness surrounding schools and commuting environments; (b) *accessibility* to green spaces, public parks, open public spaces, private gardens, or green infrastructure; (c) *purposive use* of green spaces, public parks, private gardens, or green infrastructure; (d) *activities* centred around nature such as wilderness expeditions, gardening, horticultural activities, surfing, or outdoor play; and (e) *educational contexts* such as education outside the classroom or forest schools and kindergartens. Both the *quantity* and *quality* of GT may be considered, which includes attending to the size of green spaces or duration of time spent in green spaces, along with the level of naturalness or specific features of the environments under investigation. A definition of this breadth is necessary given the heterogeneity of existing definitions and lack of consistency when considering GT in the literature.

#### 1.2.3 Psychological outcomes

For the purpose of this review, *psychological outcomes* is a summary term which encapsulates four constructs, measuring a range of psychological variables, including (a) indicators of poor mental health, (b) indicators of positive mental health, (c) cognitive functioning, and (d) academic achievement (Table 1). We have included academic achievement in our scoping review given it can be an indicator of positive psychological functioning [54], integrating aspects of cognitive control such as self-regulation [55], attention [56], executive functions, and working memory [57].

**Table 1:** Psychological outcomes included in the review.

Constructs	Variables	
Indicators of Poor Mental Health	Common internalising or externalising disorders2 or their symptoms, such as:	
	• Depression • Anxiety • Stress • Psychological distress • Poor self-regulation	• Emotional problems • Psychological difficulties • Psychosomatic symptoms • Negative affect or mood
Indicators of Positive Mental Health	Refers to elements of positive psychology or overall well-being, such as:	
	• Happiness • Resilience • Satisfaction with life • Quality of life • Health-related quality of life	• Self-esteem • Optimism • Positive affect or mood • Hope • Prosocial behaviour
Cognitive Functioning	Refers to mental processes, such as:	
	• Attention • Working memory • Executive function	• Visual, spatial, verbal, language, and cognitive development
Academic Achievement	Refers to school measures, such as:	
	• Subject grades • Grade point averages (GPA) • Test or examination results	

## 2 Methods

Due to the diverse and largely observational nature of the existing literature in this field, a *scoping* review was selected as the preferred method. Unlike systematic reviews, scoping reviews have a less focussed research question, attempt to describe the available literature broadly, and include diverse study designs and methods [58]. Further, scoping reviews do not require an evaluation of the quality of the evidence and do not involve a meta-analysis [58]. The current study is referred to as a *systematic scoping review* as a systematic approach has been employed to identify, include, and extract data from studies. This review drew on both the PRISMA Scoping Review Checklist [59] (see S1 File) and Arksey and O’Malley’s widely used framework for scoping reviews [58].

A three-step search strategy was employed. Step one was key word scoping, which involved an initial limited search of relevant databases, followed by an analysis of text words contained in the title, abstract, and index of terms used to describe key articles. Step two involved constructing and performing a systematic search, using the identified keywords and index terms from step one, across selected databases. Step three involved checking reference lists of included publications and manually searching the literature to identify additional relevant studies which may have been missed in the computerised search.

### 2.1 Data source

The following four databases were searched from inception up until 18 February 2019: PubMed, PsycINFO, Embase, and Scopus. The search strategies are available in the S2 File.

### 2.2 Study selection

Results from the systematic search were screened for eligibility according to the inclusion and exclusion criteria outlined below.

### 2.3 Inclusion/exclusion criteria

Studies were included if they met the following criteria:

1. Participants were aged ≤ 18 years, with no serious mental, cognitive, or developmental disorder requiring clinical intervention;
2. The exposure being measured was “screen time” and/or “green time” (as previously defined) and was not used as a part of a mental health intervention in a clinical group. Given the breadth of the literature, studies were only included if they measured the duration of exposure to two or more screen-based activities (e.g., TV and computer use), thus providing a more complete depiction of ST exposure overall for young people. The exception to this was the inclusion of studies measuring only one screen activity if they also measured GT, given the relative rarity of these studies;
3. At least one of the psychological outcomes outlined in Table 1 was reported; and
4. Studies were quantitative, involving analysis of original data, and provided a measure of association between the exposure and outcome of interest.

The search was confined to peer-reviewed English publications, with no publication date limit. Studies only concerned with attention deficit hyperactivity disorder (ADHD), non-common mental health disorders (e.g., schizophrenia, bipolar, or personality disorders) or suicide were excluded. Studies focussing on Internet addiction or other compulsive and problematic technology use were also excluded. Qualitative studies and review papers were excluded.

### 2.4 Data extraction and synthesis

Scoping reviews aim to present an overview of all evidence reviewed. As such, according to Arksey and O’Malley [58], decisions about how to present such a large body of literature need to be made judiciously. Consistent with the intention of the scoping review process, which compels researchers to prioritise certain aspects of the literature as key issues and themes surface [58], a progressively focussed approach was taken in presenting the results.

Data was independently extracted and cross-checked from the included studies by two authors (T.K.O and S.G.E.K) using a form designed and tested by the study authors. In line with Aim 1, descriptive characteristics for all included studies were first charted. We examined the number of publications by research topic and year, the distribution of study samples geographically, the study settings, sample sizes, and the study designs utilised in the included studies. Next, key words were extracted from all included studies to create word clouds illustrating how ST and GT are conceptualised and measured in the literature. Larger text in the word clouds illustrates words used more frequently. Psychological outcomes investigated in the included studies were categorised as either indicators of poor mental health, indicators of positive mental health, cognitive functioning, or academic achievement, as outlined in Table 1.

The key summary statistic (e.g., mean or median and measure of dispersion) for participant age was extracted from each study so it could be assigned to one of four age categories: (a) young children (aged <5 years), (b) schoolchildren (aged 5–11 years), (c) early adolescents (aged 12–14 years), and (d) older adolescents (aged 15–18 years). Cohorts with large age ranges and no details that identified a dominant age group were allocated to a mixed age groups category. The study results were reported by age group to explore potentially different impacts of ST and GT on children and adolescents of specific ages.

To provide an overview of the existing evidence, while respecting the heterogeneity of constructs and measurements, associations between ST, GT, and psychological outcomes reported in the included studies were presented in tables as either unfavourable associations, favourable associations, or not statistically significant. Unfavourable associations were bolded in tables and were representative of increases in the exposure leading to increased poor mental health or decreased positive mental health, cognitive functioning, or academic achievement. Favourable associations were bolded and underscored in tables and were representative of increases in the exposure leading to decreased poor mental health or increased positive mental health, cognitive functioning, or academic achievement. Studies reporting no statistically significant association between exposure and outcome were not bolded in tables. If results were non-linear they were presented narratively in the body of text. Conventional statistical significance was applied, classifying associations with a p-value ≥0.05 as not significant. Study reference numbers and study designs are indicated in the tables (e.g., *114CS =* reference number 114 which was a cross-sectional study).

In addressing Aim 2, which involved exploring the basis for inference about causal links, certain elements of study designs were considered and are discussed. While a formal risk of bias assessment was not performed (not required in scoping reviews [58]), a preliminary assessment of each study’s ability to permit examination of causal linkages, based on two key design features, was conducted. These features included (1) the consideration of baseline psychological profiles in longitudinal studies and (2) the use of comparable groups in experimental or intervention studies. This aided in identifying studies which could address Aim 2. Furthermore, additional variables featured in reported analyses were also extracted from the included studies and discussed. This included, but was not limited to, confounding or mediating demographic and lifestyle variables such as age, sex, physical activity, sleep, and in-person social interactions. Given our focus on socioeconomic status (SES) in Aim 3, indicators of SES were also extracted from each study where available. Studies in which differential associations by SES were investigated and reported were described in text.

In line with Aim 4, a sub-set of studies, which measured both ST and GT were examined to explore the extent to which the psychological effects of ST and GT had been delineated in the existing literature. These studies were relatively rare so each was described briefly in the text as well.

## 3 Results

**Fig 1:**
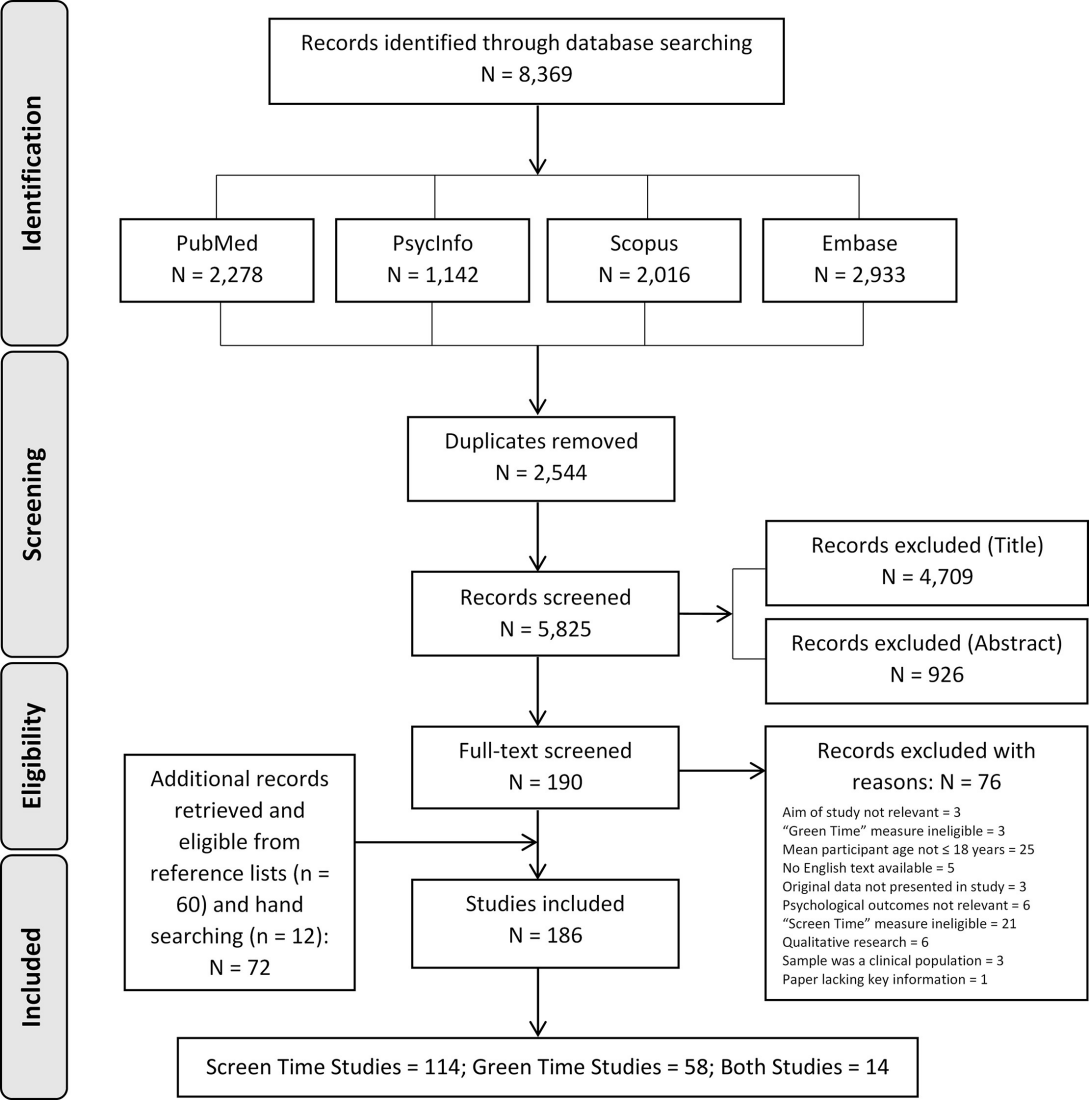
Study search and selection.

The literature search identified 8,369 studies; 8,179 studies were removed because they were either duplicates (n = 2,544) or did not meet the inclusion criteria based on information in the title (n = 4,709) or abstract (n = 926). The full text of 190 studies was assessed for eligibility, of which 114 met the inclusion criteria. Screening the reference lists of the included 114 studies resulted in the identification of a further 60 eligible studies. These studies were not captured in the original search because they did not use key words or index terms related to either (a) psychological outcomes (e.g., study primarily focussed on obesity or body mass index (BMI), physical activity, or sleep), (b) childhood or adolescence (e.g., samples included individuals of all ages), or (c) ST (e.g., where ST was simply a secondary variable in a larger study, or was classified as ‘sedentary behaviour’, which may be different from ST). Twelve additional studies were sourced through manually searching the literature. Consequently, a total of 186 studies were included in the review (see Fig 1).

### 3.1 Description of ST and GT literature

The studies included in the systematic scoping review are displayed in aggregate in Table 2, as counts and percentages, under several descriptive categories. Descriptive characteristics of individual studies can be found in S3 File.

**Table 2:** Collective characteristics of included studies.

		Screen Time (n = 114) n (%)	Green Time (n = 58) n (%)	Both (n = 14) n (%)	All Studies (N = 186) N (%)
**Sample location**^**a**^					
	Asia	8 (7)	1 (2)	0 (0)	9 (5)
	Australia & New Zealand	14 (12)	9 (15.5)	2 (14)	25 (13)
	Canada	12 (10.5)	4 (7)	1 (7)	17 (9)
	Europe	37 (32.5)	20 (34.5)	4 (29)	61 (33)
	Middle East	3 (3)	0 (0)	0 (0)	3 (2)
	South America	3 (3)	0 (0)	0 (0)	3 (2)
	United Kingdom	24 (21)	9 (15.5)	4 (29)	37 (20)
	United States	40 (35)	15 (26)	3 (21)	58 (31)
**Study setting**					
	International	3 (3)	0 (0)	0 (0)	3 (2)
	National	31 (27)	6 (10)	3 (21)	40 (21)
	State	6 (5)	1 (2)	0 (0)	7 (4)
	Region or city	51 (45)	17 (29)	5 (36)	73 (39)
	Other (school/community/services)	23 (20)	34 (59)	6 (43)	63 (34)
**Sample size**^b^					
	Minimum	40	11	76	11
	Median	1,596	214	959	969
	Maximum	388,275	230,929	20,122	388,275
**Study design**^**c**^					
	Cross-sectional	84 (74)	22 (38)	8 (57)	116 (62)
	Cross-sectional (with comparison)	0 (0)	2 (3.5)	0 (0)	2 (1)
	Longitudinal	26 (23)	7 (12)	5 (36)	36 (19)
	Longitudinal (with comparison)	0 (0)	2 (3.5)	0 (0)	2 (1)
	Pre-post design	0 (0)	8 (14)	1 (7)	1 (<1)
	Pre-post design (with comparison)	0 (0)	2 (3.5)	0 (0)	1 (<1)
	Prospective cohort study	6 (5)	1 (2)	0 (0)	7 (4)
	Quasi-experiment	0 (0)	8 (14)	0 (0)	8 (4)
	RCT/Experiment	0 (0)	4 (7)	1 (7)	5 (3)
	Retrospective cohort study	1 (<1)	0 (0)	0 (0)	1 (<1)
	Other	4 (3.5)	2 (3.5)	0 (0)	6 (3)
**Age groups**^**d**^					
	Young children (<5 years)	8 (7)	5 (9)	1 (7)	14 (7)
	Schoolchildren (5–11 years)	18 (16)	22 (38)	4 (29)	44 (24)
	Early adolescents (12–14 years)	39 (34)	11 (19)	1 (7)	51 (27)
	Older adolescents (15–18 years)	13 (11)	4 (7)	3 (21)	20 (11)
	Mixed age groups	36 (32)	17 (29)	5 (36)	58 (31)
**Psychological outcomes**^**e**^					
	Indicators of poor mental health	61 (53.5)	32 (55)	7 (50)	100 (54)
	Indicators of positive mental health	47 (41)	29 (50)	8 (57)	84 (45)
	Cognitive functioning	18 (16)	12 (21)	4 (29)	34 (18)
	Academic achievement	26 (23)	13 (22)	1 (7)	40 (21.5)

Numbers exceed totals, and percentages exceed 100%, in some places because

^a^some studies involved multiple countries

^b^6 GT studies used whole school samples and did not report on final sample number, 1 ST study used whole families in their sample and did not report on final sample number

^c^8 studies involved both cross-sectional and longitudinal analyses

^d^1 GT study stratified results by young children and schoolchildren, and is therefore counted twice

^e^66 studies measured more than one type of psychological outcome.

#### 3.1.1 Research by year and topic

**Fig 2:**
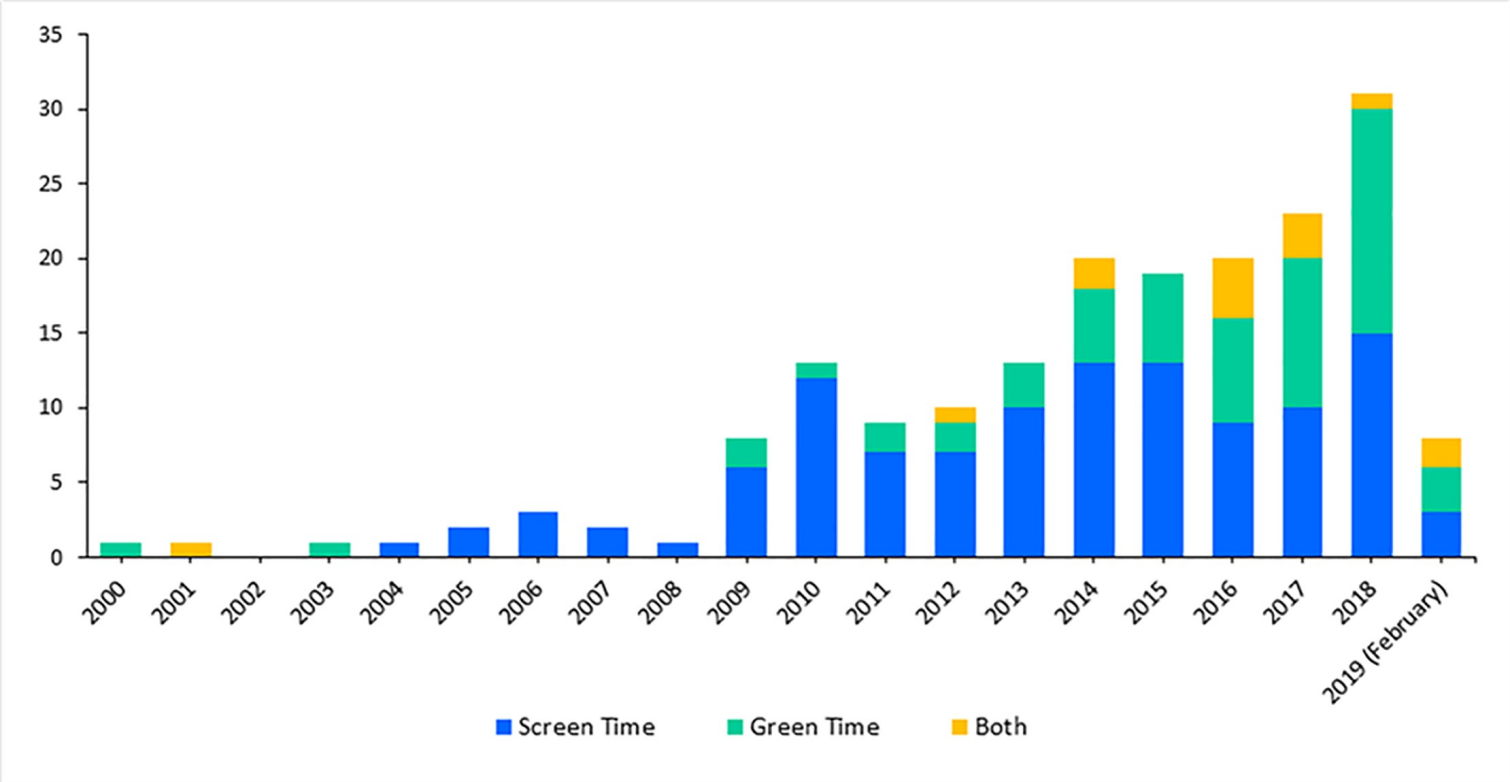
Included studies by research topic and year of publication.

Fig 2 displays the included studies by research topic and publication year (n = 186). The number of studies assessing ST, GT, or both has increased substantially over time. Overall, we identified almost double the number of studies assessing ST (n = 114; 61%) than GT (n = 58; 31%), with only 14 studies (7.5%) assessing both exposures; of these, most were published in the last five years.

#### 3.1.2 Geographic distribution of study samples

Children and adolescents in high-income countries such as the United States (n = 58), the United Kingdom (n = 37), Australia and New Zealand (n = 25), Canada (n = 17), and across Europe (n = 61) were most commonly represented in the literature (Table 2).

#### 3.1.3 Study setting and sample sizes

Sample sizes ranged from 11 to 388,275 participants, with a median of 969. Of the included studies, three (<2%) involved multiple countries, 40 (21%) comprised nationally representative samples, seven (4%) were representative of a state or similar jurisdiction within a country, and the remainder either represented a smaller area such as a region or city, or utilised participants in selected schools, organisations, or with particular characteristics (e.g., obese youth).

#### 3.1.4 Study designs

Cross-sectional studies were by far the most common study design, accounting for 74% of ST studies (n = 85), 42% of GT studies (n = 24), and 57% of the studies that examined both ST and GT (n = 8). Longitudinal studies were relatively more common in the ST literature (n = 26; 23%), while the GT study designs were considerably more variable, due to utilisation of a variety of pretest-posttest (mostly without a control group) and quasi-experimental designs (Table 2).

#### 3.1.5 Age groups

Early adolescents were most commonly studied (n = 51 studies; 27%), followed by schoolchildren (n = 44 studies; 24%), older adolescents (n = 20 studies; 11%), and young children (n = 14 studies; 7%).

#### 3.1.6 Psychological outcomes measured

Over half of the included studies investigated indicators of poor mental health (n = 100; 54%), followed by indicators of positive mental health which were assessed in 45% of studies (n = 84). Indicators of both poor and positive mental health were explored in 25% of studies (n = 46). Fewer studies concerned outcomes related to academic achievement (n = 40; 21.5%) or cognitive functioning (n = 34; 18%). Three studies (<2%) also examined other variables measuring nature connectedness or relatedness, which did not fall into the four categories.

#### 3.1.7 Conceptualisation and measurement of ST and GT in the literature

**Fig 3:**
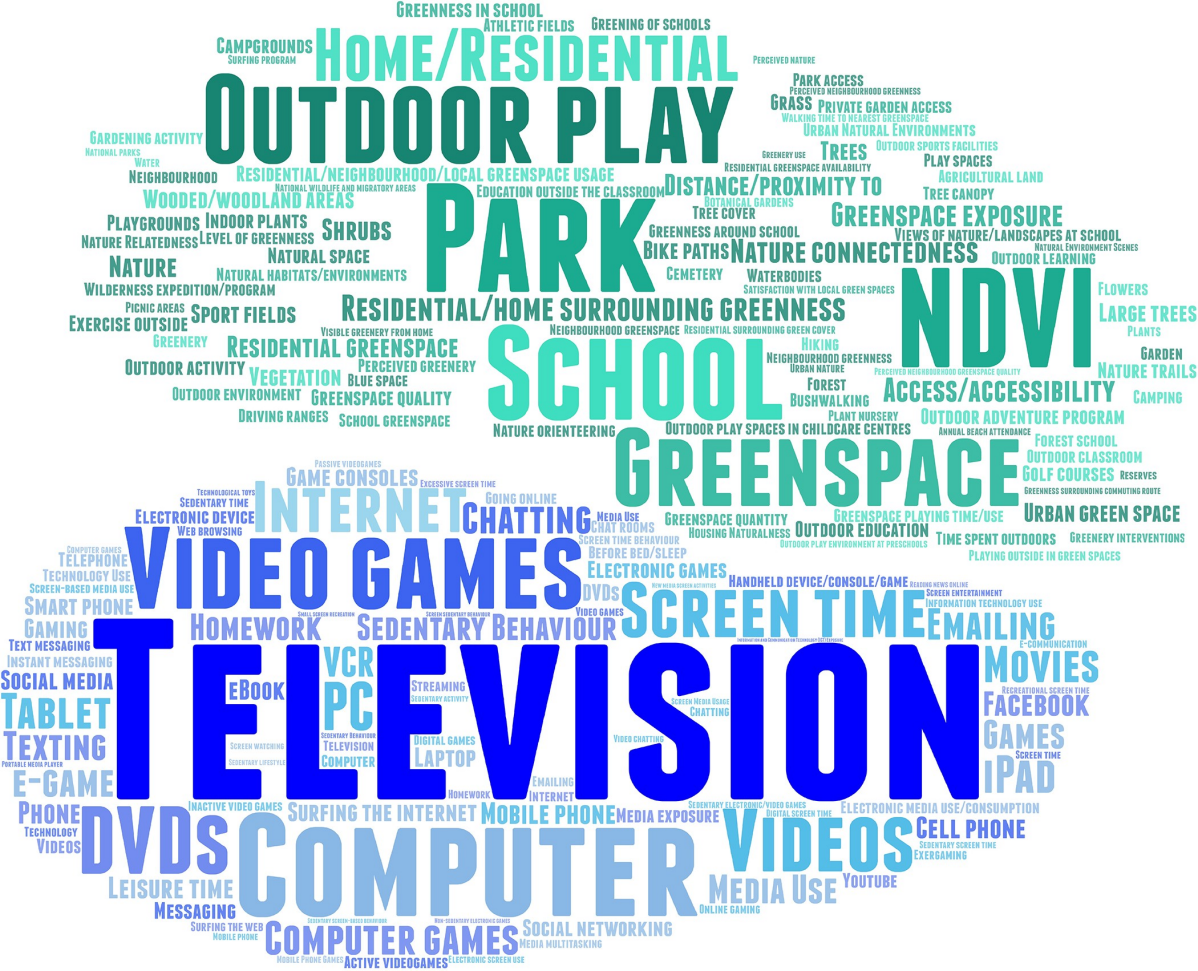
Word cloud of the language used to conceptualise and measure ST and GT in the included studies (ST = 114 studies; GT = 58 studies; Both = 14 studies).

Fig 3 illustrates the language used to conceptualise and measure ST (coloured in blue) and GT (coloured in green) in the included studies. As shown by the larger text, ‘traditional’ screen-based activities such as television watching, videogaming, and computer use are highly represented in the literature. The terminology used for ST is varied, with regular reference to ‘screen time’, ‘sedentary behaviour’, and ‘media use’. Parks, greenspace, and outdoor play were commonly assessed in the GT literature, along with the use of the Normalised Difference Vegetation Index (NDVI) which measures greenness of an area via satellite images [60]. Environmental variables were often measured in and around neighbourhoods, schools, or homes.

### 3.2 The current evidence–associations between ST, GT, and psychological outcomes

An overview of the associations between ST or GT and psychological outcomes in the included studies is presented below. Section 3.2 investigates the overall consistency of findings by age group. This section does not distinguish between study designs, although that information can be found in the tables. Detailed consideration of studies with a longitudinal, experimental, or intervention component, where these permitted examination of causal linkages (e.g., had comparable groups, included baseline psychological profiles, and considered competing explanations or confounding variables), are presented in section 3.3.

#### 3.2.1 Young children (<5 years)

Table 3 presents the results for studies looking at associations between ST (8 studies) or GT (5 studies) and psychological outcomes in young children [61–73]. Studies of young children comprised a total of 30,476 participants in ST studies (plus 483 families with unspecified numbers), 2,836 participants in GT studies, and 575 participants in studies exploring both ST and GT together.

**Table 3:** Results from studies including young children (aged <5 years) (ST = 8 studies; GT = 5 studies).

		Screen Time Exposures							Green Time Exposures						
		1	2	3	4	5	6	7	8	9	10	11	12	13	14
Psychological Outcomes Measured		Hand-held Game Use	ICT Time	Media Exposure	Screen Time	Tablet Use	TV Watching	TV/Video Time	Distance to Greenspaces	Natural Play Environment at Childcare Centres	Outdoor Education / Classroom	Residential Greenness (as measured by the NDVI)	Satisfaction with Greenspaces	Time Spent Outside	Walk in Nature
**Indicators of Poor Mental Health**	Aggression									[62]PP					
	Behaviour Problems				**[65]CS**										
	Conduct Problems (SDQ)									[62]PP					
	Depressed Affect									[62]**PP**					
	Emotional Problems (SDQ)									[62]PP					
	Externalising Problems								[66]CS			[66]CS	[66]CS	[66]CS	
	Hyperactivity/Inattention (SDQ)									[62]PP					
	Internalising Problems								[66]CS			[66]CS^†^; [66]**CS**^**‡**^	[66]CS^†^; [66]**CS**^**‡**^	[66]CS	
	Peer Problems (SDQ)									[62]**PP**					
	Self-Regulation							**[70]RCS**							
	Social-Emotional Delay				[65]CS										
	Total Difficulties (SDQ)				**[72]CS**				[66]CS			[66]CS^†^; [66]**CS**^**‡**^	[66]CS^†^; [66]**CS**^**‡**^	[66]CS	
**Indicators of Positive Mental Health**	Emotional Development										[61]LC*				
	Happiness/Well-being										[64]EC				
	Prosocial Behaviour (SDQ)				**[72]CS**				[66]CS	[62]PP**		[66]CS	[66]CS^†^; [66]**CS**^**‡**^	[66]CS	
	Social Development										[61]LC*				
**Cognitive Functioning**	Attention														[73]EC
	Cognitive Development		**[69]CS**	**[71]L**							[61]LC*				
	Communication Scores						**[63]L**; **[63]CS**								
	Effortful Control	[68]**CS**				**[68]CS**									
	Expressive Language			[67]L											
	Inhibitory Control														[73]EC
	Language Development		**[69]CS**								[61]LC*				
	Language Scores			**[67]L**; **[71]L**											
	Receptive Language			**[67]L**											
	Spatial Working Memory														[73]**EC**
**Academic Achievement**	Not Assessed	Not Assessed													

Study reference number and study design in brackets. Studies reporting an **unfavourable** association between the exposure and outcome are bolded. Studies reporting a **favourable** association between the exposure and outcome are bolded and underscored. Studies reporting no statistically significant association are not bolded.

*Study Designs*: CS = Cross-sectional; PP = Pretest posttest; RCS = Retrospective cohort study; L = Longitudinal; LC = Longitudinal with comparison; EC = Experimental crossover.

*Psychological Outcomes*: SDQ = Strengths & Difficulties Questionnaire.

Where results differ for subgroups

† = association for White British children

‡ = association for South Asian British children

* = significant at measurement time 1 and 2, but not 3 and 4

** = difficult to determine whether effects were due to intervention.

*Other*: ICT = Information & Communications Technology; NDVI = Normalized Difference Vegetation Index.

In this age group, ST exposures were most commonly explored in relation to cognitive functioning and, overall, appeared to show deleterious associations with cognitive development [69, 71], effortful control [68], language [63, 67, 69, 71] and communication [63] abilities. Unfavourable associations between ST and behaviour problems [65], total difficulties [72], self-regulation [70], and prosocial behaviour [72], were also demonstrated across a range of cross-sectional and longitudinal studies. Only two studies in this age group did not report a statistically significant association between a ST exposure and a psychological outcome [65, 67].

The GT research was less consistent, with some studies reporting no statistically significant association between GT exposures and psychological outcomes [62, 64, 66, 73] alongside favourable associations [61, 62, 66, 73]. In one study, incidental GT, such as distance to greenspaces, was not associated with young children’s mental health [66]. However, in this and other studies, green educational contexts [61, 62], satisfaction with greenspaces [66], and residential greenness [66] were favourably associated with a range of psychological outcomes. This included reduced depressed affect, internalising problems, peer problems, and total difficulties, as well as superior prosocial behaviour, cognitive, language, emotional, and social development. Further, one study reported that certain sub-groups, such as young children from ethnic minorities (South Asian British children), benefited from these GT exposures to a greater degree than White British children [66]. Lastly, an experimental study showed that compared to walking in an urban area, going for a walk in nature led to higher spatial working memory for young children [73].

#### 3.2.2 Schoolchildren (5–11 years)

Table 4 presents the results for studies looking at associations between ST (18 studies) or GT (22 studies) and psychological outcomes in schoolchildren [28, 73–111]. Studies of schoolchildren comprised a total of 58,861 participants in ST studies, 252,826 participants in GT studies (plus 1,940 schools with unspecified student numbers), and 15,356 participants in studies exploring both ST and GT together.

**Table 4:** Results from studies including schoolchildren (aged 5–11 years) (ST = 18 studies; GT = 22 studies).

		Screen Time Exposures							Green Time Exposures																				
		1	2	3	4	5	6	7	8	9	10	11	12	13	14	15	16	17	18	19	20	21	22	23	24	25	26	27	28
**Psychological Outcomes Measured**		Computer Use	Electronic Device Use (tablets, phones, games)	Playing Non-Sedentary Videogames	Playing Sedentary Videogames	Total Screen Time / Total Sedentary (Screen) Time / Media Use / Screen Entertainment	Video/Electronic Game Playing	Watching TV/VCR Videos	Access to Greenspace	Agricultural Land around School	Beach Attendance	Education Outside the Classroom / Forest School	Residential/Home, School & Commuting Greenness (as measured by the NDVI)	Greenspaces in the Neighbourhood or School	Greenspace Playing Time	Grass or Shrub Cover	Nature Orienteering Intervention	Outdoor Play Environment	Park/Playground Use	Perceived Restorativeness of School Playground	Private Garden Access	Residential Greenspace Quantity	Residential Naturalness	Residential Proximity to Major Greenspaces	Schoolyard Greening Intervention	Trees / Tree Canopy Cover	Urban Water Cover	Use of Greenspace	Walk in Nature
**Indicators of Poor Mental Health**	Anger											[104]**PPC**																	
	Conduct Problems (SDQ)	[99]CS				**[102]L**; **[80]CS**; [88]CS^B^; **[88]CS**^**G**^	[102]L; **[99]CS**^**B**^; [99]CS^G^	**[102]L**; [99]CS			[74]CS		[74]CS^RS^; [74]CS^SS^; [74]CS^HS^	[85]L^N^	[74]CS				[85]**L**		[85]**L**			[74]CS					
	Cortisol Levels											[28]**LC**																	
	Depression	**[100]CS**					**[100]CS**	[100]CS																					
	Depressive Symptoms	**[83]CS**^**WE**^; [83]CS^WD^	**[83]CS**	[83]CS	**[83]CS**^**WE**^; [83]CS^WD^	**[93]CS**; **[83]CS**^**WE**^; [83]CS^WD^		**[83]CS**^**WE**^; [83]CS^WD^																					
	Emotional Problems (SDQ)	[99]CS				[102]L; [80]CS^B^; **[80]CS**^**G**^; [88]CS^B^; **[88]CS**^**G**^	[102]L; **[99]CS**^**B**^; [99]CS^G^	[102]L; [99]CS			[74]CS	[78]QE	[74]**CS**^**RS**^;[74]CS^SS^; [74]CS^HS^	[85]L^N^	[74]**CS**				[85]L		[85]L			[74]CS					
	Hyperactivity/Impulsivity										[74]CS		[74]CS^RS^; [74]CS^SS^; [74]CS^HS^		[74]CS			[95]CS^SV^; [95]CS^Q^						[74]CS					
	Hyperactivity/Inattention (SDQ)	[99]CS				[80]CS; [88]CS; [102]L	[102]L; [99]CS^G^; **[99]CS**^**B**^	[102]L; **[99]CS**^**B**^; [99]CS^G^	[111]**CS**		[74]CS	[78]QE	[74]**CS**^**RS**^; [74]**CS**^**HS**^; [74]CS^SS^	[85]L^N^	[74]CS				[85]**L**		[85]**L**			[74]CS					
	Mental Health											[89]**QE**^**B**^; [89]QE^G^																	
	Negative Affect	**[100]CS**																		[75]PP									
	Negative Thinking						**[100]CS**	[100]CS																					
	Peer Problems (SDQ)	[99]CS				[80]CS; [88]CS; [102]L	[102]L; [99]CS	[102]L; [99]CS			[74]**CS**	[78]QE	[74]CS^RS^; [74]CS^SS^; [74]CS^HS^	[85]L^N^	[74]**CS**				[85]**L**		[85]**L**			[74]CS					
	Perceived Stress					[80]CS																							
	Psychological Distress																						[109]**CS**						
	Short-tempered	**[100]CS**					**[100]CS**	**[100]CS**																					
	Sleeplessness	**[100]CS**					**[100]CS**	**[100]CS**																					
	Somatic Complaints	**[100]CS**					**[100]CS**	[100]CS																					
	Stress											[104]PPC																	
	Total Difficulties (SDQ)	**[101]CS**				[88]CS; **[90]CS**		**[101]CS**	[111]**CS**		[74]**CS**		[74]**CS**^**RS**^; [74]**CS**^**HS**^; [74]CS^SS^		[74]**CS**									[74]CS					
**Indicators of Positive Mental Health**	Emotional Functioning (HRQoL)																					[96]CS			[108]QE			[96]CS	
	Energy											[104]PPC^*^																	
	Global Self-Worth																						[109]**CS**						
	Happiness	**[84]CS**				**[84]CS**	**[84]CS**	**[84]CS**				[104]**PPC**																	
	Health-Related Quality of Life					[103]CS																[96]CS						[96]**CS**	
	Positive Affect																			[75]**PP**									
	Prosocial Behaviour (SDQ)					[80]CS; [88]CS; [102]L	[102]L	[102]L			[74]**CS**	[78]**QE**	[74]CS^RS^; [74]CS^SS^; [74]CS^HS^		[74]CS									[74]CS					
	School Functioning (HRQoL)																					[96]CS						[96]CS	
	Self-Esteem					[91]CS											[76]PP					[96]CS						[96]**CS**	
	Self-Rated Health					[80]CS																							
	Social Perception	[105]**CS**						**[105]CS**																					
**Cognitive Functioning**	Attention	[105]CS				**[87]PS**^**†**^; **[106]CS**; **[106]L**	**[106]CS**; **[106]L**	**[105]CS**; **[106]CS**; [106]L			[74]CS		[74]**CS**^**RS**^; [74]CS^SS^; [74]CS^HS^; [82]L^RS^; [82]L^C^; [82]**L**^**S**^; [82]**L**^**T**^		[74]CS			[95]CS^SV^; [95]**CS**^**Q**^						[74]CS					[73]**EC**
	Attention Restoration																								[108]**QE**				
	Executive Functioning	[105]CS						**[105]CS**																					
	Inhibitory Control																												[73]EC
	Language, Memory & Learning	[105]**CS**						**[105]CS**																					
	Spatial Working Memory																												[73]EC
	Superior Working Memory												[82]L^RS^; [82]L^C^; [82]**L**^**S**^; [82]**L**^**T**^																
	Visuospatial Processing	[105]CS						**[105]CS**																					
	Working Memory												[82]L^RS^; [82]**L**^**C**^; [82]**L**^**S**^; [82]**L**^**T**^																[73]EC
**Academic Achievement**	Language / Arts Scores	[79]**CS**^**PR**^; [79]CS^SR^; **[98]L**					[79]CS	TV: [79]CS^PR^; [79]**CS**^**SR**^VCR: [79]CS^PR^; **[79]CS**^**SR**^; [98]L					[110]**CS**^**SS**^																
	Mathematics Scores	[79]**CS**^**PR**^; [79]CS^SR^; [98]L					[79]CS	TV: [79]CS^PR^; [79]**CS**^**SR**^ VCR: [79]CS^PR^; **[79]CS**^**SR**^; [98]L					[110]**CS**^**SS**^	**[77]CS**^**S**^		[92]CS^U^; [94]CS^U^; [94]CS^S^										[92]CS^U^; [94]**CS**^**S**^; [94]CS^N^	[92]CS		
	Reading Scores	[79]**CS**^**PR**^; **[79]CS**^**SR**^					[79]CS	TV: [79]CS^PR^; [79]**CS**^**SR**^ VCR: [79]CS^PR^; **[79]CS**^**SR**^						**[77]CS**^**S**^		[92]CS^U^; [94]CS^U^; [94]CS^S^										[92]**CS**^**U**^; [94]CS^S^; [94]CS^N^	[92]CS		
	School Grades/Performance					**[86]PS**; **[98]CS**																							
	Science Grades	[98]L						[98]L																					
	School Test Scores									[107]CS			[107]CS^SS^			[107]CS^SS,RUS^; [107]**CS**^**SS,US**^										[107]CS^SS,RUS^; [107]**CS**^**SS,US**^			
	Writing Scores													**[77]CS**^**S**^															

Study reference number and study design in brackets. Studies reporting an **unfavourable** association between the exposure and outcome are bolded. Studies reporting a **favourable** association between the exposure and outcome are bolded and underscored. Studies reporting no statistically significant association are not bolded.

*Study Designs*: CS = Cross-sectional; CSC = cross-sectional with comparison; EC = experimental crossover; LC = longitudinal study with comparison; L = Longitudinal; PP = pre-post-test design; PPC = pre-post-test design with comparison; PS = prospective study; QE = quasi-experimental.

*Where results differed for subgroups*: B = result for boys; G = result for girls; WE = ST on weekend days; WD = ST on weekdays; PR = when ST is parent-reported; SR = when ST is self-reported by child; RUS = rural schools; US = urban schools

* = results were strongest for students with poor behaviour.

*Green Time Exposure Details*: RS = Residential surrounding; SS = Surrounding school; HS = Home-school; C = Commuting; S = School; T = Total surrounding (home, school & commuting); SV = Sky view; Q = Quality; U = Urban; N = Neighbourhood; NDVI = Normalized Difference Vegetation Index.

*Psychological Outcomes*: SDQ = Strengths & Difficulties Questionnaire; HRQoL = Health-related Quality of Life subscale

† = association mediated by reduced sleep.

*Study Notes*: Studies [81]CS and [97]L were not included in this table due to unclear reporting of results.

Overall, study results were inconsistent, with 11 ST studies [79, 80, 83, 88, 91, 99, 100, 102, 103, 105, 106] and 15 GT studies [73–76, 78, 82, 85, 89, 92, 94–96, 104, 107, 108] reporting no statistically significant association between at least one exposure and psychological outcome variable measured. However, where statistically significant associations were reported, ST exposures were generally associated with unfavourable psychological outcomes (*n* = 16 studies), while GT exposures were typically associated with favourable psychological outcomes (*n* = 18 studies).

The majority of studies explored the impacts of total ST and it was not clear whether a particular type of screen activity was most influential for schoolchildren in the available literature. ST was most commonly associated with unfavourable outcomes on measures of poor mental health, such as depression/depressive symptoms [83, 93, 100], conduct problems [80, 88, 99, 102], emotional problems [80, 88, 99], negative affect [100], total difficulties [90, 101], and being short-tempered, experiencing sleeplessness, and voicing somatic complaints [100]. In some studies, stratifying ST by weekend and weekday use, child- and parent-report, or by gender, revealed differential psychological associations. In general, weekend [83] and self-reported ST [79] were associated with a wider range of adverse psychological outcomes, however this varied significantly by gender. ST was also associated with various measures of cognitive functioning, including poorer attention [87, 105, 106], and executive functioning, language, memory, learning and visuospatial processing [105] for schoolchildren. Further, higher ST was associated with reduced happiness [84] and poorer academic outcomes [79, 86, 98] in some studies.

A wide range of GT exposures were considered for schoolchildren. Education outside the classroom and forest schools (n = 4 studies) were reported as largely beneficial, being associated with reduced anger [104], healthier cortisol profiles (indicative of reduced stress) [28], increased energy [104], happiness [104], and prosocial behaviour [78], along with improved overall mental health for boys [89]. In one study, schoolchildren who perceived their schoolyard as more restorative experienced greater positive affect following recess time [75]. A schoolyard greening intervention resulted in increased attention restoration [108], while an experiment demonstrated that a brief walk in nature was associated with increased attention [73].

In some studies, higher surrounding greenness in a child’s environment, as measured by the NDVI, was associated with better mental health (lower emotional problems, hyperactivity/inattention problems, and total difficulties [74]), greater cognitive functioning (improved attention [74, 82], superior working memory [82], and working memory [82]), and better language/arts and math performance at school [110]. However, these associations differed by residential and school surrounding greenness. Furthermore, one study highlighted that greener environments appeared to benefit children academically in urban schools but not rural schools [107]. One study found that higher greenness was associated with poorer school performance [77]; however, this was proposed to be reflective of greenspace being associated with lower SES communities in New Zealand.

Residential proximity to major greenspaces was not associated with any psychological outcomes in one cross-sectional study [74], whereas having access to a private garden and park use were associated with lower conduct, hyperactivity/inattention, and peer problems, in a longitudinal study [85]. In the same cross-sectional study [74], greenspace playing time was associated with lower emotional problems, peer problems, and total difficulties.

#### 3.2.3 Early adolescents (12–14 years)

Table 5 presents the results for studies looking at associations between ST (39 studies) or GT (11 studies) and psychological outcomes in early adolescents [112–161]. Studies of early adolescents comprised a total of 97,820 participants in ST studies, 4,100 participants in GT studies, and 20,122 participants in studies exploring both ST and GT together.

**Table 5:** Results from studies including early adolescents (aged 12–14 years) (ST = 39 studies; GT = 11 studies).

		Screen Time Exposures												Green Time Exposures								
		1	2	3	4	5	6	7	8	9	10	11	12	13	14	15	16	17	18	19	20	21
Psychological Outcomes Measured		Computer Use	Gaming (video and computer)	Internet Use	Media Multitasking	Playing Video Games	Recreational Screen Time	Screen Time for Homework	Screen Time/Media Use/ Screen-Based Media Time	Small Screen Recreation	Social Media Use	Tablet/ Mobile/Cell Phone Use	Watching TV/ Videos/ DVDs	Greening Schoolyards	Greenspace	Hiking Camp	Indoor Plants	Outdoor Education Program	Outdoor Learning	Percentage Parkland in Neighbourhood	Surfing Program Participation	Viewing Natural Environmental Scenes
**Indicators of Poor Mental Health**	Anxiety										[127]CS						[132]QE					
	Anxiety Symptoms	[142]CS				**[142]CS**			**[118]CS**; **[142]CS**				[142]CS									
	Conduct Problems (SDQ)			[117]CS		**[117]CS**							[117]CS									
	Demand (PSQ)															[147]**PP**						
	Depressed Affect								[157]CS^B^; **[157]CS**^**G**^													
	Depression	[130]CS^B^; **[130]CS**^**G**^							**[116]CS**; [130]CS				[130]CS									
	Depressive Symptoms	**[142]CS**				**[142]CS**			**[118]CS**; **[134]CS**; **[142]CS**		[127]CS		[142]CS		[131]L^QL^; [131]L^QN^							
	Emotional Problems (SDQ)			[117]CS		[117]CS							[117]CS									
	Externalising Problems								**[152]CS**						[125]**CS**^**QL,CR**^;[125]**CS**^**QL,PR**^; [125]CS^QL,TR^; [125]CS^QN^							
	Health Complaints								**[135]CS**													
	Hyperactivity/ Inattention (SDQ)			**[117]CS**		[117]**CS**							[117]CS									
	Internalising Problems			[119]CS		[119]CS^CR^			**[152]CS**						[125]**CS**^**QL,CR**^; [125]**CS**^**QL,PR**^; [125]CS^QL,TR^; [125]CS^QN,CR^; [125]**CS**^**QN,PR**^; [125]CS^QN,TR^;							
	Major Depressive Disorder			[150]CS		[150]CS							[150]CS									
	Mental Health Diagnosis			[119]CS		[119]CS																
	Mental Health Problems								**[152]CS**													
	Mood									[153]ES												
	Negative Affect																				[133]PP	
	Peer Problems (SDQ)			[117]CS		[117]CS							[117]CS									
	Perceived Stress																			[124]**CS**		
	Physiological Stress													[139]**QE**								
	Total Difficulties (SDQ)	[113]L		[117]CS; [119]CS^PR^		[117]CS; [119]CS^PR,B^; **[119]CS**^**PR,G**^	[113]L; [140]CS	[113]L				[113]L	[117]CS; **[113]L**		[125]**CS**^**QL,CR**^;[125]**CS**^**QL,PR**^; [125]CS^QL,TR^; [125]CS^QN,CR^; [125]**CS**^**QN,PR**^; [125]CS^QN,TR^			[145]PPC				
	Worries (PSQ)															[147]PP						
**Indicators of Positive Mental Health**	Connectedness Towards School																				[133]**PP**	
	Emotional Functioning (HRQoL)		**[120]CS**						[128]CS^B^; **[128]CS**^**G**^													
	Extrinsic Motivation																		[123]QE			
	General Health (HRQoL)		**[120]CS**																			
	Happiness														[160]**OS**^**E**^	[147]PP						
	Health Status								**[135]CS**													
	Health-related Quality of Life		**[120]CS**; **[141]CS**						**[141]CS**; **[146]CS**^**BS**^			**[146]CS**^**BS**^	**[141]CS**; [146]CS^BS^									
	Intra-Psychic Balance (Well-being)													[139]**QE**								
	Intrinsic Motivation																		[123]QE			
	Mindfulness															[147]**PP**						
	Mood																					[161]RCT
	Positive Affect																				[133]PP	
	Prosocial Behaviour (SDQ)			[117]CS		[117]CS							[117]CS					[145]PPC^BG^; [145]**PPC**^**WG**^				
	Psychological Well-being	**[113]L**					**[113]L**	[113]L				[113]L	[113]L									
	Psychosocial Functioning (HRQoL)		**[120]CS**																			
	Quality of Life								**[135]CS**													
	Satisfaction with Appearance																				[133]**PP**	
	Satisfaction with Life								[144]CS		**[127]CS**; [127]L		[127]CS; [127]L		[160]**OS**^**E**^	[147]**PP**		[145]PPC^BG^; [145]**PPC**^**WG**^			[133]PP	
	School Functioning (HRQoL)		**[120]CS**						**[128]CS**													
	School Life Satisfaction								**[118]CS**													
	Self-Concept																		[123]QE			
	Self-efficacy			[117]CS^B^; **[117]CS**^**G**^		[117]CS							[117]CS			[147]PP						
	Self-esteem	**[148]CS**		[136]CS; [137]CS		**[136]CS**; [137]CS			[128]CS^B^; **[128]CS**^**G**^; **[148]CS**; [157]**CS**^**B**^; **[157]CS**^**G**^			[136]CS; [137]CS	**[148]CS**^**DVD**^ [148]CS^TV^					[145]**PPC**^**BG**^; [145]**PPC**^**WG**^				[161]RCT
	Self-perceived Health	[143]CS^WE^; **[143]CS**^**WD,B**^; [143]CS^WD,G^											[143]CS									
	Self-rated Health								[144]CS													
	Well-being	[143]CS											**[143]CS**^**WE,B**^; [143]CS^WE,G^; [143]CS^WD^	[139]QE	[160]**OS**^**E**^		[132]QE					
**Cognitive Functioning**	Attention				**[115]L**	[126]CS			[159]CS				[126]CS									
	Attention Shifting	**[159]CS**	[159]CS										[159]CS									
	Cognitive Development														[160]OS^E^							
	Creative Thinking & Problem Solving																	[145]PPC^BG^; [145]**PPC**^**WG**^				
	Executive Function								[159]CS					[139]QE								
	Flexibility of Attention	**[159]CS**	[159]CS										[159]CS									
	Inhibition				[114]CS^EFT^; **[114]CS**^**CR**^																	
	Shifting				[114]CS^DTT^; **[114]CS**^**CR**^																	
	Visual Memory								[159]CS													
	Visual-Spatial Abilities			[137]CS; [138]L		[137]**CS**; [138]**L**						[137]CS										
	Visuospatial Working Memory	[159]CS	**[159]CS**										[159]CS									
	Working Memory				[114]CS^DST^; **[114]CS**^**CR**^																	
**Academic Achievement**	Academic Achievement		[121]PC	**[121]PC**		[155]CS			**[112]CS**; **[121]PC**; **[129]CS**; **[154]L⁺**				**[121]PC**; [155]CS^WE^; **[155]CS**^**WD**^				[132]QE					
	Arithmetic Skills																		[123]QE^±^			
	GPA		[122]CS	**[122]CS**; [137]CS; [138]L		[126]CS; **[137]CS**; **[138]L**		[122]CS*	**[158]CS**			[137]CS	[122]CS; [126]CS									
	Language Achievement/ Scores	[149]L	[122]CS; [149]L	**[122]CS**; [149]L				[122]CS*	**[149]CS**; [149]L			[149]L	[122]CS; [149]L									
	Math Achievement/ Ability/ Scores	**[149]L**	[122]CS; [149]L	[119]CS; **[122]CS**; [137]CS; [138]L; **[149]L**		[119]CS; [137]CS; [138]L		[122]CS*	**[149]CS**; **[149]L**			[137]CS; [149]L	[122]CS; [149]L									
	Reading Ability			[137]CS; [138]**L**; [119]CS^B^; **[119]CS**^**G**^		[137]CS; [138]L; [119]CS^B^; **[119]CS**^**G**^						[137]CS										
	School Grades			[137]CS; [138]**L**		[137]CS; **[138]L**						[137]CS										
**Other**	Nature Connectedness																				[133]PP	

Study reference number and study design in brackets. Studies reporting an **unfavourable** association between the exposure and outcome are bolded. Studies reporting a **favourable** association between the exposure and outcome are bolded and underscored. Studies reporting no statistically significant association are not bolded.

*Study Designs*: CS = cross-sectional; ES = ecological momentary assessment study; L = longitudinal; OS = observational study; PC = prospective cohort; PP = pre-post test design; PPC = pre-post with comparison; QE = quasi-experimental; RCT = randomised controlled trial.

*When results differ for subgroups*: B = results for boys; G = results for girls.

*Green Time Exposure details*: QL = quality; QN = quantity; E = exposure; ± = both the outdoor group and traditional classroom group improved significantly over time, but it is not clear whether these improvements differed by group.

*Screen Time Exposure details*: BS = before sleep; WE = weekend screen time; WD = weekday screen time; DVD = for DVD viewing only; TV = for TV viewing only

* = studying with a computer was not significant, but studying without a computer was favourable; ⁺ = association was mediated by an increase in sensation seeking.

*Psychological Outcomes*: CR = child-reported; PR = parent-reported; TR = teacher-reported; BG = between-group difference; WG = within-group difference; EFT = measured with the Eriksen Flankers Task; DTT = measured with the Dots-Triangle Task; DST = measured with the Digit Span Task; SDQ = Strengths & Difficulties Questionnaire; HRQoL = Health-related Quality of Life subscale; PSQ = Perceived Stress Questionnaire.

*Study Notes*: Study [151]CS was not included in the table due to unclear reporting of results; Study [156]CS is described in text due to comparison of cluster types.

Twenty-four ST studies [113, 114, 117, 119, 121, 122, 126–128, 130, 136–138, 140, 142–144, 148–150, 153, 155, 157, 159] and 10 GT studies [123, 125, 131–133, 139, 145, 147, 160, 161] reported at least one association with a psychological outcome that was not statistically significant. However, where statistically significant associations were reported, ST exposures were generally associated with unfavourable psychological outcomes (*n* = 32 studies), while GT exposures were typically associated with favourable psychological outcomes (*n* = 8 studies).

TV watching time was largely unrelated to all psychological outcomes in this age group. Studies measuring total ST were most common and generally reported unfavourable associations with a range of psychological outcomes. Total ST was associated with indicators of poor mental health such as higher anxiety symptoms [118, 142], depression/depressive symptoms [116, 118, 134, 142], depressed affect (in girls) [157], externalising problems [152], internalising problems [152], health complaints [135], and overall mental health problems [152].

Total ST was also associated with reduced positive mental health such as lower health status [135], health-related quality of life [141, 146], quality of life [135], psychological well-being [113], school functioning [128], school life satisfaction [118], and lower emotional functioning [128] and self-esteem [148] (particularly for girls [128, 157]). Two studies reported an important distinction between *screen* sedentary behaviour and *non-screen* sedentary behaviour (e.g., reading), whereby screen sedentary behaviour was associated with lower self-esteem, but non-screen sedentary behaviour was not [148, 157]. Furthermore, a study which compared ‘clusters’ of different types of technology users [156] found that early adolescents who were labelled as ‘instrumental computer users’ (characterised as high email and general computer users) had more favourable self-efficacy and mood scores when compared to ‘multi-modal e-gamers’ and ‘computer e-gamers’, although some gender differences were present.

Overall, higher total ST was associated with lower academic achievement [112, 121, 129, 154], GPA [158], language achievement [149], and math achievement [149]. While associations between ST and measures of cognitive functioning were less clear, playing video games was associated with better visual-spatial abilities in two studies, cross-sectionally [137] and longitudinally [138], for early adolescents. In other studies, computer use was associated with poorer attention measures cross-sectionally [159], while media multitasking was associated with poorer attention longitudinally [115]. In the same longitudinal study, no reversed effects from attention problems, on media multitasking over time, were found [115]. In a cross-sectional study, media multitasking was not associated with inhibition, attention shifting, or working memory when measured by objective cognitive tests; however, when early adolescents reported their daily difficulties in these subcomponents of executive function, it was found that media multitasking was unfavourably associated with these self-reports [114].

Inconsistent findings for the GT exposures were found in this age group. Outdoor education programs and hiking camps were associated with increased satisfaction with life [147], mindfulness [147], and self-esteem [145]. A schoolyard greening intervention was associated with decreased physiological stress and increased well-being [139], whereas introducing plants into classrooms did not alter early adolescents’ anxiety, well-being, or academic achievement in another study [132]. Outdoor learning was associated with greater improvement of math skills in a quasi-experiment [123], but these results should be interpreted with caution due to significant baseline differences between groups.

A higher percentage of parkland in neighbourhoods was associated with lower perceived stress for early adolescents in a cross-sectional geographic study [124], but in an RCT, viewing scenes of natural environments on a screen was not associated with changes in mood or self-esteem [161]. Findings pertaining to early adolescents’ greenspace exposure and psychological outcomes were inconsistent, with results varying according to greenspace quality and quantity, and whether psychological variables were self-, parent- or teacher-reported [125, 131, 160]. Overall, few studies looking at the effects of GT on cognitive functioning and academic achievement were identified for this age group.

#### 3.2.4 Older adolescents (15–18 years)

Table 6 presents the results for studies looking at associations between ST (13 studies) or GT (4 studies) and psychological outcomes in older adolescents [22, 42, 162–176]. Studies of older adolescents comprised a total of 155,418 participants in the ST studies, 1,053 participants in the GT studies, and 2,065 participants in studies exploring both ST and GT together.

**Table 6:** Results from studies including older adolescents (aged 15–18 years) (ST = 13 studies; GT = 4 studies).

			Screen Time Exposures											Green Time Exposures	
		1	2	3	4	5	6	7	8	9	10	11	12	13	14
Psychological Outcomes Measured		Accessing e-news or study materials online	Computer Use	Electronic Media Use Before Bed / Before Sleep	Gaming (Video, Computer, Internet, e-games)	Being Online in Bed (Facebook, Chat, etc)	Internet Time	Playing Video Games	Social Media Sites/Apps	Telephone/Smartphone Use/Texting	Total Screen Time / Media Use	Watching TV	Watching Videos	Outdoor Program / Camp Experience	Wilderness Expedition
**Indicators of Poor Mental Health**	Aggression													[173]PP	
	Anxiety	**[176]CS**^**s**^			[170]CS^*^; [176]CS				**[176]CS**			**[170]CS**^**M**^; [170]CS^F^	[176]CS	[175]QE; [173]**PP**	
	Anxiety Symptoms										**[165]CS**				
	Conduct Problems				**[170]CS**							[170]CS			
	Depression				**[170]CS**							**[170]CS**		[175]QE; [173]PP	
	Depressive Symptoms		**[162]CS**; **[168]CS**	**[169]CS**		**[169]CS**		**[169]CS**^**IB**^; **[162]CS**		[169]CS^IB^	**[162]CS**; **[165]CS**; **[174]CS**; [164]CS^M^; **[164]CS**^**F**^	[162]CS; [168]CS; [169]CS^IB^			
	General Emotional, Behavioural & Social Problems				**[170]CS**							[170]CS^M^; **[170]CS**^**F**^			
	Oppositional Defiant Problems				**[170]CS**							[170]CS^M^; **[170]CS**^**F**^			
	Psychological Distress		**[171]CS**					**[171]CS**		[171]CS	[171]CS; **[174]CS**	[171]CS			
	Somatic Symptoms/Complaints										[165]CS				
	Total Difficulties (SDQ)		[171]CS^⁺^					[171]CS		[171]CS	**[171]CS**	[171]CS		**[175]QE**^**ST**^; [175]**QE**^**LT**^	
**Indicators of Positive Mental Health**	Emotional Functioning (HRQoL)		**[163]CS**				[166]CS	**[163]CS**; [166]CS			**[163]CS**; [166]CS	[163]CS; [166]CS			
	Global Health		[171]CS					**[171]CS**		[171]CS	[171]CS	[171]CS			
	Health-related Quality of Life		**[163]CS**; [171]CS				[166]CS	**[163]CS**; **[171]CS**; [166]CS		[171]CS	**[163]CS**; **[171]CS**; [166]CS	[163]CS; [171]**CS**; [166]CS			
	Mental Well-being									**[22]CS**					
	Positive Identity														[172]**PP**
	Psychological Strengths													[175]QE	
	Psychosocial Score (HRQoL)		**[163]CS**					**[163]CS**			**[163]CS**	[163]CS			
	Quality of Life		[171]CS					**[171]CS**		[171]CS	**[171]CS**	[171]CS			
	Satisfaction with Life	[176]CS			[176]CS				[176]CS		**[165]CS**	**[176]CS**^**S**^	[176]CS		
	School Functioning (HRQoL)		[163]CS				**[166]CS**^**SP,M**^;[166]CS^SP,F^; [166]CS^VP^	**[163]CS**; **[166]CS**^**SP,M**^; [166]CS^SP,F^; [166]CS^VP^			**[163]CS**; **[166]CS**^**SP,M**^; [166]CS^SP,F^; [166]CS^VP^	[163]CS; [166]CS			
	Self-Efficacy													[173]**PP**	
	Self-esteem	[176]CS			[176]CS				[176]CS		**[165]CS**; **[174]CS**	**[176]CS**^**S**^	[176]CS		
	Social Functioning (HRQoL)		**[163]CS**				[166]CS	[163]CS; [166]CS			**[163]CS**; [166]CS	[163]CS; [166]CS^SP^; **[166]CS**^**VP,M**^; [166]CS^VP,F^			
	Well-being													[173]PP	
**Cognitive Functioning**	Attention				**[170]CS**							[170]CS			
**Academic Achievement**	Academic Achievement	[176]CS	**[167]CS**		[176]CS			**[167]CS**	**[176]CS**		**[174]CS**	**[167]CS**; **[176]CS**^**S**^	[176]CS		
**Other**	Nature Relatedness													[175]QE; [173]PP	

Study reference number and study design in brackets. Studies reporting an **unfavourable** association between the exposure and outcome are bolded. Studies reporting a **favourable** association between the exposure and outcome are bolded and underscored. Studies reporting no statistically significant association are not bolded.

*Study Designs*: CS = cross-sectional; QE = quasi-experimental; PP = pre-post test design.

*Where results differed for subgroups*: M = result for males; F = result for females

* = In study [170]CS, only high levels of gaming were associated with anxiety for males, while any amount of gaming was associated with anxiety for females; S = for ST exposure on school days only; ⁺ = In study [171]CS, low levels of computer use were better than no computer use, but high levels of computer use demonstrated no association with Total Difficulties.

*Screen Time Exposure Details*: IB = In Bed; SP = Screen Time During School Period; VP = Screen Time During Vacation Period.

*Psychological Outcomes*: ST = Short-term; LT = Long-term; SDQ = Strengths & Difficulties Questionnaire; HRQoL = Health-related Quality of Life subscale.

*Study Notes*: Studies [42]CS and [22]CS are described in text due to the non-linear nature of the results. Study [166]CS results refer to self-reported HRQoL–all associations were non-significant when parent-reported HRQoL.

Ten ST studies [162–166, 168–171, 176] and two GT studies [173, 175] reported no statistically significant association between at least one exposure and psychological outcome measured. Where statistically significant associations were reported, they were typically unfavourable for ST (*n* = 13 studies) and favourable for GT (*n* = 3 studies).

ST was mostly examined in relation to indicators of poor mental health for older adolescents. The results of studies demonstrating an association between ST exposures and psychological outcomes primarily suggest that high levels of ST are associated with poorer mental health across a range of exposures and outcomes. In particular, high ST was mostly associated with higher levels of depression/depressive symptoms [162, 164, 165, 168–170, 174] and anxiety/anxiety symptoms [165, 170, 176] for older adolescents. One study found a non-linear, U-shaped association between ST and mental health, whereby TV watching, gaming, using computers, and using smart phones above inflection points was associated with poorer mental health, but engaging with ST activities within moderate ranges appeared to be linked to mental well-being [22]. The only exception in the same study was weekend smartphone use, which was associated with poorer mental well-being at all usage levels [22].

Overall, the results for indicators of positive mental health were less clear; however, it appears as though certain activities, such as TV watching [163, 166, 171], were less important than others. For example, studies reported that high levels of video game playing were associated with lower emotional functioning [163], health-related quality of life [163, 171], psychosocial scores [163], and quality of life [171]. It was also associated with poorer school functioning for boys who videogamed more during school terms [166]. Contrastingly, one study suggested that more TV watching was associated with better health-related quality of life [171]. Studies seldom considered the impact of ST on cognitive functioning in this age group, with only one study suggesting an unfavourable association between gaming and attention for older adolescents [170]. Of the three studies examining academic achievement, most ST exposures, including social media use, were associated with poorer achievement [167, 174, 176].

Outdoor programs, camp experiences, and wilderness expeditions were investigated in this age group. While largely unrelated to most psychological outcomes, these GT experiences were found to increase self-efficacy [173] and positive identity [172], and decrease long-term total difficulties [175] and anxiety [173]. Another study [42] summarised 126 approaches to modelling pathways linking greenspace variables to mental health outcomes for adolescents, through a combination of single mediation, parallel mediation, and serial mediation analyses, highlighting the complexity of the relationship between the natural environment and mental health.

#### 3.2.5 Studies of mixed age groups

As previously mentioned, each study was allocated to an age group category; however, some studies included wide age ranges of participants, with no indication of a dominant age group. They were consequently classed as studies of mixed age groups [177–229]. Thirty-six studies with mixed age groups investigated ST as an exposure, while 17 such studies looked at GT as an exposure. Studies of mixed age groups comprised a total of 883,732 participants in ST studies, 68,783 participants in GT studies (plus 320 schools with unspecified student numbers), and 7,468 participants in studies exploring both ST and GT.

Individual characteristics of these mixed age groups studies can be found in S3 File. Results for these mixed age groups studies are presented in S4 File. Overall, few of these studies contradicted previously presented associations between ST, GT, and psychological outcomes. Results of mixed age group studies with a longitudinal, experimental, or intervention component are considered in more detail in sections 3.3.1 and 3.3.2 as they permit examination of causal linkages.

### 3.3 Exploring the basis for causal links

In exploring whether associations between ST, GT, and psychological outcomes are likely to be causal (Aim 2), elements of study designs and key variables used in analyses were considered (as outlined in section 2.4). Whether or not associations between ST, GT, and psychological outcomes are causal is an important question, for example, to justify investment in GT to promote psychological well-being. The key consideration is not the direction of causation, but whether there is evidence of causation. Psychological well-being may be affected by both ST and GT, and in turn psychological well-being may affect an individuals’ engagement with ST and GT to some degree. While bidirectionality offers opportunities for health promotion by intervening in the feedback loop, if the associations are artefacts produced by bias or confounding this would not be a worthwhile line to pursue for health promotion.

Although experiments and randomized controlled trials are upheld as the gold standard for demonstrating causation in psychology, they are not always feasible or ethical when investigating environmental exposures. As such, there has been renewed discussion in environmental epidemiology about how to make causal inferences from observational studies (e.g., [230–232]). There are previous examples of serious threats to health and the environment for which prudent action was delayed when, in hindsight, there were early warnings in observational data (e.g., the legacy of health (respiratory illness) and environmental (forest degradation) costs associated with sulphur contamination through ‘acid rain’[233]). This reflects a need to make best use of imperfect data when assessing relationships between the environment and human health.

Determining causation does not necessarily depend on a single method, but can involve integrating evidence from a range of methods and data sources; this has been framed as ‘triangulation of evidence’ [232]. If the majority of evidence points to the same conclusion, there is a strong likelihood that a relationship is casual. In this realm, it is valuable to pay attention to studies in which sources of bias are distinctive and potentially influence the outcome in atypical directions. With this in mind, despite making synthesis of evidence challenging, heterogeneity in the ways ST and GT were conceptualised and measured is a useful aspect of the literature. Likewise, the myriad of different contexts in which associations were examined is a strength, and provides some grounds for accepting that the associations are not artefacts, despite formal consideration of bias and confounding being erratic in this literature. Together, the abundance of findings and their relative consistency in terms of mostly favourable associations between GT and psychological outcomes and mostly unfavourable associations between ST and psychological outcomes, suggest that the associations are (a) not chance findings, (b) not attributable to publication bias (even though a degree of that may have occurred), and (c) possibly causal.

Family disadvantage remains the most important source of confounding and is likely to apply in almost all settings. Our planned focus on exploring differential impacts by SES within studies (Aim 3, section 3.4) represents both an assessment of confounding (addressed by stratification) and a question with social justice implications. However, before scrutinizing this aspect of the literature, we will provide an account of the studies that have a longitudinal, experimental, or intervention component, and consider how these support or oppose the case for causation. Cohort studies have the ability to demonstrate that an exposure is associated with an outcome that covaries over time. If the outcome variable is measured at baseline, then the *change* in outcome for different levels of exposure can be assessed, and a ‘dose-response’ effect can provide support for causation after addressing sources of bias and confounding. In theory, confounding is eliminated in intervention studies and experiments through random allocation of participants to groups. In practice, systematic differences may still be present, especially in relatively small studies, so it is important to examine whether groups were similar at baseline.

#### 3.3.1 ST studies

Nineteen longitudinal ST studies included in this systematic scoping review provided an indication of baseline psychological profiles and accounted for these appropriately in analyses (e.g., psychological profiles had been factored in, through using change from baseline or equivalent approaches). These studies permit examination of causal linkages between ST and psychological outcomes. A brief description of each study is provided below.

Two of these longitudinal ST studies considered associations with indicators of positive mental health. One demonstrated that ST was not associated with life satisfaction over a 6-month period for 10–17-year-olds, after controlling for baseline life satisfaction [127]. The other reported that computer use and recreational ST were associated with decreases in psychological well-being across 7^th^ grade [113]. One time-lag study assessed associations between ST and psychological well-being (as measured by self-esteem, life satisfaction, and happiness) for 8^th^, 10^th^, and 12^th^ grade students between 1991 and 2016 [197]. Using Granger causality analyses (which allows for assessment of whether the ST exposure changed before psychological well-being, or the converse), the study reported that increases in social media use, Internet use, texting, and gaming led to lower levels of adolescent psychological well-being over time [197].

Six longitudinal ST studies considering cognitive functioning had mixed findings. Three studies with follow up after one year reported that ST was associated with increased attention problems. These studies included 6–12-year-olds [106], 9–10-year-olds [87], and early adolescents [115]. One study using data for children from age 3–11 years reported no statistically significant association between ST and general cognitive functioning over time [201]. Two other studies reported varying results, both unfavourable and favourable, across different screen activities, genders, ethnicities, and specific cognitive tasks [224, 225]. Specifically, one study suggested that between the ages of 6–12 years, girls benefited cognitively from computer use more than boys, and Black children benefited more than White children [224]. Contrastingly, increased video game playing was associated with an improved ability to solve applied problems for Black girls over time, but was associated with reduced verbal task achievement for girls of all included ethnicities [224]. Over a 5-year follow up period, greater online communications and Internet use were detrimental to vocabulary and reading abilities for 10–18-year-olds in another study [225]. Contrastingly, computer gaming was associated with increased reading and problem-solving scores, particularly for girls and minority children [225]. Furthermore, greater computer use for studying was associated with increased test scores for girls but not boys in the same study [225].

Results were also inconsistent across seven longitudinal ST studies assessing indicators of poor mental health. When looking at outcomes assessed by the Strengths and Difficulties Questionnaire (SDQ), higher weekday computer use at approximately 4 years of age was associated with an increased risk of emotional problems in girls at age 6, while other screen activities were not associated with SDQ scores over time [205]. In a study of 14-year-olds, only TV viewing was associated with increased psychological difficulties (total SDQ scores) over a school year [113]. Two additional studies, assessing 5–7-year-olds [102] and 10–18-year-olds [192], reported differences in the longitudinal effects of various screen activities on SDQ scores. The study of 5–7-year-olds reported that higher TV watching time was associated with increases in conduct problems, but electronic game use was not associated with any SDQ scores over time [102]. On the other hand, the study of 10–18-year-olds found that higher computer and Internet use was associated with increased emotional problems, peer relationship problems, and total difficulties over time [192]. These studies suggest that different screen activities may affect different aspects of psychological functioning for children and adolescents of different life stages.

When considering measures of common psychological disorders, one study reported that initial ST at 13 years of age did not predict changes in depression or anxiety symptoms, and vice versa, up to approximately 20 years of age [182]. In a study of 12–16-year-olds, baseline videogaming and computer use were not associated with increased depression scores at 1-year follow up, but higher mobile phone use and television viewing were [178].

Mixed results were also reported in the five longitudinal ST studies which investigated academic achievement. One prospective study reported that for 10–14-year-olds, higher ST was associated with deteriorating school performance over 2 years [154]. Math achievement was reported to be negatively affected by TV [181, 204], communication-based ST [184], PC/Internet use [149] and total ST [149] across 4 studies. Measures of math achievement were not associated with Internet use [184, 204], video/computer game use [149, 181, 184], mobile phone use [149], texting, emailing, or instant messaging [184], TV/video time [149], or total ST [184] in the same studies. Contrastingly, Internet use [204] and watching/streaming TV shows or movies [184] were reported to be associated with greater math achievement in two studies. These studies mostly concerned adolescents [149, 181, 184], with one study following children from 4–8 years of age [204]. Follow up periods ranged from 3 [181, 184] to 6 [149] years.

Concerning reading and language subjects, watching/streaming TV shows or movies and surfing the Internet were reported to be associated with poorer achievement for high school students over 3 years [184]. Other ST activities such as video/computer games and communication-based ST [184], and Internet and TV time [204], were not associated with reading or language achievement for primary [204] or high school [184] students over time.

**Fig 4:**
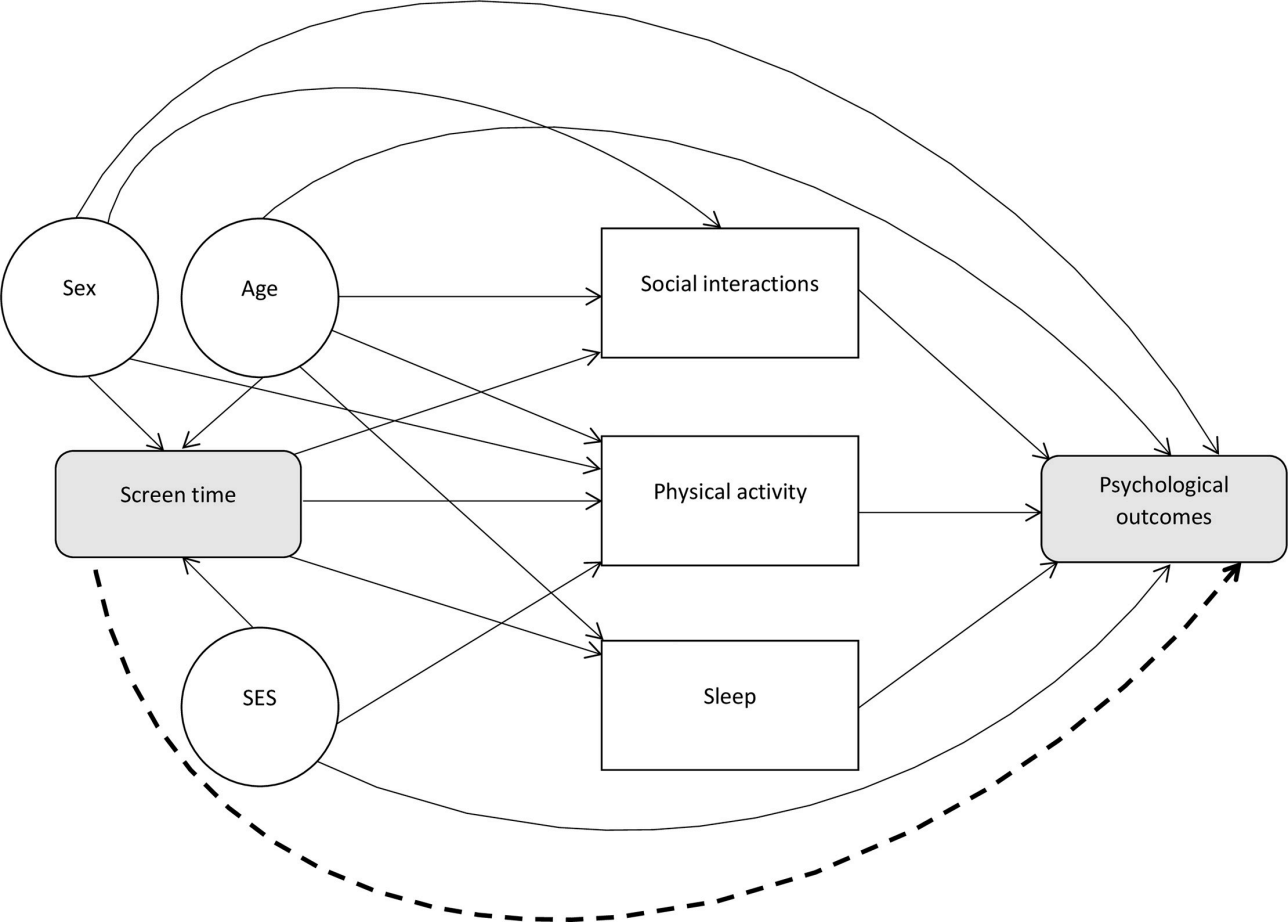
Pathways between ST and psychological outcomes (potential confounding and mediation by demographic and lifestyle variables).

In examining key variables used in ST analyses, a number of studies reported that poor sleep [68, 72, 87, 169], reduced physical activity [90, 118, 174, 203], and less in-person social interactions [67, 72, 197] were potential mediators between ST and a range of psychological outcomes. Furthermore, a number of studies reported that associations were found to differ by child sex [80, 88, 99, 106, 115, 117, 119, 128, 130, 134, 141, 143, 155–157, 164, 170, 174, 177, 183, 190, 196, 205, 214, 220, 224, 225] and age [85, 93, 115, 186, 215, 223]. As summarised in Fig 4, age and sex potentially confound associations as they independently affect both ST and psychological well-being, while the lifestyle variables are thought to be pathways through which elevated ST operates to impact on psychological well-being. Despite this evidence, these demographic (age, sex) and lifestyle variables were generally controlled (adjusted) for in analyses and examination of mediation or effect modification was limited. If simply controlled for, results will only reflect one pathway between ST and psychological well-being (as shown by the dashed line in Fig 4). This means that potential mechanisms and effect sizes of relationships could be concealed or diminished across the literature.

#### 3.3.2 GT studies

There were ten GT studies which permitted the examination of causal linkages (according to the criteria outlined in section 2.4) between GT and psychological outcomes in this systematic scoping review. Five were longitudinal GT studies which provided an indication of, and took into account, baseline psychological profiles. The other five studies had an experimental or intervention component, as well as equivalent comparison groups. A brief description of each study is provided below.

Two longitudinal studies examined psychological effects of nature in educational contexts. A prospective longitudinal study of 11-year-olds, including a comparison group with similar baseline profiles, reported that outdoor learning in a forest setting was associated with a steeper daily decrease in cortisol levels (e.g., superior profiles indicative of reduced stress) across the school year, when compared to traditional indoor classes [28]. A 4-year longitudinal study of young children found that attending a nature-based day-care centre (as compared to a conventional day-care centre) at age 3 years was associated with lower inattention/hyperactivity problems at ages 4 and 6 years [208]. Children exposed to high levels of outdoor time in day-care (as reported by day-care managers) also showed fewer inattention/hyperactivity problems at ages 4, 5, 6 and 7 years [208]. These children with higher levels of outdoor play showed significant declines in inattention/hyperactivity symptoms from age 3–5 years, retaining low levels at age 6 years, but increasing again as they entered school at 7 years of age. In the same study, children with low and high levels of outdoor hours in day-care did not differ in performance on the digit span task at age 3 years; however, the latter group showed consistently higher performance from age 4–7 years [208].

Three additional longitudinal studies, with a focus on incidental GT, contributed to the case for causal relationships through sound design and analysis. In a prospective cohort study, higher surrounding greenness in childhood/adolescence was associated with lower incidence of depressive symptoms later in life [209]. Stratified models suggested that this association was slightly stronger for young people with onset of depression before 18 years of age [209]. In another study, residential greenspace at age 4–5 years was associated with well-being at age 12–13 years [210]. Higher levels of well-being were associated with larger green space quantities; however, moderate quantities of greenspace, which were highest in quality, appeared to be most beneficial. Higher greenspace quality in early years was associated with lower internalising problems, but not externalising problems or total difficulties, in early adolescence. Furthermore, age-effects suggest that well-being benefits from greenspace quality seemed to intensify as children got older, while the well-being benefits gained from greenspace quantity seemed to weaken at age 10 years. In a pre-move post-move longitudinal study of 7–12-year-olds, moving to a new home environment with increased naturalness was associated with increased directed attention capacity post-move [219]. The change in naturalness score from previous to new home environments explained 19% of the variance in post-move attentional capacity, beyond the variance explained by pre-move attentional capacity [219].

Besides studies with a longitudinal component, several studies with an experimental or intervention component assessed the psychological effects of natural content in school environments, such as indoor plants [132] and green classroom views [187]. In the study looking at indoor plants, measures of academic achievement, anxiety, and well-being did not differ between 13-year-olds in non-randomised control and intervention classrooms which received indoor plants for a semester [132]. Contrastingly, in a randomised controlled experiment with comparable groups at baseline, natural classroom window views were associated with increased attention restoration and stress recovery for high school students [187].

Beyond the schoolyard, one study examined the psychological effects of walking in natural versus urban settings for children [73]; control groups were not used in this study as participants completed both study conditions. Following the nature walk, young children experienced increased spatial working memory and schoolchildren experienced increased attention, when compared to the urban walk [73]. A final study reported almost no differences in measures of well-being between high school students who participated in week-long outdoor adventure programs, and those who did not [175]. Despite being quasi-experimental, the results were considered reliable as matched control groups were utilised [175].

While a wider range of study designs were utilised in the GT literature there were methodological shortcomings in some studies which limit causal inferences. A lack of control group in pre-post studies [62, 75, 76, 133, 147, 172, 173, 222], significant baseline differences between groups [78, 108, 123, 145], and procedural issues [78, 89, 221] were common across studies. For example, the educational and well-being effects of education outside the classroom for schoolchildren may have been underestimated in three studies as the control groups were either ‘contaminated’ with education outside the classroom [78, 221], or the intervention schools had a pre-established interest in, and use of, education outside the classroom [89]. Furthermore, potential psychological effects of schoolyard greening interventions may have been underestimated in two studies, as students in a greening intervention school had higher baseline executive functioning than control school students in one study [139], while intervention students in another study reported liking their schoolyard to a greater extent at baseline than students in control schools [108]. Another study reported no difference in high school students’ scores on measures of self-esteem and mood after viewing natural environmental scenes and built environmental scenes on a screen [161]. Given the technologically-mediated GT experience presented in this study, it was difficult to compare with other studies offering full GT sensory experiences.

Studies frequently claimed that GT was associated with favourable psychological outcomes as a result of increased physical activity; however, this claim was not formally investigated in most instances. Only two studies demonstrated that the associations between various GT exposures and psychological outcomes were mediated by physical activity [42, 74], while two other studies reported that physical activity did not mediate associations between GT exposures and psychological outcomes [28, 85]. Relatively few studies performed adjustment for physical activity when this did not appear to be warranted. The association between superior cognitive functioning and higher GT was mediated by reduced traffic-related air pollution in one study [82]. Similar to the ST literature, associations between GT and psychological outcomes were found to differ by child sex [73, 77, 89, 173, 227] and age [73, 210], but in many studies these variables were often adjusted for rather than forming the basis for stratified analyses or investigation of interactions.

### 3.4 Exploring the extent to which associations hold across the spectrum of socioeconomic status

We were especially interested in exploring the influence of socioeconomic status (SES) on the associations between ST, GT, and psychological outcomes (Aim 3). On the one hand, as a marker of access to material and community resources and social support, SES could confound associations between high ST, low GT, and psychological well-being. It is also possible that these relationships may differ by SES, which could have important policy and social justice implications. Thus, careful attention to the role of SES is required in the design of studies and statistical analyses. An example of how confounding by SES could influence results was provided by a study which reported that being from a low SES background determined both whether a child’s preschool was classified as high or low quality on outdoor play environment categories and whether children had attention problems [95].

Overall, children and adolescents from low SES backgrounds were underrepresented in the included studies (see S3 File for indicators of SES in each individual study). Where studies reported on differences between participants and non-participants, participants were more likely to come from higher SES backgrounds [99, 105, 117, 160, 207, 223]. Participants lost to follow up, or excluded due to incomplete data, were commonly reported to be from low SES backgrounds, ethnic minorities, or families with lower education, employment, and income [65, 101, 102, 119, 121, 128, 130, 131, 140, 154, 155, 171, 192, 202, 228, 234–237]. In some cases data were available to show that lost or excluded participants also had poorer psychological outcomes, lower levels of physical activity, higher levels of ST, and lower levels of GT [101, 105, 115, 130, 154–156, 192, 226, 235, 238]. The feasibility of recruitment and follow up for longitudinal studies often motivates use of middle-to-high SES samples, with high quality data that is as complete as possible being sought [239]. This may be considered an advantage for the purposes of this systematic scoping review in that it limits the extent to which findings are confounded by SES, given the relative similarity of psychological profiles among middle-to-high SES youth. To explain, this is because the association between SES and psychological outcomes is not linear: there is a steep gradient between the lowest and next SES category in terms of psychological problems, but a flatter gradient across subsequent SES increments [240, 241].

On the other hand, the underrepresentation of children and adolescents from low SES backgrounds in this literature means that evidence to assess possible *differential* effects by SES is lacking. There were four ST studies and five GT studies which performed internal comparisons of children and adolescents from different SES backgrounds; these are considered below.

Overall, in these four studies high levels of ST appeared to have a stronger link with poor psychological outcomes for children and adolescents from low SES backgrounds. For example, one study reported that the association between high ST and poor SDQ outcomes was strongest for children in low income families [215]. Similarly, when primary analyses were stratified by SES, unfavourable associations between media consumption and self-regulation were strongest for toddlers from low SES families [70]. In another study, more television watching was associated with poorer math test scores only for students in the second lowest SES quartile [181]. Significant SES and racial differences in both ST and psychological outcomes were reported in a U.S. study, which in some cases led to differential associations between ST and psychological outcomes by SES [119].

Similar patterns emerged in the five GT studies which performed internal comparisons of children and adolescents from different SES backgrounds, with associations between GT and psychological outcomes appearing to be strongest for children and adolescents from low SES backgrounds. One study found that high SES was protective against the development of emotional problems for young children from age 3–5 years [85]. The same study reported that in the absence of socioeconomic advantage, neighbourhood greenspace could protect against the development of emotional problems [85]. Specifically, disadvantaged children with a higher percentage of greenspace in their neighbourhood had fewer emotional problems from age 3–5 years, relative to disadvantaged children in less green neighbourhoods [85]. Another study reported that living further away from a park was associated with worse mental health outcomes for 5 to 6-year-old children whose mothers had a low education level, but not for children whose mothers had a higher education level [203].

A study examining the psychological effects of an education outside the classroom program found that children from lower SES backgrounds had greatest improvements in SDQ scores, although this finding did not reach statistical significance which the authors attributed to a lack of power [78]. Contrastingly, in another study, time spent in outdoor play was associated with poorer school grades and higher conduct problems in 10–12-year-olds from low SES backgrounds [242]. That study reported that outdoor play was typically reflective of unstructured play when they were “not really doing any activities, just hanging around” [242]. When investigating associations between residential/school greenspace and academic performance, one final study reported that stratification by household income did not reveal any effect modification [212]. The study authors commented that low income families were underrepresented in their analytical samples, which may have led to underestimates of associations. Overall, these studies suggest there is a possibility that high levels of ST and inadequate access to, or time spent in nature, may disproportionately affect children and adolescents from low SES backgrounds. These findings are based on a limited number of studies and should be interpreted within those constraints.

### 3.5 Delineation of reciprocal effects of ST and GT on psychological outcomes

ST and GT appear to be associated with psychological outcomes in contrasting ways; ST is mostly associated with unfavourable psychological outcomes, while GT is mostly associated with favourable psychological outcomes. The *combination* of high ST and low GT observed in contemporary children and adolescents may be particularly harmful to their psychological well-being [19, 37]. As such, it is important to consider the reciprocal effects of both ST and GT on children and adolescents’ psychological outcomes (Aim 4).

Fourteen studies identified in this systematic scoping review measured both ST and GT [234–238, 242–250]. It is important to note that these studies did not necessarily measure both exposures with the intention of delineating the effects of high ST and low GT on psychological outcomes in children and adolescents, and it was not always possible to determine the reciprocal effects of both exposures. For the most part, these studies were interested in either ST or GT, with the alternate exposure being measured as a secondary variable.

Of the 14 studies, two provided some insight into associations between psychological outcomes and ST, in the presence of GT, and vice versa. In one pre-post study [244], German adolescents took part in a 10-day Outdoor Adventure Program, with no access to technology. They also self-reported the average daily time they typically spent on various screen activities in their leisure time. The study found that the psychological benefits gained from the outdoor adventure program were moderated by adolescents’ reported level of typical daily screen time (high (>3 hours/day) or low/moderate (≤3 hours/day)). Participation in the outdoor adventure program resulted in improved mental health across a range of measures for both low/moderate and high ST users but effect sizes were larger for high ST users, suggesting they may have reaped greater benefits from the outdoor adventure program. There was also a significant time by group interaction for life satisfaction scores, with increases in life satisfaction post-outdoor adventure program being significantly higher for adolescents who regularly engaged in high levels of ST. This illustrates potential psychological benefits of GT for high ST users in particular [244].

A randomised experiment of adolescents from England also provided potential to delineate the psychological impacts of ST and GT [243]. Participants completed a series of stressor tasks before being randomly assigned to an outdoor or indoor environment, with a friend or alone with a mobile phone. Following a period of rest in their assigned environment, participants completed a series of cognitive and mood measures. Attention restoration and positive affect was found to be greater for participants who rested in an outdoor environment, compared to those who rested in an indoor environment. Furthermore, being with a friend was found to be more beneficial than playing a game on a mobile phone. Self-reported attentiveness decreased more rapidly when playing on a mobile phone compared to being with a friend, but this only occurred for adolescents in the indoor environment. Being outdoors may buffer the psychological effects of playing on a mobile phone to some degree, but more research is needed to support this [243].

Three additional studies measuring both ST and GT allowed their reciprocal effects to be probed to some extent. One study demonstrated the psychological benefits offered by the outdoors, above and beyond physical activity, for Canadian early adolescents [236]. Survey respondents reported on their time spent (a) playing sedentary video games, (b) playing active video games, and (c) in active outdoor play. Isotemporal substitution models were used to estimate whether replacing time spent in sedentary videogames and active outdoor play, with active videogames, would be associated with changes in emotional problems, prosocial behaviour, and life satisfaction. The study found that active videogames were associated with better mental health than sedentary videogames, but active outdoor play was superior to active videogames. This provides some limited evidence to suggest that the association between ST and mental health goes beyond displacement of physical activity and that outdoor environments may provide unique benefits to mental health. Another study demonstrated that TV viewing was inversely associated with the compliant subscale of the Adaptive Social Behaviour Inventory, while outdoor play time was positively associated with the same measure (in the same model) for children aged 2–5 years [247]. Similarly, another study showed that TV watching on the weekend was inversely associated with health-related quality of life for children aged 9–11 years, while a range of greenspace indices (such as percentage of landscape and number of green patches within half-a-mile of children’s homes) were positively associated with health-related quality of life (in the same model) [237].

The analysis plans of the remaining nine studies did not entail delineating the reciprocal effects of ST and GT on psychological outcomes, and it was not possible to investigate this based on the results presented within these studies. Studies either assessed the effects of the exposures in separate models [235, 250], adjusted for either ST [234, 245, 249] or GT [238, 246] in analyses, or did not report relevant associations for determining reciprocal effects of ST and GT on psychological outcomes [238, 242, 248]. This highlights the complexity of gaining understanding of the reciprocal psychological effects of ST and GT. Studies reporting statistically significant associations typically found that independent associations between ST or GT and psychological outcomes were consistent with earlier findings (sections 3.2.1 to 3.2.4).

## 4 Discussion

We set out to collate and critically discuss the available literature on associations between ST, GT, and psychological outcomes in children and adolescents. The body of research has expanded greatly in recent years, especially in relation to ST, with the majority of available evidence coming from high-income countries. We identified 186 eligible studies for inclusion in the systematic scoping review and discuss our key findings below.

### 4.1 ST and GT have contrasting relationships with psychological outcomes

Many cross-sectional studies reported associations between ST or GT exposures with some, but not necessarily all, of the psychological outcomes assessed. There was no obvious pattern to the null findings and there were relatively few opposing results. What was clear was that higher ST tended to be associated with unfavourable psychological outcomes while greater GT tended to be associated with favourable psychological outcomes.

The longitudinal ST studies which permitted examination of causal linkages (according to the criteria outlined in section 2.4) were difficult to compare. However, observed statistically significant associations provided some support for unfavourable causal relationships, consistent with the multitude of cross-sectional studies. There were no experimental or intervention studies to draw on in relation to ST. For GT, in addition to some longitudinal studies which permitted examination of causal linkages, a small number of studies with an experimental or intervention component also demonstrated favourable relationships between GT and psychological outcomes, building the case for causal linkages.

### 4.2 There are limitations in existing study designs and analysis

Although there is a sizeable literature concerning ST or GT and psychological outcomes in children and adolescents, the majority of studies used cross-sectional designs. While the great volume and variety (e.g., heterogeneous ST and GT measures, diverse study samples and contexts) of cross-sectional studies is useful for demonstrating general consistency in results, the research now needs to move beyond this. Investment in study designs which permit examination of causal linkages is important for advancement of both fields.

Studies with a longitudinal component are an example of superior study designs. In particular, comprehensive longitudinal studies which take baseline psychological profiles into account and consider competing explanations are needed to understand the potential bi-directional and reciprocal relationships between ST, GT, and psychological outcomes [93]. In addition, more short-term intervention studies, preferably randomised controlled trials with comparable baseline groups, would be particularly persuasive in making the case for (or against) causality and could allow a better understanding of mechanisms [251].

In considering competing explanations, potential confounding and mediating variables should be treated appropriately in analyses (see Fig 4 in section 3.3.1). For children and adolescents of all ages, the displacement hypothesis was regularly put forth as a potential mechanism underlying unfavourable associations between ST and psychological outcomes. Displaced behaviours raised included getting adequate sleep [63, 67, 68, 71, 87, 100, 142, 146, 235], engaging in physical activity [142], experiencing in-person social interactions [83, 93, 99, 141], and dedicating time to academic activities [112, 121, 129, 158]. However, few studies examined mediation formally. Across the identified literature these important variables were frequently treated as confounders, despite their potential role on the causal pathway. Unless the aim is to isolate the direct independent effect of ST or GT, these factors should not be treated as confounders in analyses. Furthermore, exploration of effect modification by age, sex, and SES was relatively rare, despite the potential for these variables to interact with ST, GT, and psychological outcomes.

In addition to claims that enhanced protective behaviours (e.g., more physical activity and socialisation) operating through GT contributed to favourable psychological outcomes, many GT studies made appeals to the intrinsic qualities of nature that theoretically enhance psychological well-being. In particular, frequent reference was made to Kaplan’s Attention Restoration Theory [46], which postulates that spending time in nature can improve cognitive functioning by restoring direct attention abilities, enabling individuals to consequently perform better on tasks that depend on directed attention. Two intervention studies provided strong support for this, suggesting that outdoor education [28] and natural classroom window views [187] are beneficial for students’ attention restoration and stress recovery. Whether GT can assist in recovery of attention and reduction of stress following ST is not known, but is an interesting prospect.

### 4.3 Considering different developmental stages is important

This review highlights the importance of considering the way in which specific screen-based technologies and GT exposures affect children and adolescents, depending on social and biological factors unique to their developmental stage of life.

For example, for young children, cognitive and language development are profound. As such, ST was most commonly explored in relation to these domains in children under 5 years of age, and was typically associated with poorer cognitive and language development [63, 67, 68, 71]. These findings are possibly owing to displacement of parent-child interactions and reduced quantity and quality of child play [63, 67, 68, 71].

Early adolescence is another period defined by significant biological and social development. It is characterised by hyper-responsive neural reward systems [252], along with the pursuit of autonomy from family, and peer social acceptance [25–27, 253]; all in the absence of reliable behavioural inhibition [254] and reduced parental control. Therefore, the domains of greatest interest and the potential mechanisms proposed to link high ST to poor psychological outcomes in this age group are more complex than that of younger children. For example, it is proposed that social media, which is popular among adolescents, can contribute to poor mental health as it offers the opportunity for constant social comparison. Photographs on social media broadcast certain ideals and encourage young people to compare themselves to their peers with respect to their body image, life experiences, and abilities [113, 157]. This not only inflates social pressure to conform [113], but can also cause distress for young people when there are discrepancies between these publicised ideals and the self [157]. While real-world social acceptance has historically been open to interpretation for adolescents, social media overtly quantifies levels of social acceptance through numbers of “friends” and “likes” attained by users [255].

When considering the GT literature, associations between different types of GT and psychological outcomes were also dependent on the participant age group. For example, having access to private gardens or natural environments at home appeared to be important for pre- and school-aged children [85] (who are dependent on caregivers for access or transportation to public green spaces and parks) as it can increase opportunities for engagement in deep and complex play in nature, which is thought to be essential for healthy development [62]. In another study, greenspace *quantity*, over quality, was reported to be more important for young children [210]. Younger children may reap psychological benefits from large greenspaces as they afford the opportunity to socialise through group sports, games, and exploration, which are key for psychological well-being. However, given physical activity declines from childhood through to early adolescence, particularly for girls [256], the quantity of greenspace may become less important with older age.

Some evidence suggests that broader environments may be more important to early adolescents, who begin to gain a level of independence from their parents/caregivers. For example, greater neighbourhood greenspace was reported to buffer against perceived stress for early adolescents [124] and was associated with higher emotional well-being [160]. In another study, greenspace *quality* was reported to be more important than quantity for older children [210]. As mentioned above, physical activity declines from childhood to adolescence [256], while rumination may increase concurrently [257]. Therefore, high quality natural environments which are restorative (e.g., provide a feeling of ‘getting away’), may be more important for early-to-late adolescents because they provide opportunities for respite and mind-wandering [46].

Overall, little GT research related to cognitive functioning was available for early adolescents, and little GT research related to mental health was identified for older adolescents. Given early adolescence is a critical period associated with the development and consolidation of complex cognitive processes, and adolescence is a peak age for the emergence of common psychological disorders [258], more research in these areas is warranted.

### 4.4 Certain screen technologies are most relevant when considering psychological outcomes

A lack of consistency in the conceptualisation and measurement of ST considerably limits our ability to make detailed comparisons between studies, synthesise the existing evidence, and ultimately make broader conclusions. This includes varying measurement units (e.g., hours versus minutes of ST), and exposure variables being treated as either binary (e.g., high versus low ST) or continuous (e.g., minutes or hours of ST), with mixed data transformation methods and cut-off points (e.g., >2 hours ST per day) used across studies.

Historically, ST received attention as an important modifiable determinant of childhood obesity [259], which led to ST guidelines recommending that children and adolescents limit their ST to two hours per day [20] in order to reduce sedentary leisure time. Consideration of psychological impacts of ST invites further distinctions between types of ST, notably passive (e.g., television watching) versus interactive or stimulating ST (e.g., gaming, social networking), in view of their different psychological demands. For example, a recent systematic review reported that passive ST, like television watching, was less likely to be associated with poor sleep outcomes compared to more interactive screen-based activities including computer use, video gaming, and mobile device use [260]. Similarly, when TV exposure was assessed alone, it was mostly unrelated to psychological outcomes for adolescents in the studies included in this systematic scoping review.

ST within the included studies most commonly included television watching, followed by videogaming, and computer use. Not surprisingly, older studies do not feature contemporary interactive and stimulating technologies, such as portable small-screen devices like iPads, tablets, and smart phones. With approximately three quarters of adolescents now reporting smartphone ownership, and almost one quarter describing themselves as “constantly connected” to the Internet [261], future research should move towards focusing on the psychological impacts of these contemporary technologies which keep young people connected and make it difficult to ‘switch off’.

### 4.5 It is not clear what constitutes the most beneficial GT

Conceptualisations of GT in the included studies varied markedly. As per the ST literature, varying measurement units (e.g., Euclidean distance to greenspace versus greenspace within diverse buffer sizes), variables being treated as both binary (e.g., no exposure versus some GT exposure) and continuous (e.g., NDVI of greenness), with mixed data transformation methods and cut-off points used (e.g., 100m versus 500m buffers), once again limits our ability to make comparisons between studies, synthesise the existing evidence, and ultimately make broader conclusions. While some studies focussed on incidental exposure to urban greenspaces or residential greenness, others investigated the effects of more purposive exposure, such as outdoor play, private garden access, outdoor adventures, or education outside the classroom. It is important to note that residential proximity to greenspaces does not necessarily reflect use, and outdoor play is not guaranteed to take place in natural surroundings. Carefully planned studies are needed which determine whether incidental exposure to nature, and purposive use of natural spaces, yield similar psychological benefits. Currently, the literature fails to make a distinction between these GT exposures and the different psychological benefits they may afford individuals of different ages.

### 4.6 Youth from low SES backgrounds may be disproportionately affected

Children and adolescents from middle-to-high SES backgrounds were most commonly recruited and retained in studies. While this provides reassurance that findings are not driven by the SES gradient, current evidence pertaining to higher SES samples may be underestimating the psychological effects of ST and GT on young people as a whole. The use of high SES samples with higher baseline well-being may lead to ‘floor’ and ‘ceiling effects’, as was suggested in a study which reported non-significant findings related to well-being following an outdoor camp and wilderness experience with a sample of high SES adolescents [175].

As presented in section 3.4, in some studies the negative psychological effects of ST, and benefits gained through GT, have been found to be stronger in individuals from low SES backgrounds. On theoretical grounds, associations between ST and cognitive development may be particularly important for young children from low SES backgrounds. In combination with higher average ST [48–51], these children can experience lower levels of directed parental language [71], and may also face issues with neighbourhood safety, social isolation, and other life stressors which play a key role in parents’ decisions around media use at home [63] and access to local greenspaces [262]. Given the potential to provide community amenities in the form of additional green spaces, which could address some inequities in youth mental health, future research in this area should prioritise youth from low SES backgrounds.

### 4.7 There is value in considering both ST and GT in future research

Very few studies considering both ST and GT together were identified and included in this systematic scoping review. Given the lack of available evidence, it is difficult to determine whether individuals who demonstrate improvements in psychological functioning following exposure to a natural environment experience such improvements purely as a result of nature exposure, or whether reduced exposure to screen-based technologies in such environments contributes to their observed improvements. Equally, it is difficult to determine whether the psychological consequences of ST arise exclusively from the screen-based technologies themselves, or whether the observed psychological outcomes are also associated with the concurrent deficit in exposure to natural environments whilst an individual is engaging with screen-based technologies.

This lack of available evidence warrants further research which considers the psychological effects of both ST and GT on children and adolescents. Given the opposing ways in which technology and nature arguably influence the brain and human lifestyles, it is important to delineate their reciprocal effects to ensure accurate recommendations are made regarding appropriate ST and GT for optimal psychological well-being. Such delineation may assist in determining the ability of nature to act as a buffer against negative psychological effects of ST in a high-tech era.

On theoretical grounds, investigating the potential role of GT as an ameliorator to the consequences of extensive ST, is an interesting prospect. Paying constant directed attention to screen-based technologies can lead to directed attention fatigue. Attention Restoration Theory postulates that when direct attention mechanisms are fatigued, they can be restored in natural environments because they employ involuntary attention, which is not tiring or effortful [46, 47]. Similarly, Stress Reduction Theory contends that due to extensive human evolution in natural environments, modern humans may have a biologically prepared readiness to quickly and readily acquire restoration from stress in natural settings, but have no such preparedness for highly stimulating technological environments [45, 263, 264]. Given the psychological demands contemporary interactive and stimulating technologies place on children and adolescents, research looking at the restorative role of GT is warranted.

With an estimated 47% of total U.S. employment classified as at high risk of computerisation in coming years [265], modern technologies are here to stay, and it is important for young people to be tech-literate; however, determining activities which assist in preventing mental illness and promoting mental well-being, to ultimately reduce continued burden of youth mental health problems, is crucial. In a high-tech era, further research is required to properly measure and understand practical ways for ameliorating any detrimental impacts ST may be having on children and adolescents [255].

### 4.8 Limitations of the current systematic scoping review & recommendations for future research

A limitation of this systematic scoping review may be the inability to fully synthesise and systematically appraise included studies, due to substantial heterogeneity across included studies. However, it is important remember that the purpose of a scoping review is to describe the available literature broadly, including diverse study designs and methods with no requirement for an evaluation of the quality of the evidence.

Given our aim was to provide a broad overview of existing evidence, it was also beyond the scope of this review to discuss the magnitude of the effects of ST and GT on psychological outcomes. The disparate ways exposure variables were measured in the included studies made it difficult to make these comparisons. More focussed systematic reviews with meta-analyses should be undertaken in the future, to pool data for studies that conceptualised and measured exposures in similar ways. We believe that the body of evidence pertaining to outcomes such as depression, anxiety, psychological difficulties (as measured by the SDQ), attention, and academic achievement may be wide enough to allow the conduct of a focussed systematic review. Such a review, presenting magnitude of effects, would be beneficial in commenting on the practical and clinical significance of associations across the literature.

The current review focused upon ST duration rather than content; therefore, it was not possible to comment on the differential effects of specific content, such as violent videogames and educational TV programs. In general, recreational and educational ST were combined in study responses, which made it difficult to explore differential impacts. The Canadian Pediatric Society recently released new ST guidelines suggesting that ST content is equally as important as ST duration [266]; therefore, future research should aim to synthesise evidence reporting the effects of ST duration and content (specifically distinguishing between recreational and educational ST) on psychological outcomes in children and adolescents.

A further limitation is that the review was limited to articles published in English. We may not have identified all relevant studies, despite attempts to be as comprehensive as possible. This may be due to the inconsistent terminology used in describing and indexing ST and GT. For example, most studies sourced from reference lists were not captured in the original search because they referred to time in screen-based activities as ‘sedentary time’; however, as highlighted in the literature, sedentary time that is not spent using electronic devices has significantly different psychological effects than sedentary time spent with screens [148, 157]. Therefore, rather than considering screen time as an interchangeable term with sedentary time, as it typically is in obesity research, a distinction needs to be made in the literature when considering psychological impacts.

Despite the aforementioned limitations, the approach used in this study provides a comprehensive overview and description of the current state-of-the-evidence. Overall, we recommend that: (a) a focused systematic review of only studies with a longitudinal, experimental, or intervention component be undertaken in the future, (b) specific attention be paid to the psychological benefits of purposive versus incidental GT for children and adolescents of different ages, (c) interactive ST activities and different ST content be considered, and (d) derivation of effect magnitudes occur where studies can be pooled. Further, we recommend that starting dates for searches commence around the time when contemporary technologies, such as smart phones, were introduced. Older research pertaining to previous generations with older technology use and different socialisation patterns should be drawn on judiciously. In addition, a narrow range of operationalisations of ST and GT will need to be employed to limit heterogeneity and allow for more fine-grain analysis.

## 5 Conclusion

While moderate ST can be beneficial for young people in a connected world, it is widely speculated that the concomitant trends of increasing ST and decreasing GT among children and adolescents may be social determinants of trends in youth mental health problems. However, research rarely considers the reciprocal effects of extensive ST (which is arguably detrimental) and GT (which is arguably protective) on children and adolescents’ psychological well-being. Researchers should move beyond cross-sectional studies, to longitudinal and intervention studies which are designed to investigate the psychological effects of both ST and GT, with careful specification of the extent and type of exposure. Research should consider specific developmental ages of children and adolescents, young people from low SES backgrounds, and consider the specific contribution of other lifestyle variables. GT presents as a potentially novel strategy to ameliorate high levels of ST; however, robust evidence is needed to guide policies and recommendations for exposure at critical life stages in childhood and adolescence. Nature may currently be an under-utilised public health resource, and it could potentially function as an upstream preventative and psychological well-being promotion intervention for children and adolescents in a high-tech era.

## Supporting information

S1 File PRISMA checklist for scoping reviews. (DOCX)

S2 File Search strategies for the review. (DOCX)

S3 File Descriptive characteristics of studies included in the systematic scoping review. (DOCX)

S4 File Results from studies including mixed age groups. (DOCX)
