# Supplementary material for: Psychological impacts of “screen time” and “green time” for children and adolescents: A systematic scoping review
Source: PLoS One. 2020 Sep 4;15(9):e0237725. doi: 10.1371/journal.pone.0237725 (PMC7473739; doi:10.1371/journal.pone.0237725)
Supplement: S2 File — (DOCX) [file pone.0237725.s002.docx]

**S2. Search strategies for review**

**PubMed Logic Grid**

(#1 AND #4 AND #5 AND #6) – Both exposures

(#1 AND #2 AND #3 AND #5 AND #6) - Green AND Screen exposures

| **#1** | **#2** | **#3** | **#4** | **#5** | **#6** |
| --- | --- | --- | --- | --- | --- |
| **Population** | **Exposure: Green** | **Exposure: Screen** | **Exposure: Both** | **Outcome** | **Study Type** |
| child[mh] OR child*[tiab] OR adolescen*[tiab] OR teen*[tiab] OR adolescent[mh] OR youth*[tiab] OR “young people”[tiab] OR “young person”[tiab] OR parents[mh] OR parent*[tiab] | ecosystem[mh] OR ecosystem[tiab] OR biodiversity[mh] OR biodiversity[tiab] OR gardens[mh] OR garden*[tiab] OR Urbanization[mh] OR urbanization[tiab] OR urbanisation[tiab] OR “green space”[tiab] OR greenspace[tiab] OR “nature exposure”[tiab] OR “nature experience*”[tiab] OR Trees[mh] OR Tree*[tiab] OR greenness[tiab] OR NDVI[tiab] OR “normalized difference vegetation index”[tiab] OR “normalised difference vegetation index”[tiab] OR “public open space*”[tiab] OR “urban green space*”[tiab] OR “green area*”[tiab] OR “natural environment*”[tiab] OR forests[mh] OR forest*[tiab] OR wilderness[mh] OR wilderness[tiab] OR “connectedness to nature”[tiab] OR “nature immersion”[tiab] OR outdoor*[tiab] OR “nature relatedness”[tiab] OR ecotherapy[tiab] OR “attention restoration theory”[tiab] OR “stress reduction theory”[tiab] | “internet use”[tiab] OR “computer use”[tiab] OR Computers[mh:noexp] OR Computers, Handheld[mh] OR Cell Phone Use[mh] OR “cell phone*”[tiab] OR “mobile phone*”[tiab] OR “smart phone*”[tiab] OR Video Games[mh] OR “video game*”[tiab] OR Television[mh:noexp] OR television[tiab] OR TV[tiab] OR “screen time”[tiab] OR “screen use”[tiab] OR Social Media[mh] OR “social media”[tiab] OR “digital media”[tiab] | ecosystem[mh] OR ecosystem[tiab] OR biodiversity[mh] OR biodiversity[tiab] OR gardens[mh] OR garden*[tiab] OR Urbanization[mh] OR urbanization[tiab] OR urbanisation[tiab] OR “green space”[tiab] OR greenspace[tiab] OR “nature exposure”[tiab] OR “nature experience*”[tiab] OR Trees[mh] OR Tree*[tiab] OR greenness[tiab] OR NDVI[tiab] OR “normalized difference vegetation index”[tiab] OR “normalised difference vegetation index”[tiab] OR “public open space*”[tiab] OR “urban green space*”[tiab] OR “green area*”[tiab] OR “natural environment*”[tiab] OR forests[mh] OR forest*[tiab] OR wilderness[mh] OR wilderness[tiab] OR “connectedness to nature”[tiab] OR “nature immersion”[tiab] OR outdoor*[tiab] OR “nature relatedness”[tiab] OR ecotherapy[tiab] OR “attention restoration theory”[tiab] OR “stress reduction theory”[tiab] OR “internet use”[tiab] OR “computer use”[tiab] OR Computers[mh:noexp] OR Computers, Handheld[mh] OR Cell Phone Use[mh] OR “cell phone*”[tiab] OR “mobile phone*”[tiab] OR “smart phone*”[tiab] OR Video Games[mh] OR “video game*”[tiab] OR Television[mh:noexp] OR television[tiab] OR TV[tiab] OR “screen time”[tiab] OR “screen use”[tiab] OR Social Media[mh] OR “social media”[tiab] OR “digital media”[tiab] | Mental Health [mh] OR “mental health”[tiab] OR Mental Processes[mh] OR resilien*[tiab] OR Stress, Psychological[mh:noexp] OR Psychological Tests[mh] OR Depression[mh] OR depression[tiab] OR Anxiety[mh:noexp] OR anxiety[tiab] OR Happiness[mh] OR happiness[tiab] OR optimism[mh] OR optimism[tiab] OR Affect[mh] OR “positive affect”[tiab] OR “negative affect”[tiab] OR Cognition[mh] OR cogniti*[tiab] OR Educational status[mh] OR Achievement[mh] OR Underachievement[mh] OR Wechsler Scales[mh] OR Intelligence[mh] OR Attention[mh] OR attention[tiab] OR Executive Function[mh] OR “executive function*”[tiab] OR Mental fatigue[mh:noexp] | cross-sectional studies[mh] OR cross-sectional[tiab] OR Cohort Studies[mh] OR cohort[tiab] OR Case-Control Studies[mh] OR case-control[tiab] OR Longitudinal Studies[mh] OR longitudinal[tiab] OR GIS[tiab] OR “geographic information system*”[tiab] OR “geographical information system*”[tiab] OR epidemiologic studies[mh] OR systematic review[tiab] |

**PsycInfo Logic Grid**

(#1 AND #4 AND #5 AND #6) AND peer-reviewed journal.pt - Both

(#1 AND #2 AND #3 AND #5 AND #6) AND peer-reviewed journal.pt - Green AND Screen Exposures

| **#1** | **#2** | **#3** | **#4** | **#5** | **#6** |
| --- | --- | --- | --- | --- | --- |
| **Population** | **Exposure: Green** | **Exposure: Screen** | **Exposure: Both** | **Outcome** | **Study Type** |
| \|  \| (child* OR adolesen* OR parent* OR youth* OR "young people" OR "young person" OR teen*).ti,ab. \| \| --- \| --- \| | neighborhood.sh. OR neighbo?rhood*.ti,ab. OR Recreation areas.sh. OR exp "Nature (Environment)"/ OR exp Horticulture Therapy/ OR "horticulture therapy".ti,ab. OR ecology.sh. OR exp Urbanization/ OR urbani?ation.ti,ab. OR tree*.ti,ab. OR "green?space".ti,ab. OR greenness.ti,ab. OR NDVI.ti,ab. OR "normali?ed difference vegetation index".ti,ab. OR "public open space*".ti,ab. OR "urban green space*".ti,ab. OR biodiversity.ti,ab. OR forest*.ti,ab. OR "connectedness to nature".ti,ab. OR "nature immersion".ti,ab. OR outdoor*.ti,ab. OR "nature relatedness".ti,ab. OR ecotherapy.ti,ab. OR wilderness.ti,ab. OR restorati*.ti,ab. OR "attention restoration theory".ti,ab. OR "stress reduction theory".ti,ab. OR garden*.ti,ab. OR "green area*".ti,ab. OR "nature exposure".ti,ab. OR "nature experience*".ti,ab. OR ecosystem.ti,ab. | Computers.sh. OR exp Computer Games/ OR Computer Usage.sh. OR "computer use".ti,ab. OR Internet Usage.sh. OR "internet use".ti,ab. OR exp Mobile Devices/ OR Screen Time.sh. OR Social Media.sh. OR "screen time".ti,ab. OR "social media".ti,ab. OR TV.ti,ab. OR television.ti,ab. OR "smart?phone*".ti,ab. OR "cell phone*".ti,ab. OR "mobile phone*".ti,ab. | neighborhood.sh. OR neighbo?rhood*.ti,ab. OR Recreation areas.sh. OR exp "Nature (Environment)"/ OR exp Horticulture Therapy/ OR "horticulture therapy".ti,ab. OR ecology.sh. OR exp Urbanization/ OR urbani?ation.ti,ab. OR tree*.ti,ab. OR "green?space".ti,ab. OR greenness.ti,ab. OR NDVI.ti,ab. OR "normali?ed difference vegetation index".ti,ab. OR "public open space*".ti,ab. OR "urban green space*".ti,ab. OR biodiversity.ti,ab. OR forest*.ti,ab. OR "connectedness to nature".ti,ab. OR "nature immersion".ti,ab. OR outdoor*.ti,ab. OR "nature relatedness".ti,ab. OR ecotherapy.ti,ab. OR wilderness.ti,ab. OR restorati*.ti,ab. OR "attention restoration theory".ti,ab. OR "stress reduction theory".ti,ab. OR garden*.ti,ab. OR "green area*".ti,ab. OR "nature exposure".ti,ab. OR "nature experience*".ti,ab. OR ecosystem.ti,ab. OR Computers.sh. OR exp Computer Games/ OR Computer Usage.sh. OR "computer use".ti,ab. OR Internet Usage.sh. OR "internet use".ti,ab. OR exp Mobile Devices/ OR Screen Time.sh. OR Social Media.sh. OR "screen time".ti,ab. OR "social media".ti,ab. OR TV.ti,ab. OR television.ti,ab. OR "smart?phone*".ti,ab. OR "cell phone*".ti,ab. OR "mobile phone*".ti,ab. | Mental Health.sh. OR "mental health".ti,ab. OR exp Cognitive Processes/ OR exp Attention/ OR attention.ti,ab. OR "Depression (Emotion)".ti,ab. OR depression,ti.ab. OR Major Depression.sh. OR anxiety.sh,ti,ab. OR Generalized Anxiety Disorder.sh. OR Distress.sh,ti,ab. OR Well Being.sh. OR "well?being".ti,ab. OR Happiness.sh,ti,ab. OR Optimism.sh,ti,ab. OR "Resilience (Psychological)".sh. OR resilien*.ti,ab. OR exp Academic Achievement/ OR "academic achievement".ti,ab. OR "academic success".ti,ab. OR Intelligence.sh,ti,ab. OR exp Intelligence Measures/ OR exp Cognitive Ability/ OR cogniti*.ti,ab. OR Life Satisfaction.sh. OR (satisfaction adj3 life).ti,ab. OR Positive Emotions.sh. OR Negative Emotions.sh. OR "positive affect".ti,ab. OR "negative affect".ti,ab. | exp Cohort Analysis/ or cohort.ti,ab. or exp Longitudinal Studies/ or longitudinal.ti,ab. or intervention.sh,ti,ab. or "cross?sectional".ti,ab. or "case?control".ti,ab. or GIS.ti,ab. or "geographic information system*".ti,ab. or "geographical information system*".ti,ab. or RCT.ti,ab. or "randomi?ed controlled trial".ti,ab. or “systematic review”.ti,ab |

**Scopus Logic Grid**

(#1 AND #4 AND #5 AND #6) AND (Limit-to (SRCTYPE, “j”)) – Both exposures

(#1 AND #2 AND #3 AND #5 AND #6) (Limit-to (SRCTYPE, “j”)) - Green AND Screen exposures

| **#1** | **#2** | **#3** | **#4** | **#5** | **#6** |
| --- | --- | --- | --- | --- | --- |
| **Population** | **Exposure: green** | **Exposure: screen** | **Exposure: both** | **Outcome** | **Study Type** |
| TITLE-ABS ( child*  OR  adolescen*  OR  youth*  OR  parent*  OR  teen*  OR  {young person}  OR  {young people} ) | TITLE-ABS ( {green space}  OR  {green spaces}  OR  greenspace*  OR  {nature exposure}  OR  {nature experience}  OR  {nature experiences}  OR  tree*  OR  garden*  OR  greenness  OR  ndvi  OR  {normalized difference vegetation index}  OR  {normalised difference vegetation index}  OR  {public open space}  OR  {public open spaces}  OR  {urban green space}  OR  {urban green spaces}  OR  ecosystem  OR  biodiversity  OR  {green area}  OR  {green areas}  OR  {natural environment}  OR  {natural environments}  OR  forest*  OR  {connectedness to nature}  OR  {nature immersion}  OR  outdoor*  OR  {nature relatedness}  OR  ecotherapy  OR  wilderness  OR  urbanisation  OR  urbanization  OR  restorati*  OR  {attention restoration theory}  OR  {stress reduction theory} ) | TITLE-ABS ( {computer use}  OR  {internet use}  OR  {video game}  OR  {video games}  OR  videogame*  OR  television  OR  tv  OR  {screen time}  OR  {screen use}  OR  {social media}  OR  {digital media}  OR  {electronic device}  OR  {electronic devices}  OR  {cell phone}  OR  {cell phones}  OR  {mobile phone}  OR  {mobile phones}  OR  {smart phone}  OR  {smart phones}  OR  smartphone* ) | TITLE-ABS ( {green space}  OR  {green spaces}  OR  greenspace*  OR  {nature exposure}  OR  {nature experience}  OR  {nature experiences}  OR  tree*  OR  garden*  OR  greenness  OR  ndvi  OR  {normalized difference vegetation index}  OR  {normalised difference vegetation index}  OR  {public open space}  OR  {public open spaces}  OR  {urban green space}  OR  {urban green spaces}  OR  ecosystem  OR  biodiversity  OR  {green area}  OR  {green areas}  OR  {natural environment}  OR  {natural environments}  OR  forest*  OR  {connectedness to nature}  OR  {nature immersion}  OR  outdoor*  OR  {nature relatedness}  OR  ecotherapy  OR  wilderness  OR  urbanisation  OR  urbanization  OR  restorati*  OR  {attention restoration theory}  OR  {stress reduction theory} OR {computer use}  OR  {internet use}  OR  {video game}  OR  {video games}  OR  videogame*  OR  television  OR  tv  OR  {screen time}  OR  {screen use}  OR  {social media}  OR  {digital media}  OR  {electronic device}  OR  {electronic devices}  OR  {cell phone}  OR  {cell phones}  OR  {mobile phone}  OR  {mobile phones}  OR  {smart phone}  OR  {smart phones}  OR  smartphone*) | TITLE-ABS ( {mental health}  OR  depression  OR  anxiety  OR  well-being  OR wellbeing OR {well being} OR happiness  OR  optimism  OR  {life W/3 satisfaction}  OR  resilien*  OR  {positive affect}  OR  {negative affect}  OR  cogniti*  OR  {cognitive function}  OR  {cognitive functioning}  OR  attention  OR  {executive function}  OR  {executive functioning}  OR  {academic achievement}  OR  {academic success}  OR  intelligence ) | TITLE-ABS ( {systematic review} OR  cross-sectional  OR  rct OR {randomised controlled trial} OR {randomized controlled trial}  OR  cohort  OR  pre-post  OR  case-control  OR  {case control}  OR  longitudinal  OR  gis  OR  {geographic information system}  OR  {geographic information systems}  OR  {geographical information system}  OR  {geographical information systems}  OR  intervention ) |

**Embase Logic Grid**

(#1 AND #4 AND #5 AND #6) AND ‘article’/it - Both

(#1 AND #2 AND #3 AND #5 AND #6) AND ‘article’/it - Green AND Screen exposures

| **#1** | **#2** | **#3** | **#4** | **#5** | **#6** |
| --- | --- | --- | --- | --- | --- |
| **Population** | **Exposure:**  **Green** | **Exposure: Screen** | **Exposure:**  **Both** | **Outcome** | **Study Type** |
| Child/de OR “preschool child”/de OR “school child”/de OR toddler/de OR child*:ti,ab OR adolescen*:ti,ab OR teen*:ti,ab OR adolescent/de OR youth*:ti,ab OR “young people”:ti,ab OR “young person”:ti,ab OR parent/de OR parent*:ti,ab OR “school child*”:ti,ab OR “preschool child*”:ti,ab | Ecosystem/de OR ecosystem:ti,ab OR biodiversity/exp OR biodiversity:ti,ab OR garden*:ti,ab OR Urbanization/de OR urbanization:ti,ab OR urbanisation:ti,ab OR “green space”:ti,ab OR greenspace:ti,ab OR “nature exposure”:ti,ab OR “nature experience*”:ti,ab OR Tree/de OR Tree*:ti,ab OR greenness:ti,ab OR NDVI:ti,ab OR “normalized difference vegetation index”:ti,ab OR “normalised difference vegetation index”:ti,ab OR “public open space*”:ti,ab OR “urban green space*”:ti,ab OR “green area*”:ti,ab OR “urban area”/de OR “residential area”/de OR “suburban area”/de OR “natural environment*”:ti,ab OR forest/de OR forest*:ti,ab OR wilderness/de OR wilderness:ti,ab OR “connectedness to nature”:ti,ab OR “nature immersion”:ti,ab OR outdoor*:ti,ab OR “nature relatedness”:ti,ab OR ecotherapy:ti,ab OR “attention restoration theory”:ti,ab OR “stress reduction theory”:ti,ab | Internet/de OR “internet use”:ti,ab OR “computer use”:ti,ab OR Computer/de OR “Cell Phone Use”/de OR “cell phone”:ti,ab OR “mobile phone”:ti,ab OR smartphone/de OR “smart phone”:ti,ab OR “Video Game”/de OR “video game*”:ti,ab OR Television/de OR television:ti,ab OR TV:ti,ab OR “screen time”:ti,ab OR “screen use”:ti,ab OR “social media”/de OR “social media”:ti,ab OR “digital media”:ti,ab | Ecosystem/de OR ecosystem:ti,ab OR biodiversity/exp OR biodiversity:ti,ab OR garden*:ti,ab OR Urbanization/de OR urbanization:ti,ab OR urbanisation:ti,ab OR “green space”:ti,ab OR greenspace:ti,ab OR “nature exposure”:ti,ab OR “nature experience*”:ti,ab OR Tree/de OR Tree*:ti,ab OR greenness:ti,ab OR NDVI:ti,ab OR “normalized difference vegetation index”:ti,ab OR “normalised difference vegetation index”:ti,ab OR “public open space*”:ti,ab OR “urban green space*”:ti,ab OR “green area*”:ti,ab OR “natural environment*”:ti,ab OR forest/de OR forest*:ti,ab OR wilderness/de OR wilderness:ti,ab OR “connectedness to nature”:ti,ab OR “nature immersion”:ti,ab OR outdoor*:ti,ab OR “nature relatedness”:ti,ab OR ecotherapy:ti,ab OR “attention restoration theory”:ti,ab OR “stress reduction theory”:ti,ab OR Internet/de OR “internet use”:ti,ab OR “computer use”:ti,ab OR Computer/de OR “Cell Phone Use”/de OR “cell phone”:ti,ab OR “mobile phone”:ti,ab OR smartphone/de OR “smart phone”:ti,ab OR “Video Game”/de OR “video game*”:ti,ab OR Television/de OR television:ti,ab OR TV:ti,ab OR “screen time”:ti,ab OR “screen use”:ti,ab OR “social media”/de OR “social media”:ti,ab OR “digital media”:ti,ab | “Mental Health”/de OR “mental health”:ti,ab OR “psychological well-being”/de OR resilien*:ti,ab OR Stress/de OR “mental stress”/de OR Psychologic Test/exp OR Depression/de OR depression:ti,ab OR Anxiety/de OR anxiety:ti,ab OR Happiness/de OR happiness:ti,ab OR optimism/de OR optimism:ti,ab OR Affect/de OR “negative affect”:ti,ab OR “positive affect”:ti,ab OR Cognition/exp OR cogniti*:ti,ab OR “Educational status”/exp OR “academic achievement”/exp OR “Wechsler intelligence scale for children”/de OR “Wechsler preschool and primary scale of intelligence”/de OR Intelligence/de OR Attention/exp OR attention:ti,ab OR “Executive Function”/de OR “executive function*”:ti,ab | “cross-sectional study”/de OR cross-sectional:ti,ab OR “Cohort Analysis”/de OR cohort:ti,ab OR “Case Control Study”/de OR “case control”:ti,ab OR “Longitudinal Study”/de OR longitudinal:ti,ab OR “geographic information system”/de OR GIS:ti,ab OR “geographic information system*”:ti,ab OR “geographical information system*”:ti,ab OR “systematic review”/de OR “systematic review”:ti,ab OR RCT:ti,ab OR “randomized controlled trial”/de OR “randomised controlled trial”:ti,ab OR “randomized controlled trial”:ti,ab |
