# Supplementary material for: Psychological impacts of “screen time” and “green time” for children and adolescents: A systematic scoping review
Source: PLoS One. 2020 Sep 4;15(9):e0237725. doi: 10.1371/journal.pone.0237725 (PMC7473739; doi:10.1371/journal.pone.0237725)
Supplement: S3 File — (DOCX) [file pone.0237725.s003.docx]

# **S3. Descriptive characteristics of studies included in the systematic scoping review**

| **First author (publication year)** | **Country of sample** | **Study time-frame** | **Study design** | **Sample total N** | **Age group**  **(YC, SC, EA, OA)** | **Exposure**  **(ST, GT, or Both)** | **Psychological outcome**  **(AA, CF, PR, PS)** | **Indicator of socioeconomic status (SES)** | **Ref #** |
| --- | --- | --- | --- | --- | --- | --- | --- | --- | --- |
| Aggio  (2016) | United Kingdom | 2001 – 2013 | Longitudinal | 8,462 | Mixed  (YC & SC) | ST; parent-reported TV, videos, DVDs, computer, or video games time. | CF; British Ability Scales & Verbal Similarities Test. | - 42% of mothers had high academic qualification. - 76% of families had income above the poverty level. | 201 |
| Aggio  (2017) | United Kingdom | 2008 – 2009 | Cross-sectional | 13,169 | SC | Both; parent-reported TV time; parent-reported independent outdoor play. | PR & PS; parent-completed SDQ. | - 71% of families had income above the poverty level. | 248 |
| Agostini  (2018) | Italy | 2014 – 2015 | Longitudinal with comparison group | 93 | YC | GT; outdoor education at kindergarten. | CF & PS; teacher-report on Kuno Beller Developmental Tables. | - Unclear | 61 |
| Aguilar  (2015) | Chile | 2014 | Cross-sectional | 395 | EA | ST; self-report on typical daily television, computer, and video games time. | AA; grades in mathematics and language. | - 82% classed as middle or high SES. | 112 |
| Allen  (2015) | Australia | 2010 – 2014 | Longitudinal & Cross-sectional | 7,818 | Mixed  (SC & EA) | ST; parent-reported TV and electronic gaming time. | PR & PS; parent-completed SDQ. | - Non-English speaking families were underrepresented. - Those with incomplete data had a lower household income and neighbourhood socioeconomic position. | 215 |
| Amoly  (2015) | Spain | 2012 – 2013 | Cross-sectional | 2,111 | SC | GT; parent-reported play in greenspaces; residential and school surrounding greenness (NDVI); residential proximity to greenspaces; parent-reported annual beach attendance. | CF & PR; teacher-reported inattention (ADHD symptom criteria of DSM); parent-completed SDQ. | - 55% of mothers and 49% of fathers had university qualifications. - Neighbourhood SES mentioned but not reported. | 74 |
| Anderson (2017) | England, Wales, Scotland, Northern Ireland | 2001 – 2013 | Cross-sectional | 10,995 | Mixed  (YC & SC) | ST; parent-reported daily TV and video time. | PR; parent-completed Child Social Behaviour Questionnaire to measure self-regulation. | - 41.6% of original cohort not in analysis and were more likely to be from ethnic-minorities and households with less socioeconomic advantage. | 202 |
| Arora  (2018) | England | 2011 – 2014 | Longitudinal | 853 | Mixed  (EA & OA) | ST; self-reported use of TV, video games, mobile phones, computers, laptops, social networking before bedtime. | AA; score based off of English, Mathematics & Science records. | - Schools were selected to ensure different school types within different areas were included, which served as a proxy of socio-economic status. | 177 |
| Babic  (2017) | Australia | 2014 | Longitudinal | 322 | EA | ST; self-report on the Adolescent Sedentary Activity Questionnaire; daily recreational and non-recreational TV, DVD, computer, tablet, mobile phone time. | PR & PS; self-completed Flourishing Scale & SDQ. | - Majority of participants (58%) were from middle socioeconomic backgrounds (5^th^ & 6^th^ decile). | 113 |
| Bagot  (2015) | Australia | ? | Pre-PSt design | 550 | SC | GT; self-report on the Perceived Restorative Components Scale for Children to measure perceived restorativeness of school playground. | PR & PS; self-report on the Positive and Negative Affect Scale for Children. | - Schools across a broad range of socioeconomic groups. | 75 |
| Balseviciene (2014) | Lithuania | 2013 | Cross-sectional | 1,468 mother-child dyads | Mixed  (YC & SC) | GT; residential proximity to parks; residential greenness (NDVI). | PR; mother-reported SDQ. | - 80% of children’s mothers had a university/college degree. | 203 |
| Barton  (2016) | England | 2006 - 2012 | Pre-Post design | 130 | Mixed  (SC, EA & OA) | GT; wilderness expedition. | PS & Other; self-report on Rosenberg’s Self-Esteem Scale; self-report on State Connectedness to Nature Scale. | - Unclear. | 222 |
| Barton  (2015) | England | 2009 | Pre-Post intervention | 52 | SC | GT; nature orienteering intervention. | PS; self-report on Rosenberg’s Self-Esteem Scale. | - Participants were amongst the 20% most socio-economically deprived in England for one or a combination of factors, such as housing, income and health, crime and living environment. | 76 |
| Baumgartner (2014) | The Netherlands | ? | Cross-sectional | 523 | EA | ST; self-reported daily time and simultaneous use of TV, sending messages, social networking, using the computer, and playing video games, to measure media multitasking. | CF; the Dutch version of the Behaviour Rating Inventory of Executive Function measured executive function; The Digit Span measured working memory; the Eriksen Flankers task measured inhibition; the Dots-Triangles task measured shifting ability. | - Participants came from different levels of schools in both urban and rural areas. | 114 |
| Baumgartner (2017) | The Netherlands | ? | Longitudinal | 1,441 & 439 | EA | ST; self-reported daily time and simultaneous use of TV, sending messages, social networking, using the computer, and playing video games, to measure media multitasking. | CF; self-report on 9 symptoms for inattentiveness, adapted from the DSM-5 criteria for ADHD. | - Unclear. | 115 |
| Beere  (2017) | New Zealand | 2003 – 2012 | Cross-sectional | 230,929 | SC | GT; greenspace exposure measured by percentage of public and private greenspace within school parcel boundaries and zone buffers. | AA; New Zealand National Standards in mathematics, reading, and writing. | - Urban schools across all deciles of SES. | 77 |
| Benson  (2013) | United States | 2007 – 2008 | Cross-sectional chart review design | 117 | EA | ST; child/caregiver-report of total daily hours watching TV and/or using a computer/video game. | PR; self-completed Children’s Depression Index. | - SES not included in analysis. | 116 |
| Bezold  (2018) | United States | 1989 – 2013 | Prospective cohort | 11,346 | Mixed  (YC, SC, EA & OA) | GT; surrounding residential greenness (NDVI) during childhood and adolescence. | PR; McKnight Risk Factor Survey and Center for Epidemiologic Studies depression scale. | - 63% of participants had a household income equal to or above $75,000. - 65% of participant’s fathers had college level education. | 209 |
| Bickham (2015) | United States | 2009 – 2010 | Longitudinal | 126 | Mixed  (EA & OA) | ST; daily TV, video game, computer, and mobile phone use measured through survey questions, time use diaries, and ecological momentary assessment. | PR; self-administered Beck Depression Inventory for Primary Care. | - 45% of participants were not White. - 52% of participant’s parents had college level education. | 178 |
| Bolling  (2019) | Denmark | ? | Quasi-experiment | 631 | SC | GT; education outside the classroom. | PR & PS; student-completed SDQ. | - 75% from high SES backgrounds. | 78 |
| Booker  (2014) | United Kingdom | 2009 | Cross-sectional | 4,899 | Mixed  (SC, EA & OA) | ST; self-report of hours spent chatting on social networking sites, game console use, computer game use, and TV, video & DVD watching on a normal school day. | PR & PS; self-reported SDQ; self-report on 6 questions about happiness. | - More than 20% (weighted percentage) of the sample had parents with degree qualifications. | 223 |
| Borzekowski  (2005) | United States | 1999 – 2000 | Cross-sectional | 348 | SC | ST; parent- and self-report on average time spent watching TV, videos, and playing video games. | AA; mathematics, reading, and language arts sections of the Stanford Achievement Test. | - The sample was ethnically diverse. - 44% of students came from households where no parent had completed more than high school. - 28% lived in households where English was not the main language spoken. | 79 |
| Brodersen  (2005) | England | 1999 | Cross-sectional | 4,319 | SC | ST; self-report time spent watching TV or videos, playing video games or on the computer. | PR & PS; self-report on the Perceived Stress Scale, SDQ, and single self-rated health question. | - Schools varied by ethnicity and SES (independent (fee-paying) schools, schools from affluent outer London boroughs, and schools from more deprived inner-city areas). - The average deprivation score of the sample was more deprived than the U.K. population in general. | 80 |
| Brussoni  (2017) | Canada | 2014 | Pre-post design | 45 | YC | GT; Seven C scores to measure the quality of outdoor play spaces in childcare centres. | PR & PS; teacher-completed SDQ; teacher-completed Preschool Social Behaviour Scale-Teacher Form. | - The centres' outdoor play spaces scored lowest quality among 16 centres participating in previous research. | 62 |
| Busch  (2013) | The Netherlands | ? | Cross-Sectional | 2,425 | EA | ST; daily TV, computer, Internet, and video games time. | PR & PS; self-reported SDQ; self-report on Rosenberg’s Self-Esteem Scale; self-report on Schwarzer’s Generalised Self-Efficacy Scale. | - Students’ SES was reported to be higher than that of their peers in the Netherlands. | 117 |
| Cao  (2011) | China | 2010 | Cross-sectional | 5,003 | EA | ST; self-reported TV and computer time on usual weekday and weekend day. | PR & PS; self-report on Depression Self-Rating Scale for Children; self-report on Screen for Child Anxiety Related Emotional Disorders; self-report on School Life Satisfaction Rating Questionnaire for Adolescents. | - Low, medium, and high perceived SES. Proportions of sample not clear. | 118 |
| Carson  (2012) | United States | 2006 – 2007 | Cross-sectional | 6,700 | EA | ST; self-reported daily internet use, video game playing, computer game playing for weekdays and weekends. | AA & PR; self-report on the Social Skills Rating System; parent-reported SDQ; reading and mathematics achievement assessed in a one-on-one assessment. | - Majority White sample. - Poverty level and parental education level differed by ethnicity/race (White, Black, Asian American, Latino). | 119 |
| Casey  (2016) | Australia | ? | Cross-sectional | 494 | EA  (100% female) | ST; TV viewing, video game and computer activity. | PS; self-report on the Pediatric Quality of Life 4.0 Generic Core Scales for Teens aged 13-18. | - Sample from rural communities. - Majority of participants lived with both parents. - Most parents were employed and had more than 12 years education. | 120 |
| Casiano  (2012) | Canada | 2000 – 2001 | Cross-sectional | 9,137 | Mixed  (EA & OA) | ST; self-reported weekly TV/video watching, video game playing, and computer/Internet use over the past 3 months. | PR; symptoms of depression were measured with the Composite International Diagnostic Interview-Short Form. | - Majority of participants were White. - Majority of participants had middle-to-high household income. | 179 |
| Chen  (2014) | Australia | 2011 – 2012 | Cross-sectional | 3,353 | Mixed  (SC & OA) | ST; number of days students experienced 2 or more hours of ST (TV, computers, video games) per day outside of school hours over the past week; average screen hours across school and non-school days. | PS; the Child Health Utility 9D measured health-related quality of life. | - SES was close to average Australian levels as measured by the Index of Community Socio-Educational Advantage. | 213 |
| Corder  (2015) | United Kingdom | 2005 – 2007 | Prospective cohort | 845 | EA | ST; self-reported daily TV, video, DVD, internet, and video game time. | AA & PR; General Certificate of Secondary Education results at the end of Year 11; self-report on the Mood & Feelings Questionnaire. | - The sample had middle-to-high levels of socioeconomic advantage overall. | 121 |
| Corraliza  (2012) | Spain | ? | Cross-sectional | 172 | SC | GT; nearby nature in school and home areas measured by the Nearby Nature Observational Scale; child-report on the Perceived Nature Questionnaire to measure perception that the child has about the nearby nature in his/her surroundings. | PR; child-report on the Perceived Stress Scale; child-report on the Stressful Events Questionnaire. | - Unclear. ­­­ | 81 |
| Dadvand  (2017) | Spain | 2003 – 2013 | Longitudinal | 888 - 978 | Mixed  (YC & SC) | Both; residential surrounding greenness (NDVI); residential surrounding tree cover; time spent watching TV. | CF; Connors’ Kiddie Continuous Performance Test; Attentional Network Task. | - Participants in follow-up analyses had mothers with higher education than those who were lost to follow-up. | 234 |
| Dadvand  (2015) | Spain | 2012 – 2013 | Longitudinal | 2,593 | SC | GT; greenness surrounding home, commuting route between home and school, within and around school boundaries, and total surrounding greenness (NDVI). | CF; n-back test assessed working memory and superior working memory; Attentional Network Test assessed attention. | - The 36 schools were reflective of the general SES of schools across Barcelona. | 82 |
| Dalton  (2011) | United States | 2007 | Cross-sectional | 152 | Mixed  (SC & EA) | ST; self-reported TV, video game, and recreational computer time on school days. | PS; self-report on the Pediatric Quality of Life Inventory. | - The sample was quite disadvantaged socioeconomically and health-wise. | 216 |
| de Haan  (2004) | The Netherlands | 2001 – 2002 | Cross-sectional | 9,782 | Mixed  (EA & OA) | ST; watching TV and using the computer. | AA & PR; self-reported school results in Dutch, English and arithmetic; internalising and externalising problem behaviour. | - Unclear. | 180 |
| Dennison-Farris  (2017) | United States | 2015 | Cross-sectional | 121 | SC | ST; self-reported time spent watching TV, using a computer, playing sedentary electronic games, and playing non-sedentary electronic games on weekdays and weekend days. | PR; self-report on the Child Depression Inventory. | - Sample of American Indian youth. - Potentially more disadvantaged, but not explicitly reported. | 83 |
| Dettweiler  (2017) | Germany | ? | Longitudinal with comparison group | 48 | SC | GT; outdoor learning in a forest setting. | PR; salivary cortisol analyses, with samples taken at 8:30am, 10:20am, 12:30pm over the school year, to measure stress levels. | - Similar SES across both groups. - Unclear how advantaged the participants were. | 28 |
| Duch  (2013) | United States | ? | Longitudinal & cross-sectional | 119 (CS) & 73 (L) | YC | ST; parent- and child-reported 24-hour recall of screen media use on weekday, including TV, cell phones, DVDs, or computers. | CF; Ages and Stages Questionnaire: a Parent-Completed Child Monitoring System, Third Edition. | - All families had incomes below the poverty line. - Majority Hispanic. | 63 |
| Dumais  (2008) | United States | 2002 – 2004 | Longitudinal | 11,642 | Mixed  (EA & OA) | ST; self-reported weekday TV and video game time. | AA; 11^th^ grade GPA; 12^th^ grade maths scores. | - On average, participants were from SES quartile 2.6 (SD = 1.1). | 181 |
| Dumith  (2010) | Brazil | 1993 – 2005 | Cross-sectional | 4,431 | SC | ST; self-reported TV, video games, and computer time on weekdays and weekends. | PS; happiness faces scale. | - Adolescents across whole socio-economic spectrum were represented. - Numbers/proportions unclear. | 84 |
| Dunton  (2011) | United States | Across 4 days (Friday – Monday) | Ecological Momentary Assessment Study | 121 | Mixed  (SC & EA) | GT; time spent outdoors measured through ecological momentary assessment with mobile phones. | PR & PS; self-reported negative affect measured through 4 items; self-reported positive affect measured through 2 items. | - Low-to-middle income, ethnically diverse children in Southern California. | 217 |
| Dzhambov  (2018) | Bulgaria | 2016 | Cross-sectional | 399 | OA | GT; availability, access, quality, and usage of greenspaces was investigated; residential greenspace measured by NDVI, SAVI, and tree cover density; distance to nearest urban greenspace; self-reported perceived neighbourhood greenness, visible greenery from home, walking time to nearest greenspace, time spent in neighbourhood greenspace per week, and perceived neighbourhood greenspace quality. | PR; self-report on the General Health Questionnaire-12. | - A range of − 3.29 to 4.19 was observed on an individual level socioeconomic index. | 42 |
| Espinosa  (2006) | United States | 1998 – 2002 | Longitudinal | Unclear:  ~17,008 based on baseline 21,260 and attrition of 20% | Mixed  (YC & SC) | ST; parent-reported Internet use at home (yes/no) and TV time at home (between 3pm and dinner); teacher-rated computer proficiency. | AA; reading and mathematics achievement. | - Children are represented across the spectrum of SES. - Numbers/proportions unclear. | 204 |
| Esteban-Cornejo  (2015) | Spain | 2011 – 2012 | Cross-sectional | 1,146 | EA | ST; self-completed Youth Sedentary Behavior Questionnaire; average daily time watching TV/videos, playing computer/video games, internet surfing, doing homework/study with computer. | AA; math score, language score, and GPA from school records. | - 30% of participant’s mothers had a university level qualification. | 122 |
| Fagerstam  (2014) | Sweden | ? | Quasi-experiment | 86 | EA | GT; outdoor learning. | AA & PS; mathematics skills were assessed via a test; self-regulation skills were assessed with a Programme for International Student Assessment questionnaire. | - Unclear. | 123 |
| Feda  (2015) | United States | ? | Cross-sectional (GIS) | 68 | EA | GT; park access, park area, and housing density in adolescents’ neighbourhood. | PR; self-report on the Perceives Stress Scale. | - Overall, SES was approximately middle-class in the sample. | 124 |
| Feng  (2017) | Australia | 2012 | Cross-sectional  (GIS) | 3,083 | EA | GT; greenspace quantity assessed by percentage of land-use within each ‘statistical area 2’ of residence classified as “parkland” by the Australian Bureau of Statistics; parent-reported greenspace quality in the neighbourhood. | PR; parent-, teacher-, and self-completed SDQ. | - Appears children across the spectrum of SES were included. - Unclear on numbers/proportions of children in each SES tertile. | 125 |
| Feng  (2017) | Australia | 2004 – 2012 | Longitudinal | 4,968 – 3,798 | Mixed  (YC, SC & EA) | GT; greenspace quantity assessed by percentage of land-use within each ‘statistical area 2’ of residence classified as “parkland” by the Australian Bureau of Statistics; parent-reported greenspace quality in the neighbourhood. | PR & PS; parent-reported SDQ. | - Over 60% of children were from average-to-affluent socioeconomic backgrounds. | 210 |
| Ferguson  (2011) | United States | ? | Cross-sectional | 603 | EA | ST; self-report on Media use questionnaire, average weekly TV and video game time. | AA & CF; parent-report of adolescent’s GPA; child- and parent-completed Child Behavior Checklist to assess attention problems. | - Majority Hispanic sample. - Low average household income (compared to national average). - Reflective of the community. | 126 |
| Ferguson  (2014) | United States | 6-month follow-up | Cross-sectional & Longitudinal | 237 (CS) & 101 (L) | EA | ST; self-reported TV and social media time. | PR & PS; self-completed Zung Depression Inventory; self-completed Beck Anxiety Inventory; self-completed Satisfaction with Life Scale (5-item). | - Majority Hispanic sample. - Reflective of the community. | 127 |
| Finne (2013) | Germany | 2003 - 2006 | Cross-sectional | 6,813 | EA | ST; self-reported average daily TV/videos, computer/Internet, and gaming console use time. | PS; self-completed age specific versions of the revised German KINDL-R questionnaire, assessing health-related quality of life. | - Participants excluded due to missing data were more likely to be from low SES or immigrant backgrounds. | 128 |
| Flouri  (2014) | England | 2000 – 2007 | Longitudinal | 6,348 | SC | GT; neighbourhood greenspace assessed using the 2001 Generalised Land Use Database. | PR; parent-reported SDQ. | - The non-analytic sample were more advantaged than the analytic sample (because people in rural areas of England tend to be more affluent). - Maternal education and two-parent family structure were more common in those who dropped out. | 85 |
| Garcia-Hermoso  (2017) | Chile | 2014 | Cross-sectional | 395 | EA | ST; self-report of the number of hours per typical day in the past seven days spent watching TV, playing computer or video games, and other computer use. | AA; grades in mathematics and language core subjects | - 72% of participants were from a middle SES background. | 129 |
| Gentile  (2014) | United States | 7-month time period | Prospective study | 1,323 | SC | ST; self-reported TV, video game, and computer use time during different times of the day, separately for weekdays and weekends. | AA; teacher-reported average grade for each child. | - The average education of participants’ parents was some college level. | 86 |
| Barlett  (2012) | United States | 13-month time period | Prospective study | 1,323 | SC | ST; self-reported TV, video game, and computer use time during different times of the day, separately for weekdays and weekends. | CF; teacher-report on 3-items that measured attention problems. | - Unclear. | 87 |
| Godinho  (2014) | Portugal | 2003 – 2004 | Cross-sectional | 1,680 | EA | ST; self-reported TV and computer time on week and weekend days. | PR; self-report on the Second Edition of the Beck Depression Inventory. | - Those not included in analyses were more likely to attend public schools, have younger, less educated parents, and clinical scores indicative of depression. | 130 |
| Goldfield  (2016) | Canada | 2005 – 2010 | Cross-sectional | 358 | OA | ST; self-reported daily TV, sedentary video game, and recreational computer use hours. | PR; self-report on the Children’s Depression Inventory. | - 71% of sample was Caucasian. - Parental education mentioned but not reported. | 162 |
| Goldfield  (2015) | Canada | 2005 – 2010 | Cross-sectional | 358 | OA | ST; self-reported daily TV, sedentary video game, and recreational computer use hours. | PS; self-report on the Adolescent Core version of the Pediatric Quality of Life scale. | - 71% of sample was Caucasian. | 163 |
| Gopinath  (2012) | Australia | 2004 – 2011 | Cross-sectional & Longitudinal | 2,353 – 1,691 | Mixed  (EA & OA) | Both; self-reported daily hours watching TV, playing video games, and using a computer for fun; self-reported number of weekly hours in non-sporting outdoor activities. | PS; self-report on the Pediatric Quality of Life Inventory. | - Participants lost at follow-up were more likely to be East & South-East Asian. - Mix of public, private, or religious high schools. - Over 50% of parents had tertiary qualifications. | 235 |
| Greenwood  (2016) | England | ? | Randomised Experiment | 120 | OA | Both; flowing baseline measures, stressor tasks, and pre-treatment measures, participants were sent into either an outdoor or indoor environment for 20 minutes; in addition to the 2 environmental conditions, participants were randomised into 3 social contexts (alone, with a friend, of playing a game on a mobile phone). | CF & PS; attention was measured using the Necker Cube Pattern Control Task; mood and attentiveness were assessed using Zuckerman’s (1977) Inventory of Personal Reactions. | - College in South-West London. | 243 |
| Griffiths  (2010) | England, Wales, Scotland, Northern Ireland | 2005 – 2007 | Cross-sectional | 13,470 | SC | ST; mother-reported hours per day their child spent watching TV/videos/DVDs, used a computer, or played electronic games. | PR & PS; parent-completed SDQ. | - 89% of sample was White. - 59% of mothers employed. - ~30% of mothers had a diploma or degree. - 81% of households were non-lone mother. - Purposive overrepresentation of children living in disadvantaged areas and from ethnic minority groups, from Wales, Scotland, and Northern Ireland. | 88 |
| Gubbels  (2016) | The Netherlands | 2010 – 2012 | Longitudinal | 401 | EA | GT; perceived greenery, perceptions of greenery improvement, and greenery use following greenery interventions in districts. | PR; self-report on the Center for Epidemiologic Studies Depression Scale. | - 20 severely deprived districts in The Netherlands. | 131 |
| Gunnell  (2016) | Canada | 2006 – 2010 | Longitudinal & cross-sectional | 1,160 - 236 | Mixed  (EA & OA) | ST; self-reported hours per day typically engaged in TV viewing, video game playing, and computer use for weekdays and weekend days. | PR; self-report on the Children’s Depression Inventory; self-report on the Multidimensional Anxiety Scale for Children-10. | - 74% Caucasian. - 75% of parents (either one or both) had college-level education. | 182 |
| Gustafsson  (2012) | Sweden | 12-month timeframe | Quasi-experimental non-equivalent groups design | 230 | SC | GT; outdoor education in a green context. | PR & PS; parent-report on the SDQ. | - 83% of children came from middle-to-high SES backgrounds and none were from immigrant parents in the Intervention school. - 32% of children came from middle-to-high SES backgrounds and 65% were from immigrant parents in the Reference school. | 89 |
| Hamer  (2009) | Scotland | 2003 | Cross-sectional | 1,486 | SC | ST; parent-reported weekly TV and screen entertainment time for their child. | PR; parent-report on the SDQ. | - Sample spanned spectrum of SES, but numbers/proportions not reported. | 90 |
| Han  (2009) | Taiwan | 2005 – 2006 | Quasi-experimental control-series design | 76 | EA | GT; limitedly visible indoor plants were placed in classrooms. | AA & PR & PS; average term examination grades in Mandarin, English, mathematics, civil ethics, history, geography, chemistry, and physical education; self-report on the State Anxiety Inventory; self-report on Well-Being Measures by Kaplan (2001). | - Unclear. | 132 |
| Hartson  (2018) | United States | 2015 – 2016 | Cross-sectional | 40 | SC | ST; self-reported weekly hours watching TV, DVDs, videos, or playing on the computer or with video games. | PS; self-report on Rosenberg’s Self-Esteem Scale. | - 74% of children were from lower income households. - 80% of parents had high school level education, or lower. | 91 |
| Hayward  (2016) | Australia | 2014 | Cross-sectional | 3,295 | OA | ST; self-report on an item from the Core Indicators and Measures of Youth Health Survey; adolescents reported their ST for the previous seven days. | PR; self-report on the Moods and Feelings Questionnaire-Short Form. | - Sample had mid-range SES. | 164 |
| Herman  (2015) | Canada | 2011 – 2012 | Cross-sectional | 7,725 | Mixed  (EA & OA) | ST; adolescents reported how much time they spent on a computer playing computer games and using the Internet (outside of school/work), playing video games, and watching TV or videos, in a typical week over the past 3 months. | PS; self-rated health and mental health. | - 73% of sample was White. - 84% of households had < post-secondary / post-secondary graduate education level. | 183 |
| Hignett  (2018) | England | 2011 | Pre-post design | 40 | EA | GT; surfing program, fostering connectedness to the natural and marine environment. | PR, PS & Other; an adapted version of the Parent-Child Interaction System was used to monitor negative and positive affect in an interview; teacher-report on the Social and Emotional Aspects of Learning questionnaire; self-reported well-being on a relevant section of the Youth version of the British Panel Household Survey; self-report on an adapted and extended version of the Inclusion of Nature in the Self scale. | - Students had either been excluded from mainstream school or were at risk of exclusion. | 133 |
| Hinkley  (2014) | Belgium, Cyprus, Estonia, Germany, Hungary, Italy, Spain, & Sweden | 2007 – 2010 | Prospective cohort | 3,604 | Mixed  (YC & SC) | ST; parent-report on adapted questions from the Generation M-study; reported child’s TV viewing and e-game/computer use for weekdays and weekends separately. | PR & PS; parent-report on the SDQ; parent-report on the KINDL. | - Over 57% of children were from middle-to-high SES families. - 9% of children were from low SES families. | 205 |
| Hinkley  (2017) | Australia | 2008 – 2012 | Prospective cohort | 108 | Mixed  (YC & SC) | ST; parent-report of child’s week and weekend time spent watching TV, DVDs, videos, playing sedentary electronic games, playing active electronic games, and computer/internet use, over the past month. | PS; self-report on the Bar-On Emotional Quotient Inventory – Youth Version (short version). | - Childcare centres in low-, mid-, and high-SES areas were included. - 76% of participants were from a high SES family with a university-educated mother. | 206 |
| Hinkley  (2018) | Australia | 2013 – 2014 | Cross-sectional | 575 | YC | Both; mother-report of child’s TV, DVD, video, computer/electronic game/handheld device time for week and weekend days; mother-report of child’s outdoor play for week and weekend days. | PS; mother-report on the Adaptive Social Behavior Inventory. | - Selection of socioeconomically diverse local government areas in Melbourne. - 75% of the sample were from high SES and 22% were from mid-SES. | 247 |
| Hoare  (2014) | Australia | 2012 | Cross-sectional | 800 | EA | ST; self-report on the Adolescent Behaviours, Attitudes, and Knowledge Questionnaire; reported TV, video, DVD, videogame, and recreational computer use time on a single school day and weekend days. | PR; self-completed Short Moods and Feelings Questionnaire. | - 67% of sample were of European-Australian decent. - 56% of parents had completed tertiary education. | 134 |
| Hodson  (2017) | United States | 2010 – 2011 | Cross-sectional | 222 primary schools | SC | GT; urban environmental variables; **level of greenness, grass cover, shrub cover** based on the U.S. Geological Survey National Land Cover Dataset (NLCD) for 2011; the NLCD 2011 U.S. Forest Service **Tree Canopy** cartographic product; NLCD 2011 Percent Developed Imperviousness dataset to calculate mean **impervious surface** percent; the percentage of **waterbodies** using the USGS National Hydrography Dataset. | AA; school-level academic performance using third-grade reading and mathematics test scale scores, and the proportion of third grade students exceeding basic standards in reading and mathematics ability. | - A socioeconomically and demographically diverse population. - Approximately 10% living below the poverty line. - Numerous ethnic and racial groups are represented in school populations and vary greatly in their proportions from school to school. | 92 |
| Hofferth  (2010) | United States | 1997 – 2003 | Longitudinal | ~3,558 | Mixed  (SC, EA & OA) | ST; Time Use Diaries completed by parent or parent and child, asking about the child’s activities over a randomly designated 24-hour period; watching TV, playing video games (Game boy and other hand-held video game devices), playing on the computer, studying using the computer, computer communications (internet searching, accessing web sites, emailing, and instant messaging). | CF & PR; parent-report on the Behavior Problems Index; child-completed Woodcock-Johnson Revised Test of Basic Achievement (letter-word identification, passage comprehension, applied problems). | - 75% of children lived with 2 parents. - 44% of mothers had completed some college. - Two thirds of mothers were employed. - 72% of students were White. | 224 |
| Hofferth  (2012) | United States | 2003 – 2008 | Longitudinal | 1,221 | Mixed  (SC, EA & OA) | ST; child- and/or parent-completed time diaries measuring weekday and weekend computer game play, web site visits, email or instant messaging, study using the computer, video game play on hand-held devices or consoles, and television viewing. | CF; child-completed Woodcock-Johnson Revised Test (letter-word identification, passage comprehension, applied problems). | - Children from diverse ethnic/racial backgrounds are represented. - Numbers/proportions unclear. | 225 |
| Hrafnkelsdottir  (2018) | Iceland | 2015 | Cross-sectional | 244 | OA | ST; self-reported TV/DVD, Internet, and computer hours per day on average, separately for weekdays and weekends. | PR & PS; self-completed 22-item version of the Subscales of the Symptom Checklist 90; self-completed Rosenberg Self-Esteem Scale; self-completed Diener’s Satisfaction with Life Scale. | - 60% of mothers had university level education. | 165 |
| Hunter  (2018) | Canada | 2012 – 2015 | Longitudinal | 4,408 | Mixed  (EA & OA) | ST; self-reported average daily time watching/streaming TV shows or movies, playing video/computer games, surfing the internet, and texting, messaging, emailing. | AA; self-reported grades in Math and English. | - Students lost at follow-up were more likely to be from ethnic minorities. - 82% of sample was White. | 184 |
| Huynh  (2013) | Canada | 2009 – 2010 | Cross-sectional | 17,249 | Mixed  (SC, EA & OA) | GT; features of public natural space (total, green, blue) in 5km buffers around schools were obtained from the CanMap Route Logistics and Enhanced Points of Interests geographic information systems, including local parks and sport fields, provincial/territorial parks, national parks, other parks, wooded areas, campgrounds, picnic areas, golf courses, driving ranges, national wildlife and migratory areas, botanical gardens, and water bodies (oceans lakes, rivers, streams). | PS; self-completed Cantril ladder (a direct and global indicator of subjective wellbeing over time). | - 71% of the sample was Caucasian. - Over 92% of the sample had mid-to-high family affluence. - 71% of participants living in neighbourhoods with medium-to-high SES. | 226 |
| Iannotti  (2009) | United States & Canada | 2001 – 2002 | Cross-sectional | 22,053 | EA | ST; self-reported hours per weekday and weekend day using a computer (excluding homework), watching TV/videos. | PR & PS; self-reported somatic symptoms; self-rated Perceived Health Status; self-rated Life Satisfaction. | - Samples were representative of the United States and Canadian populations. - No further SES information provided. | 135 |
| Iannotti  (2009) | United States, Canada, Switzerland, the Netherlands, Czech Republic, Poland, Finland, Norway, Italy & Spain | 2005 – 2006 | Cross-sectional | 49,124 | Mixed  (EA & OA) | ST; self-reported hours per weekday and weekend day using a computer (excluding homework), watching TV/videos. | PR & PS; self-reported somatic symptoms; self-rated Perceived Health Status; self-rated Life Satisfaction. | - In the majority of countries, national representative samples were drawn and samples were stratified to ensure representation of relevant subgroups. - No further SES information provided. | 185 |
| Jackson  (2010) | United States | ? | Cross-sectional | 500 | EA | ST; self-reported typical number of days of Internet, videogame, and cell phone use for more than 3 hours; self-reported email and instant messaging frequency. | PS; self-completed Rosenberg’s Self-Esteem Scale. | - 67% of the sample were Caucasian Americans. - Children from high and low income households were represented, but numbers/proportions are not reported. | 136 |
| Jackson  (2011) | United States | ? | Cross-sectional | 482 | EA | ST; self-reported typical number of days of Internet, videogame, and cell phone use for more than 3 hours. | AA, CF & PS; self-reported school grades and GPA; reading and mathematics skills assessed using the Wide Range Achievement Test Revision 3; visual spatial skills assessed using the Wide Range Assessment of Visual Motor Abilities Section 2, Matching; self-completed Rosenberg’s Self-Esteem Scale. | - 67% of the sample were Caucasian Americans. - Parents of males were more likely to be in the lowest income level (32.9%) than were parents of females (21.2%). - African American parents (43.1%) were more likely to be in the lowest income level than were Caucasian American parents (18.7%). - Only 5.2% of African American parents were in the three highest income levels compared to 13.9% of Caucasian American parents. | 137 |
| Jackson  (2011) | United States | 3-year time period | Longitudinal | 482 | EA | ST; self-reported typical number of days of Internet and videogame use for more than 3 hours. | AA & CF; self-reported school grades and GPA; reading and mathematics skills assessed using the Wide Range Achievement Test Revision 3; visual spatial skills assessed using the Wide Range Assessment of Visual Motor Abilities Section 2, Matching. | - 67% of the sample were Caucasian Americans. - Parents of males were more likely to be in the two lowest income levels (30%, 37.5%) than were parents of females (19.3%, 29.3%). - Parents of African Americans were more likely to be in the two lowest income levels (38.6%, 48.6%) than were parents of Caucasian Americans (19%, 27.5%). - Parents of Caucasian Americans were more likely to be in the three highest income levels (10%, 2%, 1.5%) than were parents of African Americans (1.4%, 0%, 0%). | 138 |
| Jalali-Farahani  (2016) | Iran | ? | Cross-sectional | 465 | OA | ST; self-report on the Quantification de l’Activite Physique en Altitude Chez les Enfants questionnaire; total screen time, TV, and videogames/Internet hours per week, reported separately for during school period and vacation period. | PS; self- and parent-report on the Pediatric Quality of Life Inventory. | - Students were recruited from 3 different socio-economic zones in Tehran. - No further SES information provided. | 166 |
| Janssen  (2016) | Canada | 2014 | Cross-sectional | 20,122 | EA | Both; self-reported daily time spent playing active video games and sedentary video games; self-reported time spent playing outdoors outside of school hours. | PS; emotional problems measured via 9 questions designed for the study; self-rated life satisfaction using the Cantril Ladder; prosocial behaviour measured via 5 questions designed for the study. | - 77% of the sample was white. - 81% were Canadian born. - 80% of children lived in dual parent household. - 57% had high-perceived family wealth. - Excluded participants were more likely to be from ethnic minorities. | 236 |
| Kantomaa  (2016) | Finland | 1985 – 2002 | Cross-sectional | 8,061 | OA | ST; self-reported average daily TV viewing, working on a computer, and playing video games in hours. | AA; GPA calculated from grades in languages, mathematics, biology, geography, physics, chemistry, religion or ethics, history, music, visual arts, physical education, crafts, and home economics. | - 13% of mothers had higher education. | 167 |
| Katon  (2010) | United States | 2007 – 2008 | Cross-sectional | 2,291 | OA | ST; two questions about the hours and minutes spent on a computer and watching TV, that were adapted from a questionnaire used in a large survey of high school students. | PR; self-completed Patient-Health Questionnaire two-item depression scale. | - Household income mentioned but not reported for total sample. - 7% of sample was classified as a low-income household. | 168 |
| Kelz  (2015) | Austria | 2009 | Pre-post quasi-experimental field research | 133 | EA | GT; schoolyard greening intervention. | CF, PR & PS; executive functioning was assessed with the Attention Network Test (alerting score, orienting score, and conflict score); blood pressure was measured as an indicator of physiological stress; the Basler Well-Being Questionnaire was used to assess current well-being (intra-psychic balance); the Recovery-Stress Questionnaire was used to determine recovery from stress. | - Rural Austria. - Unclear. | 139 |
| Khan  (2018) | Bangladesh | ? | Cross-sectional | 671 | EA | ST; self-report on the Adolescent Sedentary Activity Questionnaire, reporting time spent watching TV or DVDs, using a computer, and social media during each typical school day and weekend day. | PR; parent-report on the SDQ. | - 52% of mothers and 71% of fathers had tertiary level education. - Family income was split relatively evenly across quartiles. - Those with missing data were more likely to report low education or income. | 140 |
| Khouja  (2019) | England | 1991 – 2010 | Longitudinal | 1,869 | OA | Both; self-report on six questions relating to average hours watching television, computer use, and texting for weekdays and weekend days; self-reported playing outdoors in Summer and Winter. | PR; self- and parent-report on the Pediatric Quality of Life Inventory. | - Participants lost at follow-up were more likely to have a mother with lower educational level. - 81-91% of children came from a “non-manual” family occupational social class. | 238 |
| Kim  (2016) | United States | ? | Cross-sectional | 92 | SC | Both; half-mile and quarter mile buffers were generated surrounding participant's homes, and various landscape indices were analysed; Percentage of Landscape, Number of Patches, Mean Patch Size, Mean Shape Index, Mean Nearest Neighbour Distance, and Patch Cohesion Index; total TV watching hours during the weekend, captured via the Physical Activity Questionnaire for Older Children. | PS; child- and parent-completed Pediatric Quality of Life Inventory, deriving psychosocial health summary score and total HRQOL score. | - The sample was composed of mostly Hispanic (83%), low-SES individuals. - 76% of children lived with both parents. - 49% of mothers were employed. - 22% of mothers had a college, vocational, or technical degree beyond secondary school. | 237 |
| Koivusilta  (2007) | Finland | 2001 | Cross-sectional | 7,292 | Mixed  (EA & OA) | ST; self-report of daily time spent on the computer for email, writing and surfing, playing digital games, and on mobile phone for texting, gaming. | AA, PR & PS; student's subjective assessment of his/her relative position in class based on preceding end-of-term school report; school status and type of school (not in school, vocational school, upper secondary school); 'educational career' was formed describing hypothesised educational prospects in adulthood; self-rated Health Status; self-reported Daily Health; self-report on 2 questions about Depression. | - 60% of participants were from middle-to-high SES backgrounds. | 186 |
| Kremer  (2014) | Australia | 2006 | Cross-sectional | 8,029 | SC | ST; self-report time spent watching television, on a computer, or playing video games for leisure, separately for week and weekend days in hours. | PR; self-report on the Short Mood and Feelings Questionnaire. | - Stratification by SES and rurality was conducted and communities were randomly selected from each stratum. - Within each community, a random sample of schools from the Catholic, independent and government sectors were represented across each state. | 93 |
| Kuo  (2018) | United States | 2009 – 2010 | Cross-sectional | 318 schools | SC | GT; greenness was measured in the School, the Catchment area (attendance boundaries for the school), and the Neighbourhood (the area inside the school catchment but outside the school zone); tree canopy cover and grass cover were captured for each area; Greenness variables were assessed by green cover data from the Chicago Urban Tree Canopy Assessment. | AA; School-level academic achievement; percentage of third graders at a school meeting or exceeding expectations in reading and math. | - Highly disadvantaged public elementary schools in Chicago. - 87% of third graders were eligible for free lunch - 45% were African-American, 43% Hispanic, and 3% Asian/Pacific Islander. - 26% spoke a language other than English at home. | 94 |
| Kweon  (2017) | United States | 2010 – 2011 | Cross-sectional | 219 schools | Mixed  (SC, EA & OA) | GT; Green Space was measured in schools, including trees, grass and shrubs, bare soil, paved surfaces, and buildings; schools were geocoded for analysis and the 2011 land-use/land-cover map of D.C. | AA; Percentage of students who received Proficient or Advanced academic performance scores in mathematics; Percentage of students who received Proficient or Advanced academic performance scores in reading. | - 66% of students were enrolled in the free lunch program. - 80% of students were African American, followed by Hispanic (12.04%), and white (6.18%). | 229 |
| Lacy  (2012) | Australia | 2005 – 2006 | Cross-sectional | 3,040 | EA | ST; assessed via questions adapted from the 2002 National Children’s Nutrition Survey conducted in New Zealand, which related to hours spent television viewing (including videos and DVDs), playing video games, and using the computer (other than for homework) over the last 5 school days and previous weekend. | PS; self-report on the adolescent module of the Pediatric Quality of Life Inventory 4.0 Generic Core Scales. | - 19% of participants were from areas with high SES. - 55% of participants were from middle SES areas. | 141 |
| Largo-Wright  (2018) | United States | 6-week period | Experimental cross-over | 37 | YC | GT; outdoor vs indoor classrooms. | PS; children completed a brief, self-reported ‘Face Scale’ survey after every writing lesson, to measure happiness; teachers completed an online survey at the conclusion of the study to measure perspectives on children’s happiness and wellbeing in the nature and control conditions. | - 85-88% of the students identified as White, non-Hispanic. | 64 |
| Lemola  (2015) | Switzerland | 2012 - 2013 | Cross-sectional | 362 | OA | ST; student-report on media consumption in bed before going to sleep on a regular school night; watching TV or movies, playing video games, talking or texting on the phone, and spending time online on Facebook or in chat rooms, or surfing the Internet. | PR; self-report on 6-items from the short version of the German version of the Centre of Epidemiological Studies Depression Scale. | - Unclear. | 169 |
| Li  (2016) | United States | ? | Randomised Controlled Experiment | 94 | Mixed  (EA & OA) | GT; one classroom had no windows, one classroom had windows which opened onto a built space, and the third classroom had windows which opened onto a greenspace. | CF & PR; subjective attentional functioning was assessed using a Visual Analogue Scale questionnaire; objective attentional functioning was assessed with the Digit Span Forward and the Digit Span Backward tests; subjective stress was assessed using a Visual Analogue Scale questionnaire; objective stress was measured via physiological measures. | - Diverse ethnicities included in sample, but numbers/proportions are not reported. - Schools were suburban, urban, and rural. - No SES information provided. | 187 |
| Liu  (2016) | China | ? | Cross-sectional | 13,659 | OA | ST; 2 items from the Youth Risk Behavior Survey were used as measures of ST; self- reported hours watching TV or playing videogames/computer use on a typical school day. | PR; self-report on the Center for Epidemiologic Studies-Depression Scale; self-report on the Multidimensional Anxiety Scale for Children; Youth Self-Report scale. | - The sample’s mean subjective social and economic status score (SD) was 6.1 (1.6; range, 1-the lowest to 10-the highest). | 170 |
| Maras  (2015) | Canada | 2006 – 2010 | Cross-sectional | 2,482 | EA | ST; Leisure-Time Sedentary Activities 6-item questionnaire was designed by the investigators; self-report hours per day in TV viewing, video game playing, and computer use on weekdays and weekends. | PR; self-completed Children’s Depression Inventory; self-completed Multidimensional Anxiety Scale for Children-10. | - At least one parent completed college for 87% of participants. - 72% of sample was Caucasian. | 142 |
| Markevych  (2014) | Germany | 2006 – 2009 | Cross-sectional | 1,932 | SC | Both; access to urban green spaces measured by the shortest distance between each child's place of residence and the nearest urban green space, with data obtained from the local Bavarian land use dataset; time spent outdoors during Summer and Winter; time spent in front of a screen during Summer and Winter. | PR; parent-report on the German SDQ. | - 77% of parents had high educational level. - 89% of children lived with 2 parents. | 249 |
| Markevych  (2019) | Germany | 1995 –  ~2014 | Cross-sectional | 2,429 | Mixed  (SC & OA) | GT; residential and school greenspace measured by NDVI, tree cover density, proportions of agricultural land, forest, and urban green space in buffers around addresses using a variety of GIS datasets; a combined home-school greenspace exposure was also created. | AA; parent-report of German and Maths grades at 10-years follow-up; self-report of German and Maths grades at 15-years follow-up. | - In the Munich sample, 73% of parents had high educational level & 83% of children lived with both parents. - In the Wesel sample, 42% of parents had high educational level, 50% had medium educational level, & 86% of children lived with both parents. - Participants with low SES and from immigrant families were underrepresented in analytical samples. | 212 |
| Mårtensson  (2009) | Sweden | 2004 | Cross-sectional with comparison | 198 | SC | GT; Outdoor Play Environment at Preschools measured with the OPEC instrument; the fraction of free sky above the play structures (i.e. sky view factor) was also assessed. | CF; teacher-rated on the Early Childhood Attention Deficit Disorders Evaluation Scales. | - 29% of mothers had high educational level. - 54% came from high SES background. | 95 |
| Martinez-Lopez  (2015) | Spain | 2011 | Cross-sectional | 2,293 | EA | ST; self-report on number of hours a day they watch TV and use the PC, for weekdays and weekends. | PS; Self-perceived health measured with a single item from the Health Behaviour in School-Aged Children Questionnaire; Well-being measured with a single item from the Health Behaviour in School-Aged Children Questionnaire. | - Unclear. | 143 |
| Mathers  (2009) | Australia | 2005 | Cross-sectional | 925 | OA | ST; use-of-time data were collected by a computerised activity recall diary, the Multimedia Activity Recall for Children and Adolescents (MARCA); adolescents completed 4 MARCA diaries (2 full school days and 2 full weekend days); the MARCA's was used to determine minutes (per recall) devoted to television viewing, using a computer, playing video games, and telephone use (talking/texting). | PR & PS; Global Health assessed by self-report on a single item from the Child Health Questionnaire; Health Status assessed by self-report on the Pediatric Quality of Life Inventory 4.0; Health-related Quality of Life assessed by self-report on the KIDSCREEN; self-report on the Kessler-10; self-report on the SDQ. | - Baseline sample came from areas of greater advantage than the analysis sample. - 31% of participants were in the most advantaged socioeconomic quartile. - 60% of participants were from middle SES areas. | 171 |
| Matin  (2017) | Iran | 2011 – 2012 | Cross-sectional | 13,486 | EA | ST; prolonged screen time was defined as watching TV, computer work and sedentary behavior (screen time in general) for more than 2 hours a day. | PS; Self-Rated Health was measured via a single question; participants indicated their degree of life satisfaction by using a ten-point scale. | - 66% of participants were from middle-to-high SES. | 144 |
| Matsuoka  (2010) | United States | 2004 – 2005 | Cross-sectional | 101 public schools | Mixed  (EA & OA) | GT; student exposure to nature at each school involved three groups of measures; the views of nature that students had from the school buildings were rated; vegetation levels on the campuses were measured; student potential access to this vegetation was determined. | AA; academic achievement was measured via the percentage of Michigan merit award winners, based on performance on the Michigan Educational Assessment Program test; graduation rates as reported to the state; the percentage of seniors stating that they planned to attend a four-year college upon graduation. | - Participant ethnicity and eligibility for free lunch program was considered, but numbers/proportions not reported. | 188 |
| McAnally  (2018) | New Zealand | ? | Pre-post with comparison | 104 | EA  (100% male) | GT; Outdoor Education Programme. | PS, AA & PS; self-completed Satisfaction with Life Scale; self-completed Rosenberg Self-Esteem Scale, self-completed SDQ; Students’ National Certificate in Educational Achievement marks for English, Maths, Science, and Social Studies; Creative Thinking & Problem Solving assessed with the Remote Associates Test (Mednick, 1962). | - Private boarding school. - 86% identified as New Zealand European. - Students from mostly high socio-economic communities (school decile rating of 9, where 10 represents schools with the lowest proportion of students from low socio-economic communities). | 145 |
| McCracken  (2016) | Scotland | 2014 | Cross-sectional | 276 | SC | GT; use of greenspace over the previous week assessed by self-report of type of green space used, frequency of use for each, and how often they had exercised outside in the previous week; Residential Greenspace data obtained from the Central Scotland Green Network and analysed with GIS. | PS; self-report on the Kid-KINDL questionnaire. | - On average, participants were from decile 6 (SD = 2.9) on the Scottish Index for Multiple Deprivation. | 96 |
| McCree  (2018) | England | 2013 – 2016 | Longitudinal mixed methods | 11 | SC | GT; Forest Schooling. | AA, PS & Other; Wellbeing, involvement, and engagement measured by session leader and researcher using Leuven scale measures; academic attainment measured by comparing students on national standards in reading, writing, and mathematics; Nature Connection measured with the Connection to Nature Index. | - Included a social mix of families, with 26% eligible for Free School Meals (national average = 26%). - Children who were 'struggling to thrive', were seen as likely to underachieve, and were economically and emotionally disadvantaged with special education needs, were chosen for the Forest School. | 97 |
| McDonald  (2018) | Canada | 2008 – 2010 | Cross-sectional | 1,596 | YC | ST; parent-report of child’s time per day on any type of media (television, movies, computer/tablet). | PR; parent-report on the Brief Infant-Toddler Social and Emotional Assessment. | - Mothers who joined and completed participation were more likely to have tertiary education, greater household incomes, & be Caucasian. - 78% of mothers had post-secondary qualifications. - 71% came from households earning ≥$80,000. - 82% of the sample was White/Caucasian. | 65 |
| McEachan  (2018) | England | 2012 – 2015 | Cross-sectional | 2,594 | YC | GT; residential greenspace calculated with the NDVI around participants’ geocoded home; subsample of respondents rated satisfaction with, and use of, local green spaces (public parks, sports playing fields, or other natural habitats; parents reported how many days their child played outside in green spaces per week in summer and winter, and how long on average (minutes per day); parents reported which greenspace they used most frequently in summer and were satisfied they were with its quality. | PR & PS; parent-report on SDQ. | - The study area, Bradford, is characterised by high levels of ethnic diversity and deprivation. - 58% of participants were of South Asian origin. | 66 |
| McHale  (2001) | United States | 2-year time period | Longitudinal | 198 | Mixed  (SC & EA) | Both; each year seven evening telephone interviews were conducted; children and parents reported daily activities outside of school and work hours, including outdoor play and watching TV. | AA & PR; school grades were obtained from  most recent report cards, and grade point averages were calculated from grades in math, science, social studies, and language arts; self-report on the Children’s Depression Inventory; mothers rated children's conduct using the 5-item conduct problems subscale from the Strengths and Vulnerabilities Questionnaire. | - Almost all families were White. - 90% of parents were employed. - Maternal education level was reported as a mean of 14.57 (SD = 2.17), with 12 representing high school graduate and 16 representing college graduate. - Paternal education was a mean of 14.67 (SD = 2.40). - Family background characteristics were variable, ranging from working to upper-middle class. | 242 |
| Mendelsohn  (2010) | United States | 2005 – 2008 | Longitudinal | 253 | YC | ST; 24-hour recall diary based on an interview with the mother; information about all electronic media (television, videos/DVDs, movies, and games) on the most recent typical day. | CF; language development assessed using the Preschool Language Scale-4. | - Bellevue Hospital Center, New York City, is an urban public hospital serving low SES families. - 94% of mothers were Latina. - Average maternal education level was completion of Grade 10. | 67 |
| Mireku  (2019) | England | 2014 – 2016 | Cross-sectional | 6,616 | EA | ST; adolescent-report of screen time 1 hour before sleep (mobile phone, tablet, eBook reader, laptop, portable media player, portable video game console, desktop computer, television or video game console) with the light on or in darkness. | PS; self-report of health-related quality of life on the KIDSCREEN-10. | - Majority of participants were White and had parents in a ‘higher’ occupation. - Majority of parents did not have higher education. | 146 |
| Mundy  (2017) | Australia | ? | Cross-sectional | 876 | SC | ST; parent-report how many hours their child spends watching TV or DVDs, playing video games (on computer or console (eg, Xbox)), and using the computer (e-mail/ schoolwork/internet access/chat), on school days and weekend days. | PR; parent report on SDQ. | - The sample was skewed to higher SES. | 99 |
| Mutz  (2019) | Germany | 2016 | Pre-test post-test | 76 | OA | Both; Outdoor Adventure Program - the campsite as well as the surrounding area has neither access to the internet and television nor service for mobile phones; self-reported daily leisure time screen time, in front of television, computer and game console in their on an average weekday and weekend day. | PR & PS; self-report on the Perceived Stress Questionnaire (subscales ‘worry’, ‘tension’, ‘joy’, ‘demand’); life aatisfaction assessed with single self-report item; hedonic balance measured according to Bradburn (2015). | - Participants predominantly represent the German middle-class. | 146 |
| Mutz  (2016) | Germany | 2015 | Longitudinal pre-test post-test pilot study | 12 | EA | GT; 9-day hike. | PR & PS; self-report on the Perceived Stress Questionnaire ("worry" & “demand” subscales); self-report on the General Self-efficacy Scale; self-report on the Mindful Attention and Awareness Scale; happiness and long-term life satisfaction measured with 2 self-report questions. | - Unclear. | 244 |
| Mӧßle  (2010) | Germany | 2005 | Cross-sectional & longitudinal | 5,529 | SC | ST; self-report of average daily time watching television or playing computer games for regular school day and regular weekend day; participants also completed a timetable for the day before the interview where they could mark on a 30 min basis to what extent they performed various activities (e.g., watching TV or DVD, playing computer games, etc). | AA; marks in German, Mathematics, Science, and physical activity were obtained via a teacher questionnaire. | - Majority of parents had high educational background. - Majority of participants were native to Germany. | 98 |
| Nakamura  (2012) | Japan | 2009 | Cross-sectional | 3,464 | SC | ST; questions pertaining to time spent using media (game, TV, and PC). | PR; subjective health complaints; measured by 9 items pertaining to depression, sleeplessness, ill at ease, dizziness, poor appetite, headache, abdominal pain, short-tempered, and negative thinking. | - Unclear. | 100 |
| Nathanson  (2018) | United States | ? | Cross-sectional | 402 | YC | ST; mother-reported how many hours the child uses a tablet or hand-held game player on a typical weekday and on a typical weekend day during the morning, the afternoon, and the evening. | CF; mother-report on questions from the short form of the Early Childhood Behaviour Questionnaire to assess temperamental EC. | - 80% of mothers were Caucasian. - 43% of mothers were employed. - On average, mothers had received some college education. | 68 |
| Nelson  (2006) | United States | 1994 – 1996 | Longitudinal | 11,957 | Mixed  (EA & OA) | ST; adolescents reported hours per week watching TV/videos, and playing video or computer games. | AA & PS; academic grades self-reported; self-report to 6 items modified from or similar to the Rosenberg Self-Esteem inventory. | - 70% of the sample was White. - 55% of parents had some college level education. - Mean household income was $45,000/year. | 189 |
| Nihill  (2013) | Australia | 2010 | Cross-sectional | 357 | EA  (100% female) | ST; self-report on the Adolescent Sedentary Activity Questionnaire (ASAQ); participants report time outside school, during week days and weekends, they spent watching TV/videos/DVDs, using computers for school and non-school purposes (e-games and e-communication). | PS; participants completed the physical self-concept and global self-esteem subscales from Marsh's Physical Self-Description Questionnaire. | - Girls from low-income communities. - Mean SES score of 4.3 (SD = 1.8), where 1 is more disadvantaged and 10 is most advantaged. - 85% of participants were Australian. | 148 |
| Norton  (2014) | United States | 2010 | Pre-test post-test | 159 | OA | GT; Wilderness Expedition. | PS; self-completed 40 Developmental Asset Profile, encompassing positive identity and self-esteem. | - Under-resourced urban teens. - 38% Hispanic, 25% African American and 9% Caucasian. - 37% of parents had a high school diploma and 20% had some college level education. | 172 |
| Ohannessian  (2009) | United States | 2006 – 2007 | Longitudinal & cross-sectional | 328 | Mixed  (EA & OA) | ST; adolescents indicated how much time they spent watching television, text messaging, e-mailing/IMing, playing video games (PlayStation, Nintendo, Game Boy, Xbox, etc.) or computer games, and “surfing the Web” on an average day. | PR; self-report on the Center for Epidemiological Studies Depression Scale for Children; self-report on the Child Anxiety Related Disorders scale. | - 41% were Caucasian, 22% were African-American and 24% were Hispanic. - 96% of mothers and 95% of fathers completed high school; 26% of mothers and 24% of fathers had completed college. - 52% of adolescents lived with both biological parents. | 190 |
| Otte  (2019) | Denmark | 2014 - 2015 | Quasi-experiment | 619 | Mixed  (SC & EA) | GT; education outside the classroom. | AA; mathematics skills were assessed using Hogrefe’s MG/FG test. | - 53% from high SES backgrounds. - 41% from middle SES backgrounds. | 221 |
| Page  (2010) | England | 2006 – 2008 | Cross-sectional | 1,013 | SC | ST; children reported how many hours they watched TV and played on the computer (not for homework) per day. | PR & PS; self-report on the SDQ. | - Participants who completed all data collection had lower deprivation scores compared to those who were excluded. | 101 |
| Parkes  (2013) | England, Wales, Scotland, Northern Ireland | 2005 – 2009 | Longitudinal | 11,014 | SC | ST; mother-report of child’s television/video/DVD viewing and computer or electronic game playing, outside school on weekdays. | PR & PS; mother-report on the SDQ. | - 90% of mothers were White. - 41% of mothers working. - Approximately 38% of mothers had higher education qualifications. - Those who dropped out were more likely to be from disadvantaged families. | 102 |
| Peiró-Velert  (2014) | Spain | 2010 | Cross-sectional | 3,006 | Mixed  (EA & OA) | ST; self-report on sedentary screen media use variables from the Adolescent Sedentary Activity Questionnaire; including TV/video/DVD viewing, computer for playing, computer for communicating, computer for doing homework, overall computer use, passive videogames, active videogames, mobile for communicating, mobile for playing. | AA; academic achievement or performance in the previous academic year. | - Spectrum of SES appears to be represented, but no numbers/proportions are reported. | 191 |
| Perry  (2012) | United States | 2000 | Cross-sectional | 371 | SC | ST; children reported how many hours they sat and watched television or videos, played video games, or used the computer yesterday. | PS; self-report on the Pediatric Quality of Life Inventory version 4.0. | - High concentration of minority residents and families with children living in poverty. - 57% of participants were Black and 40% were White. - 24% of participants experienced food insecurity. - 57% had annual income <$30,000. | 103 |
| Piccininni  (2018) | Canada | 2013 / 2014 | Cross-sectional | 20,697 | Mixed  (SC, EA & OA) | GT; students reported how many hours a day they usually spend time playing outdoors outside school hours and on weekends. | PR; self-report on an eight-item scale which asked about psychological (feeling low or depressed, irritability or bad temper, feeling nervous, and difficulties in getting to sleep) and somatic (headache, stomach ache, backache, and feeling dizzy) symptoms. | - There were no notable differences between those included and excluded with regards to ethnicity and perceived family wealth. - Of those included, 57% perceived their family wealth to be above average. - 80% of participants were from Canadian dominant culture. - 70% of participants came from neighbourhoods with medium-to-high capital. | 227 |
| Plitponkarnpim  (2018) | Thailand | 2014 - 2015 | Cross-sectional | 483 families | YC | ST; Information & Communication Technology exposure; daily screen time (TV, computer, tablet, smart phone, handheld game consoles) was parent-reported; 6-hour diary record blocks-recall input questionnaire at the clinic; prospective 1-hour blocks diary home recording. | CF; Capute Scales used to determine the presence of atypical development in cognitive development (visual-motor functioning) and expressive-receptive language. | - 61% of parents had a bachelor’s degree. - Over 60% of mothers worked. | 69 |
| Poulain  (2018) | Germany | 2011 – 2017 | Cross-sectional & longitudinal | 850 & 512 | EA | ST; self-report about the duration (hours) of the daily time spent with different screen-based media (TV/video, game console, PC/ internet, mobile phone). | AA; school grades were assessed for German, Mathematics, and Physical Education. | - 62% of participants belonged to the middle-class, while 22% to high-class. | 149 |
| Poulain  (2019) | Germany | 2011 – 2017 | Longitudinal | 814 | Mixed  (EA & OA) | ST; self-report of electronic media time per day using television/video, computers/Internet, and mobile phones. | PR & PS; behavioral difficulties assessed by the SDQ; quality of life derived from the KIDSCREEN-27. | - 62% of participants were from middle SES backgrounds, while 25% were from high SES backgrounds. - Compared to drop-outs, adolescents in analyses had higher SES. | 192 |
| Primack  (2011) | United States | 2003 – 2008 | Cross-sectional | 106 | EA | ST; media exposure data were collected using a cellular telephone-based EMA protocol; calls from a trained staff member during 5 extended weekends (Friday through Monday) in an 8-week period; participants were asked at every telephone call to identify any media they were using; they were specifically asked about (1) television or movies, (2) music, (3) video games, (4) Internet, (5) print media (magazines, newspapers, books). | PR; adolescent and parent interviews using the Schedule for Affective Disorders and Schizophrenia for School-Age Children-Present and Lifetime Version; a child psychiatrist provided a final diagnosis based on DSM-III-R or DSM-IV criteria. | - 89% of participants were white. | 150 |
| Przybylski  (2017) | England | ? | Cross-sectional | 120,115 | OA | ST; self-reported time watching films and other media (e.g., TV programs), playing games (e.g., on computers and consoles), using computers (e.g., Internet, e-mail), and using smartphones (e.g., social networking, chatting online) during free time. | PS; self-report on the Warwick-Edinburgh Mental Well-Being Scale. | - Nationally representative sample. - No specific numbers/proportions reported on levels of deprivation or ethnicity. | 22 |
| Radesky  (2014) | United States | 2001 –  ~ 2004 | Retrospective cohort study | 7,450 | YC | ST; parent-report how many hours their child spent watching TV and videos on a typical weekday and weekend day. | PR; caregivers completed the modified Infant Toddler Symptom Checklist. | - 46% of children were White, 16% were Black, 21% were Hispanic and 17% were Asian/Pacific Islander/Alaska Native. - 50% of mothers had at least some college level education. - 61% of mothers employed. | 70 |
| Reshadat  (2013) | Islamic Republic of Iran | 2012 | Cross-sectional | 573 | EA | ST; students reported daily time spent playing computer or video games. | PR & PS; student-report on the General Health Questionnaire. | - 58% of fathers had a diploma or higher diploma. - 47% of mothers had a diploma or higher diploma. | 151 |
| Richardson  (2017) | Scotland | 2005 – 2010 | Longitudinal | 2,909 | Mixed  (YC & SC) | Both; quantified the area of public parks and total natural space around each child's home; surveyed whether the child had access (sole or shared) to a private garden; hours of screen time per day. | PR & PS; parent/caregiver-report on the SDQ. | - In 38% of households, at least one person had achieved a degree qualification. - In 38% of households, at least one person had achieved a vocational qualification. - 23% of participants were from the most deprived neighbourhood areas. | 245 |
| Robinson  (2011) | Australia | 1989 –  ~ 2006 | Cross-sectional | 1,275 | EA | ST; adolescent reported their daily television/video viewing habits and computer use. | PR; parent report on the Child Behaviour Checklist for Ages 4 – 18. | - 91% of adolescents were Caucasian. - Other measures of SES, such as family income, were considered but numbers/proportions were not clearly presented. | 152 |
| Roe  (2011) | Scotland | 2007 | Pre-test post-test with comparison | 36 | SC | GT; Forest School. | PR & PS; a shortened 14-item version of the University of Wales Institute of Science and Technology Mood Adjective Checklist was used to measure participants’ mood, hedonic tone, energy, stress, and anger levels. | - Deprived urban areas of Central Scotland. | 104 |
| Rose  (2018) | Australia | 2014 – 2015 | Multisite pre-post design | 160 | OA | GT; Outdoor Programs. | PR & PS; students completed the Generalized Self-Efficacy Scale; the short form of the Ryff Well-Being Scales; 3 subscales (aggression, depression, and fear) from the Early Adolescent Temperament Questionnaire-Revised; the Nature Relatedness Scale. | - The sample was homogenous in terms of SES, which was considered high. | 173 |
| Rosen  (2014) | United States | ? | Cross-sectional | 1,030 | Mixed  (YC, SC, EA & OA) | Both; parent-report of child’s daily media and technology usage (going online, using a computer for other than being online, sending and receiving e-mail, IMing/chatting, talking on the telephone, texting, playing video games, listening to music, and playing with technological toys); parent-report of child’s daily outdoor play and exercise. | CF & PR; parent-report on the 18-item Attention Deficit Hyperactivity Disorder Rating Scale–IV–school version; parent and child attention symptomology checklist; Yale Single Item Depression Scale; parent-report on behavior problems in three items from the 11-item symptomology checklist. | - 39% of children were Latino/a, 22% were Caucasian, and 18% were Black/African-American. - 60% of parents were employed full time or part time (14%). - 41% of parents had a college degree and an additional 31% had some college. | 246 |
| Rosenqvist  (2016) | United States | 2005 – 2006 | Cross-sectional | 381 | SC | ST; parent-report of how many hours per day the child watches TV, or uses the computer for homework, playing games, Internet, or other). | CF; psychologist administered the NEPSY-11, measuring Attention and Executive Functioning, Language, Memory and Learning, Social Perception, and Visuospatial Processing. | - Those who did not participate were more likely to have parents with lower educational level. - 64% of mothers had college-level education. | 105 |
| Ruiz  (2010) | Spain | 2000 – 2002 | Cross-sectional | 1,820 | Mixed  (EA & OA) | ST; self-report of daily hours viewing television and playing video games. | CF; Spanish version of the "SRA Test of Educational Ability" to assess cognitive performance; verbal, numeric, and reasoning ability. | - SES considered in analyses, but numbers/proportions are not reported. | 193 |
| Rusby  (2014) | United States | 2009 – 2011 | Ecological Momentary Assessment (EMA) Study | 82 | EA | ST; participants completed four EMA assessment periods; students were prompted during non-school hours only; participants were asked about activities or behaviours they were doing, including small screen activities (being on the computer, watching television, or playing video games). | PR & PS; using a 1-to-9 scale, participants reported on their current mood states. | - 59% of participants were Caucasian, 16% Hispanic/ Latino, 5% American Indian, 2% Asian, 1% African American, 1% Hawaiian/Pacific Islander, 9% mixed race/ethnicity, and 7% unknown. | 153 |
| Russ  (2009) | United States | 2003 – 2004 | Cross-sectional | 54,863 | Mixed  (SC, EA & OA) | ST; parent-report of hours child spends watching TV, watching videos, playing video games, using the computer (not for schoolwork) on an average day. | PR & PS; parent-report of child’s general health status on the National Survey of Children’s Health; parent-report on one question about child social/emotional problems; parent-report on child’s self-esteem. | - Excluded participants were more likely to be from low-income families, to be of black or Hispanic race/ethnicity, and to come from households where the highest reported educational level was less than college. - 66% of the sample was White. - 70% of parents had educational attainment beyond high school. - 75% of children lived in 2-parent households. | 228 |
| Sanders  (2018) | United States | 2011 – 2012 | Longitudinal | 374 | Mixed  (EA & OA) | ST; youth- reported how many hours they spend in a typical day watching TV programs and playing video games. | PR; internalizing behavior problems were measured using depression and anxiety-related items representative of core symptoms in each domain. | - 65% of participants identified as white, 13% as African-American, and 22% as other or mixed race. - 70% of children were from dual-parent households. - Average income was $66,000 yearly. | 194 |
| Schutte  (2017) | United States | ? | Experimental crossover | 67 | YC & SC (stratified) | GT; children were randomised to walk in either an urban environment or a natural environment. | CF; Spatial Working Memory Task; Go-No Go Task (Wiebe et al., 2011); Continuous Performance Task (Wiebe et al., 2011); Digit Span Back Task (school-aged children only). | - A majority of the families were middle class. - 69% of participants were reported as Anglo-American, 7% were African American, and 24% did not report race/ethnicity. | 73 |
| Sharif  (2010) | United States | 2003 – 2005 | Longitudinal | 4,533 | EA | ST; self-report on how many hours adolescents watch TV, movies, videos, play videogames, on school days. | AA; self- and parent-reported school performance and grades. | - Participants who dropped out were more likely to be of non-white race and lower socioeconomic status. - 62% of participants were white, 18% Hispanic, 11% black, and 9% were other race. - 31% had a parent with a college degree. - Household income ranged from $10,000 or less (8%) to over $75,000 (30%). | 154 |
| Sharif  (2006) | United States | 1999 | Cross-sectional | 4,508 | EA | ST; self-reported hours adolescents watch TV and play videogames on weekdays and weekends. | AA; self-reported school performance. | - Those with missing data were more likely to have lower levels of parental education and come from lower SES schools. - For 78% of participants, both parents had completed high school. - 56% of schools were middle-to-high SES (based on free lunch programs). | 155 |
| Shiue  (2015) | Scotland | 2012 / 2013 | Cross-sectional | 1,997 | Mixed  (YC, SC & EA) | ST; parents reported children's daily TV and/or screen watching time in household interviews. | PR & PS; parent-report on the SDQ; self-rated health as either "good" or "fair to poor." | - Nationally representative sample. - No other SES information provided. | 211 |
| Soderstrom  (2013) | Sweden | 2009 | Cross-sectional with comparisons | 169 | Mixed  (YC & SC) | GT; outdoor environment quality at day care centres assessed by three persons using the Outdoor Play Environment Categories scoring tool; time spent outside was also measured for children. | PR; mid-morning and mid-afternoon saliva sampling to measure cortisol as an indicator of stress. | - Day-care centres with very low-quality environment and low SES were underrepresented. - Day-care centres were from 2 socio-economic regions – one being high/medium and the other being medium/low SES. - In high socio-economic areas, more mothers had post-graduate education. | 207 |
| Straker  (2013) | Australia | 2003 – 2006 | Cross-sectional | 643 | EA | ST; self-report Multimedia Activity Recall for Children and Adults; adolescents recorded their activities in a self-report recall electronic diary/questionnaire for a minimum of seven days (weekdays and weekends); including TV viewing, playing electronic games at a video game centre, using handheld electronic game devices such as Gameboy, using console devices such as PlayStation, different computer uses such as graphics, word processing, email, internet, gaming, and general. | PR & PS; self-report on Cowan's Perceived Self-Efficacy Scale; self-report on Beck's Depression Inventory for Youth; self-report on the Child behaviour Checklist. | - 95% of participants had at least one Caucasian parent and SES comparable with the general Australian population. | 156 |
| Strong  (2018) | Taiwan | 2001 – 2006 | Longitudinal | 3,795 | Mixed  (EA & OA) | ST; self-reported hours spent online gaming and online chatting/communicating per week. | PR; participants completed the Center for Epidemiologic Studies Depression Scale (modified for mental health-related studies of adolescents in Taiwan). | - Study used a multistage cluster sampling design to provide a nationwide representative sample in Taiwan. - On average, parents had completed high school level education. | 195 |
| Suchert  (2015) | Germany | 2014 | Cross-sectional | 1,296 | EA | ST; students reported how much time they spent on the most recent school day and the most recent Sunday with watching TV/DVDs, playing video/computer games (except active electronic gaming), other leisure-time pursuits on the computer/mobile phone. | PR & PS; self-report on the subscale “depressed affect” of the German version of the Center for Epidemiological Studies Depression Scale for Children; self-report on three items of the KINDL-R to measure self-esteem. | - 58% of participants attended a “Gemeinschaftsschule” or “Regionalschule” school, which tend to recruit students from low- to middle-class families. - 42% of participants attended a “Gymnasium” school, which serves mainly students from middle- and upper-class families. | 157 |
| Swing  (2010) | United States | 13-month period | Longitudinal & cross-sectional | 1,323 | SC | ST; parent and child reported average time spent watching TV and playing video games during 4 time periods (6AM - 12PM, 12PM = 6PM, 6PM - 12AM, 12AM - 6AM) on weekdays and weekends. | CF; teacher-report on 3 items that measure attention problems in the classroom. | - Unclear. | 106 |
| Syvaoja  (2013) | Finland | 2011 | Cross-sectional | 277 | EA | ST; screen time was evaluated with questions used in the WHO Health Behavior in School-Aged Children study; self-reported weekday and weekend hours watching TV and videos, playing computer or video games, or using a computer (for purposes other than playing games, for example, e-mailing, chatting, or surfing the Internet or doing homework). | AA; GPA was calculated and from subjects including native language, first foreign language, mathematics, physics/chemistry, biology, history, geography, religion or ethics, visual arts, music, and physical education. | - In 79% of families, the highest level of parental education was tertiary level education. | 158 |
| Syvaoja  (2014) | Finland | 2011 | Cross-sectional | 224 | EA | ST; self-reported daily hours on weekdays and weekends watching television/videos, playing computer or video games, using a computer (for purposes other than playing games, for example, emailing, chatting, or surfing the Internet or doing homework). | CF; cognitive functioning assessed using the Neuropsychological Test Automated Battery; visual memory assessed with a Pattern Recognition Memory test; executive functions assessed with Spatial Span and Intra-Extra Dimensional Set Shift tests; tests assessing attention were Reaction Time and Rapid Visual Information Processing. | - 71% of mothers and 56% of fathers had tertiary level education. | 159 |
| Tallis  (2018) | United States | 2012 | Cross-sectional | 495 schools | SC | GT; school surrounding greenness measured by the NDVI; agricultural lands in school surroundings; percentage of trees and shrubs around schools. | AA; California Standardized Testing and Reporting data on student achievement from 2012; standardized tests in science, mathematics and English language. | - Ethnicity and proportion of students eligible for a free lunch program were used as proxies for SES. - Numbers/proportions were not reported though. | 107 |
| Tillman  (2018) | Canada | 2011 – 2013 & 2016 | Cross-sectional | 851 | Mixed  (SC & EA) | GT; accessibility to nature (parks and water) was defined using Euclidean buffers at 500M around each child's home; NDVI was used to measure grass and shrubbery and dense vegetation in the buffers. | PS; child-report on the Pediatric Quality of Life Inventory 4.0. | - Almost 70% of participants living with 2 parents. - 62% of mothers and 52% of fathers had post-secondary education. - 62% of mothers and 68% of fathers were employed. - 67% of participants were from medium-to-high income households. | 218 |
| Tomopoulos  (2010) | United States | 2005 – 2008 | Longitudinal | 259 | YC | ST; 24-hour recall diary based on an interview with child’s mother; provided information about all electronic media (television, videos, DVDs, movies, games) the child had been exposed on the most recent typical day. | CF; cognitive development assessed using the Bayley Scales of Infant and Toddler Development-III; language development assessed using the Preschool Language Scale-4 (auditory comprehension and expressive communication subscales). | - Urban public hospitals serving at-risk families. - 41% of mothers were high school graduates. - Spanish was the primary language spoken for 86%. | 71 |
| Trinh  (2015) | Canada | 2009 | Cross-sectional | 2,660 | OA | ST; self-reported daily hours watching TV/movies, playing video/computer games, on a computer chatting, emailing, or surfing the internet, over the past 7 days. | AA, PR & PS; psychological distress was measured with the General Health Questionnaire; self-report on the Center for Epidemiologic Studies Depression scale; self-report on six items adapted from the Rosenberg Self-Esteem Scale; self-reported academic grades. | - The study sample was a highly dispersed distribution of over 100 schools including students from urban and rural schools and at all levels of socio-economic status. - On average, highest parental education was 14.6 years (SD = 1.71). | 174 |
| Twenge  (2018a) | United States | 2009 – 2015 | Surveillance: time-lag design | 388,275;  118,545 | Mixed  (EA & OA) | ST; in 2009, 2011, 2013 & 2015 self-report of average daily hours spent playing video or computer games or using a computer for something that is not school work (including activities such as Nintendo, Game Boy, PlayStation, Xbox, computer games, the Internet, Nintendo DS, iPod touch, Facebook, an iPad or other tablet, a smartphone, YouTube, or other social networking tools); self-reported social media use (visiting social networking websites); self-reported frequency of reading news on the internet; self-reported TV watching on average weekdays and weekends. | PR; self-report on 6-items from the Bentler Medical and Psychological Functioning Inventory depression scale. | - A nationally representative survey of 8th, 10th, and 12th graders. - 62% of adolescents were from higher SES. | 196 |
| Twenge  (2018b) | United States | 2006 – 2016 | Surveillance: time-lag design | 41,773 | Mixed  (EA& OA) | ST; Internet hours per week (not for school or work - e-mailing, instant messaging, gaming, shopping, searching, downloading music, etc); gaming hours per week (playing electronic games on a computer, TV, phone, or other device); texting hours per week; social media hours per week (visiting social networking sites like Facebook); video chat hours per week (video chatting (Skype, etc.); reading news online; TV viewing. | PS; self-report on the Rosenberg Self-Esteem Scale; self-report on a single item about life satisfaction; self-reported happiness. | - Race/ethnicity, SES, mother’s education considered in analyses, but numbers/proportions not reported. | 197 |
| Ulset  (2017) | Norway | 2006 – 2009,  2011 | Longitudinal | 562 | Mixed  (YC & SC) | GT; daycare managers reported daily hours children spent outdoors at daycare centres; daycare centers were also categorised as "nature-based" or "conventional.” | CF; teacher-report on the SDQ; children were tested with the Digit Span test, a subset of the Weschler Intelligence Scale for Children. | - 45% of mothers had some form of tertiary education, compared to 28% in Norway. - 84% of mothers and 96% of fathers were employed. - The median household income was approximately NOK 623 000. This was slightly lower than the national Norwegian average for households with children aged 0-5, but higher than the regional average for where the families lived. | 208 |
| Ussher  (2007) | England & Wales | ? | Cross-sectional | 2,623 | Mixed  (EA & OA) | ST; children reported daily hours they watched TV, videos, and played computer games. | PR; self-report on the SDQ. | - Majority of participants lived with both parents. - Parents typically worked in middle-to-high class occupations. | 198 |
| van Dijk-Wesselius  (2018) | The Netherlands | 2014 – 2016 | Quasi-experimental / prospective intervention study | 2,031 | SC | GT; schoolyard greening intervention. | CF & PR; Digit Letter Substitution Test to measure information processing speed (Natu & Agarwal, 1995); Sky Search task (a subscale from the Test of Everyday Attention for Children) to measure selective attention (Manly et al., 2001); emotional functioning assessed on the subscale emotional functioning of the Pediatric Quality of Life Scale. | - Intervention and control schools were carefully matched on socio-economic status. - No other SES information reported. | 108 |
| van Lier  (2017) | New Zealand | 2012 | Cross-sectional | 8,063 | Mixed  (EA & OA) | GT; gardening activity assessed with one item, "Do you or your family grow any of your own vegetables?" | PR & PS; student-report on the World Health Organization Well-being Index; measures positive mood, vitality, and general interests; student-report on the Reynolds Adolescents Depression Scale-Short Form. | - Diverse ethnicities. - Approximately 68% of the sample lived in areas which were middle-to-high SES. - 81% of participants lived in households with no individual-level poverty. | 199 |
| Verburgh  (2016) | The Netherlands | ? | Cross-sectional | 163 | SC  (100% male) | Both; participants indicated how many days per week and how many minutes per day they participated in outdoor play, TV-watching, computer use, and active gaming. | CF; motor inhibition measured with the Stop Signal Task; short term memory (verbal and visuo-spatial) assessed with the Digit Span Forwards task; working memory examined using the Digit Span Backwards task; modified version of the Attention Network Test used to measure alerting and orienting attention; modified version of the Flanker task to assess executive attention. | - Unclear. | 250 |
| Wang  (2019) | United States | 2009, 2011, 2013, 2015, 2017 | Surveillance study | 75,807 | Mixed  (EA & OA) | ST; self-report of daily hours playing video or computer games or using a computer for something that is not school work (Xbox, PlayStation, an iPod, an iPad or other tablets, a smartphone, YouTube, Facebook or other social networking tools, and Internet) on an average school day. | PR; self-reported psychological distress on three questions; poor mental health status was recorded if students answered yes to any of the three questions. | - Majority of the sample was White (>50% across each measurement year). - No other SES information reported. | 200 |
| Ward  (2016) | New Zealand | 2014 | Observational Study | 72 | EA | GT; greenspace exposure measured by GPS monitors worn by students for a 7-day period; access to publicly accessible parks, sports fields, and reserves was measured, but vacant land, school playgrounds, or backyards were not measured. | CF & PS; self-report on the Life Satisfaction Scale, 5 items derived from Hubener's Student Life Satisfaction Scale, the Ten Domain Index of Wellbeing, and a single item measure of happiness with life; 7 computerised neurocognitive tests were used to calculate performance in visual memory, verbal memory, processing speed, psychomotor speed, reaction time, cognitive flexibility, and executive function. | - Participating schools were all middle-to-high SES. | 160 |
| Wells  (2000) | United States | ? | Longitudinal (pre-move post-move design) | 17 | Mixed  (SC & EA) | GT; a naturalness subscale completed by a trained researcher, of a detailed objective Housing Quality Scale (Evans, Wells, Chan, & Saltzman, 2000); amount of nature in the window views as well as the material of the yard; completed for participants' home at phase 1, and for their new home in phase 2. | CF; mother-report on the Attention Deficit Disorders Evaluation Scale. | - Low-income urban children. - 64% of children were African American. | 219 |
| Wells  (2003) | United States | ? | Cross-sectional | 337 | SC | GT; naturalness scale of the residential environment was developed as part of a detailed housing scale instrument (Evans, Wells, Chan, & Saltzman, 2000); the amount of nature in the window view, the number of live plants indoors, and the material of the outdoor yard. | PR & PS; mother-report on the Rutter Child Behavior Questionnaire; child-report on the Global Self-Worth subscale of the Harter Competency Scale (Harter, 1982). | - 44% of children’s parents were single, divorced, or widowed. - 95% of participants were White. - 63% of mothers had completed some college. - Mean income-to-needs ratio for the families was 1.79 (SD = 1.66), where a ratio of 1.0 or below represents poverty. | 109 |
| Williams  (2018) | Australia | 2015 – 2016 | Quasi-experimental cross-over trial | 335 | OA | GT; outdoor adventure program. | PS & PR; self-report on the Generalised Self-Efficacy Scale, the Short Warwick Edinburgh Mental Well-being Scale, and the Basic Psychological Needs Scale-General.; self-report on the short-form of the State-Trait Anxiety Inventory, the 10-item Center for Epidemiologic Studies Depression scale, and the SDQ; Nature Relatedness measured with a 6-item shortened version of the Nature Relatedness Scale. | - The sample was relatively homogeneous in terms of SES (mean = 5.98, SD = 1.22), which was considered high (range 4–10, where lower scores equate to higher SES). | 175 |
| Wood  (2013) | England | 2009 | Randomised Controlled Trial | 25 | EA | GT; while completing a 10-minute cycling exercise, participants either viewed natural or built scenes on a projector screen. | PR & PS; self-report on the Rosenberg Self-Esteem Scale; self-report on the Adolescent Profile of Mood States Questionnaire. | - Unclear. | 161 |
| Wu  (2018) | Canada | 2003 – 2011 | Longitudinal | 4,861 | Mixed  (SC & OA) | ST; students reported the daily number of hours they spent playing computers or video games and watching TV. | PR; primary diagnosis of an internalizing or externalizing disorder obtained from health administrative data. | - 70% of parents had college or university level education. - Approximately 88% of participants came from middle-to-high income households. | 214 |
| Wu  (2014) | United States | 2006 – 2012 | Cross-sectional | 905 public schools | SC | GT; amount of trees and vegetation (greenness) in the vicinity of schools, measured by the NDVI. | AA; data from the Massachusetts Comprehensive Assessment System provided the school-based measure of student performance in English and Math. | - Approximately 65% of students were middle-high income. - 67% of participants were White. | 110 |
| Yan  (2017) | China | 2016 | Cross-sectional | 2,625 | OA | ST; students reported how many hours a day they usually spent watching television, playing e-games, receiving news or study materials from electronic devices, using social media sites or apps, and watching videos both on school days and on non-school days. | AA, PR & PS; self-reported scores on the last cumulative examination in their grade; self-report on the Middle School Student Mental Health Scale (developed by Wang) to assess anxiety; self-report on the Satisfaction with Life Scale; self-report on the Rosenberg Self-Esteem Scale. | - Unclear. | 176 |
| Yang  (2013) | Iceland | 2007 | Cross-sectional | 10,467 | Mixed  (SC & EA) | ST; students reported the average time they usually spent each day watching TV/DVD/VCR, playing Internet computer games, playing computer games not on the internet, using internet communication or 'chatting' channels, and 'other' computer use. | PR; self-report on the Symptom Check List 90 (little interest in doing things, little appetite, loneliness, that they cried easily or wanted to cry, had difficulties falling asleep or staying asleep, feeling sad or blue, or felt the future seemed hopeless). | - Population-based data, but SES not reported. | 220 |
| Zach  (2016) | Germany | 2005 – 2006 | Cross-sectional | 6,206 | SC | GT; parents reported accessibility of green space (availability of public parks or green spaces). | PR; parent-report on the SDQ. | - Approximately 90% of parents were working. - Approximately 69% of households had medium-to-high income. - Approximately 89% of children were raised by both parents. | 111 |
| Zhao  (2018) | China | 2016 | Cross-sectional | 20,324 | YC | ST; time spent on video programs, electronic games, and browsing the web via screen (including television, computer, cellphone, iPad, etc) on weekdays and weekends in the latest month was reported by parents. | PR & PS; parent-report on the SDQ. | - Approximately 50% of mothers had university level education. - 97% of children’s parents were still married. - Approximately 73% of children lived in middle-to-high income households. | 72 |

AA = academic achievement; ADHD = attention deficit hyperactivity disorder; CF = cognitive functioning; DSM = Diagnostic & Statistical Manual; DVD = digital video disc; EA = early adolescents (12 – 14 years old); GIS = geographic information systems; GPA = grade point average; GPS = Global Positioning System; GT = green time; HRQOL = health-related quality of life; NDVI – Normalized Difference Vegetation Index; OA = older adolescents (15 – 18 years old); PC = personal computer; PR = indicators of poor mental health; PS = indicators of positive mental health; SAVI = Soil Adjusted Vegetation Index; SES = socioeconomic status; SC = schoolchildren (5 - 11 years old); SD = standard deviation; SDQ = Strengths & Difficulties Questionnaire; ST = screen time; TV = television; VCR = videocassette recorder; WHO = World Health Organization; YC = young children (<5 years old).
