# Supplementary material for: Psychological impacts of “screen time” and “green time” for children and adolescents: A systematic scoping review
Source: PLoS One. 2020 Sep 4;15(9):e0237725. doi: 10.1371/journal.pone.0237725 (PMC7473739; doi:10.1371/journal.pone.0237725)
Supplement: S4 File — (DOCX) [file pone.0237725.s004.docx]

**S4. Results from studies including mixed age groups (ST = 36 studies; GT = 17 studies)**

|  | | **Screen Time Exposures** | | | | | | | | | | | | | **Green Time Exposures** | | | | | | | | | | | | | | | | | | | |
| --- | --- | --- | --- | --- | --- | --- | --- | --- | --- | --- | --- | --- | --- | --- | --- | --- | --- | --- | --- | --- | --- | --- | --- | --- | --- | --- | --- | --- | --- | --- | --- | --- | --- | --- |
|  | | 1 | 2 | 3 | 4 | 5 | 6 | 7 | 8 | 9 | 10 | 11 | 12 | 13 | 14 | 15 | 16 | 17 | 18 | 19 | 20 | 21 | 22 | 23 | 24 | 25 | 26 | 27 | 28 | 29 | 30 | 31 | 32 | 33 |
| **Psychological Outcomes Measured­** | | Active Electronic Games | Communication-Based Screen Time | Computer Use | Electronic Device Use - Leisure Time | Emailing/IMing/Texting | Gaming | Internet Use | Mobile Phone Use | Screen-Based Media Use/Screen Time | Sedentary/Passive Electronic Games | Social Media Use/Social Networking | TV Watching | TV/VCR/DVD Watching Time | Agricultural Land | Blue Space / Water | Classroom Features | Connection to Nature | Distance to City Parks from Residence | Education Outside the Classroom | Forest Area | Gardening Activity | Green Features in Environment | Greenspace / Parks | Views | Greenness (NDVI) | High Quality Outdoor Environment | Naturalness in the Home | Nature-Based Daycare Centres | Outdoors | Total Natural Space | Trees | Vegetation on Campus | Wilderness Expedition |
| **Indicators of Poor Mental Health** | Anxiety |  |  |  |  | **190CS**;  190L | **190CS^V,B^**;  **190L^V,B^**;  **190CS^V,G^**;  **190L^V,G^** | **190L**;  **190CS^Alc^** |  |  |  |  | **190CS^B^**;  **190CS^G^**;  190L |  |  |  |  |  |  |  |  |  |  |  |  |  |  |  |  |  |  |  |  |  |
|  | Anxiety Symptoms |  |  |  |  |  |  |  |  | **182CS**;  182L |  |  |  |  |  |  |  |  |  |  |  |  |  |  |  |  |  |  |  |  |  |  |  |  |
|  | Conduct Problems (SDQ) |  |  | 192L^I^ |  |  |  |  | 192L | **215CS**;  **215L^Kc^**;  **211CS** |  |  |  | 192L |  |  |  |  | **203CS^LME^**;  203CS^HME^ |  |  |  |  |  |  | 203CS^R,LME^;  **203CS^R,HME^** |  |  |  |  |  |  |  |  |
|  | Cortisol Levels (morning) |  |  |  |  |  |  |  |  |  |  |  |  |  |  |  |  |  |  |  |  |  |  |  |  |  | **207CSC** |  |  |  |  |  |  |  |
|  | Crying easily or wanting to cry |  |  | **220CS** |  |  | **220CS^I^**;  **220CS** | **220CS^Ch^** |  |  |  |  |  | **220CS** |  |  |  |  |  |  |  |  |  |  |  |  |  |  |  |  |  |  |  |  |
|  | Daily Health Complaints |  |  | **186CS** |  |  | **186CS^D^** |  | **186CS** |  |  |  |  |  |  |  |  |  |  |  |  |  |  |  |  |  |  |  |  |  |  |  |  |  |
|  | Daytime Tiredness |  |  | **186CS** |  |  | **186CS^D^** |  | **186CS** |  |  |  |  |  |  |  |  |  |  |  |  |  |  |  |  |  |  |  |  |  |  |  |  |  |
|  | Depression |  |  | 178L;  179CS^I^;  **186CS** |  | 190CS;  190L | 178L^V^;  **179CS^V^**; **186CS^D^**;  190CS^V^;  190L^V^ | 190CS;  190L | **178L**;  **186CS** |  |  |  | **178L**;  190CS;  **190L^B^**;  **190L^G^** | 179CS |  |  |  |  |  |  |  |  |  |  |  |  |  |  |  |  |  |  |  |  |
|  | Depressive Symptoms |  |  |  |  |  |  | **195L**;  **196STLD^N^** |  | **182CS**;  182L |  | **196STLD^G^;**  196STLD^B^ | **196STLD** |  |  |  |  |  |  |  |  | **199CS** |  |  |  | **209PC^CA^** |  |  |  |  |  |  |  |  |
|  | Emotion Self-Regulation |  |  |  |  |  |  |  |  |  |  |  |  | **202CS** |  |  |  |  |  |  |  |  |  |  |  |  |  |  |  |  |  |  |  |  |
|  | Emotional Problems (SDQ) |  |  | **192L^I^**;  **205PC^WD,G^**;  205PC^WD,B^;  205PC^WE^ |  |  |  |  | 192L | **215CS^Kc^**;  **215L^Bc^**;  **211CS** |  |  | 205PC | 192L |  |  |  |  | 203CS |  |  |  |  |  |  | 203CS^R^ |  |  |  |  |  |  |  |  |
|  | Externalising Problems |  |  | 180CS;  224L |  |  | **214L^V,C,B^**;  214L^V,C,G^;  **224L^B,V^**;  224L^G,V^ |  |  |  |  |  | **180CS**;  214L^B^;  **214L^G^**;  **224L^G†^**;  224L^B^;  224L^G‡^ |  |  |  |  |  |  |  |  |  |  |  |  |  |  |  |  |  |  |  |  |  |
|  | Feeling Lonely |  |  | **220CS** |  |  | **220CS^I^**;  **220CS** | **220CS^Ch^** |  |  |  |  |  | **220CS** |  |  |  |  |  |  |  |  |  |  |  |  |  |  |  |  |  |  |  |  |
|  | Feeling Sad or Blue |  |  | **220CS** |  |  | **220CS^I^**;  **220CS** | **220CS^Ch^** |  |  |  |  |  | **220CS** |  |  |  |  |  |  |  |  |  |  |  |  |  |  |  |  |  |  |  |  |
|  | Feeling that the future is hopeless |  |  | **220CS** |  |  | **220CS^I^**;  **220CS** | **220CS^Ch^** |  |  |  |  |  | **220CS** |  |  |  |  |  |  |  |  |  |  |  |  |  |  |  |  |  |  |  |  |
|  | Having difficulties falling asleep or staying asleep |  |  | **220CS** |  |  | **220CS^I^**;  **220CS** | **220CS^Ch^** |  |  |  |  |  | **220CS** |  |  |  |  |  |  |  |  |  |  |  |  |  |  |  |  |  |  |  |  |
|  | Having little appetite |  |  | **220CS** |  |  | **220CS^I^**;  **220CS** | **220CS^Ch^** |  |  |  |  |  | **220CS** |  |  |  |  |  |  |  |  |  |  |  |  |  |  |  |  |  |  |  |  |
|  | Health Complaints |  |  |  |  |  |  |  |  | **185CS^NA^**;  **185CS^NE^**;  **185CS^SE^**;  185CS^WE^;  **185CS^EE^** |  |  |  |  |  |  |  |  |  |  |  |  |  |  |  |  |  |  |  |  |  |  |  |  |
|  | Hyperactivity/ Inattention (SDQ) |  |  | 192L^I^ |  |  |  |  | 192L | **215CS**;  **215L^Kc^**;  211CS |  |  |  | 192L |  |  |  |  | **203CS^LME^**;  203CS^HME^ |  |  |  |  |  |  | 203CS^R^ |  |  |  |  |  |  |  |  |
|  | Internalising Problems |  |  | 180CS;  224L^B‡^;  224L^†^;  **224L^P,G‡^**;  **224L^St,G‡^** |  |  | **214L^C,V^**;  224L^V^ |  |  | **194L** |  |  | **180CS**;  214L;  224L |  |  |  |  |  |  |  |  |  |  |  |  |  |  |  |  |  |  |  |  |  |
|  | Little interest in doing things |  |  | **220CS** |  |  | **220CS^I^**;  **220CS** | **220CS^Ch^** |  |  |  |  |  | **220CS** |  |  |  |  |  |  |  |  |  |  |  |  |  |  |  |  |  |  |  |  |
|  | Peer Problems (SDQ) |  |  | **192L^I^**;  205PC |  |  |  |  | 192L | **215CS**;  **215L^Kc^**;  **211CS** |  |  | 205PC | 192L |  |  |  |  | **203CS^LME^**;  203CS^HME^ |  |  |  |  |  |  | 203CS^R^ |  |  |  |  |  |  |  |  |
|  | Psychological Distress |  |  |  | **200S** |  |  |  |  |  |  |  |  |  |  |  |  |  |  |  |  |  |  |  |  |  |  |  |  |  |  |  |  |  |
|  | Psychosomatic Symptoms |  |  |  |  |  |  |  |  |  |  |  |  |  |  |  |  | **227CS^P^** |  |  |  |  |  |  |  |  |  |  |  | 227CS^P,B^;  **227CS^P,G^** |  |  |  |  |
|  | Socioemotional Difficulties/ Problems |  |  | 228CS |  |  | **223CS^Con^**;  223CS^C^ | **223CS^Ch^** |  | **223CS**;  **228CS** |  |  | 223CS;  **228CS** |  |  |  |  |  |  |  |  |  |  |  |  |  |  |  |  |  |  |  |  |  |
|  | Stress Recovery |  |  |  |  |  |  |  |  |  |  |  |  |  |  |  |  |  |  |  |  |  |  |  | **187RCE^*^** |  |  |  |  |  |  |  |  |  |
|  | Total Difficulties (SDQ) |  |  | **192L^I^** |  |  |  |  | 192L | **211CS**;  **198CS** |  |  |  | 192L |  |  |  |  | **203CS^LME^**;  203CS^HME^ |  |  |  |  |  |  | 203CS^R^ |  |  |  |  |  |  |  |  |
| **Indicators of Positive Mental Health** | Adaptability | 206PC |  | 206PC^I^ |  |  |  |  |  |  | 206PC |  |  | 206PC |  |  |  |  |  |  |  |  |  |  |  |  |  |  |  |  |  |  |  |  |
|  | Emotional Functioning (HRQoL) |  |  |  |  |  |  |  |  | **216CS** |  |  |  |  |  | **218CSGIS^SA^**^,^;  218CSGIS^RA^ |  |  |  |  |  |  | 218CSGIS | 218CSGIS^SA^;  218CSGIS^RA^ |  |  |  |  |  |  |  |  |  |  |
|  | Emotional Quotient | 206PC |  | 206PC^I^ |  |  |  |  |  |  | **206PC** |  |  | 206PC |  |  |  |  |  |  |  |  |  |  |  |  |  |  |  |  |  |  |  |  |
|  | Emotional Wellbeing (KINDL) |  |  | 205PC |  |  |  |  |  |  |  |  | 205PC |  |  |  |  |  |  |  |  |  |  |  |  |  |  |  |  |  |  |  |  |  |
|  | General Health Status |  |  | 228CS |  |  |  |  |  | 228CS |  |  | 228CS |  |  |  |  |  |  |  |  |  |  |  |  |  |  |  |  |  |  |  |  |  |
|  | Happiness |  | **197STLD^VCh,◊^** |  |  | **197STLD^T,◊^** | 223CS^Con^;  **223CS^C^**;  **197STLD** | **223CS^Ch^**;  **197STLD**;  197STLD^N^ |  | **223CS** |  | **197STLD** | **223CS**;  **197STLD^◊^** |  |  |  |  |  |  |  |  |  |  |  |  |  |  |  |  |  |  |  |  |  |
|  | Health Status |  |  |  |  |  |  |  |  | **185CS^NA^**;  **185CS^NE^**;  185CS^SE^;  **185CS^WE^**;  185CS^EE^ |  |  |  |  |  |  |  |  |  |  |  |  |  |  |  |  |  |  |  |  |  |  |  |  |
|  | Health-related Quality of Life |  |  |  |  |  |  |  |  | **213CS**;  **216CS** |  |  |  |  |  | **218CSGIS^SA^**;  218CSGIS^RA^ |  |  |  |  |  |  | **218CSGIS^SA◦^**;  218CSGIS^SA꙳^;  218CSGIS^RA^ | **218CSGIS^SA^**;  218CSGIS^RA^ |  |  |  |  |  |  |  |  |  |  |
|  | Interpersonal Scores | 206PC |  | 206PC^I^ |  |  |  |  |  |  | 206PC |  |  | 206PC |  |  |  |  |  |  |  |  |  |  |  |  |  |  |  |  |  |  |  |  |
|  | Intrapersonal Scores | 206PC |  | 206PC^I^ |  |  |  |  |  |  | **206PC** |  |  | 206PC |  |  |  |  |  |  |  |  |  |  |  |  |  |  |  |  |  |  |  |  |
|  | Life Satisfaction |  |  |  |  |  |  |  |  | **185CS^NA^**;  **185CS^NE^**;  **185CS^SE^**;  **185CS^WE^**;  185CS^EE^ |  | **197STLD^▫^** |  |  |  |  |  |  |  |  |  |  |  |  |  |  |  |  |  |  |  |  |  |  |
|  | Mental Well-being |  |  |  |  |  |  |  |  |  |  |  |  |  |  |  |  |  |  |  |  | **199CS** |  |  |  |  |  |  |  |  |  |  |  |  |
|  | Positive Affect |  |  |  |  |  |  |  |  |  |  |  |  |  |  |  |  |  |  |  |  |  |  |  |  |  |  |  |  | **217EMAS^PA^** |  |  |  |  |
|  | Positive Emotional Wellbeing |  |  |  |  |  |  |  |  |  |  |  |  |  |  | **226CS^SC^**;  **226CS^MA^**;  226CS^RA^ |  |  |  |  |  |  |  | 226CS |  |  |  |  |  |  | **226CS^SC^**;  226CS^MA^;  226CS^RA^ |  |  |  |
|  | Prosocial Behaviour (SDQ) |  |  |  |  |  |  |  |  | **215CS**;  215L;  **211CS** |  |  |  |  |  |  |  |  | **203CS^LME^**;  203CS^HME^ |  |  |  |  |  |  | 203CS^R,LME^;  **203CS^R,HME^** |  |  |  |  |  |  |  |  |
|  | Psychological Wellbeing |  |  | **192L^I^** |  | **197STLD^T^** | **197STLD** | **197STLD** | **192L** |  |  | **197STLD** |  | 192L |  |  |  |  |  |  |  |  |  |  |  |  |  |  |  |  |  |  |  |  |
|  | Psychosocial Score (HRQoL) |  |  |  |  |  |  |  |  | **216CS** |  |  |  |  |  | **218CSGIS^SA^**;  218CSGIS^RA^ |  |  |  |  |  |  | **218CSGIS^SA◦^**;  218CSGIS^SA꙳^;  218CSGIS^RA^ | 218CSGIS^SA^;  218CSGIS^RA^ |  |  |  |  |  |  |  |  |  |  |
|  | Quality of Life |  |  | **192L^I^** |  |  |  |  | 192L |  |  |  |  | 192L |  |  |  |  |  |  |  |  |  |  |  |  |  |  |  |  |  |  |  |  |
|  | School Functioning (HRQoL) |  |  |  |  |  |  |  |  | **216CS** |  |  |  |  |  | 218CSGIS^SA^;  218CSGIS^RA^ |  |  |  |  |  |  | **218CSGIS^SA◦^**;  218CSGIS^SA꙳^;  218CSGIS^RA^ | 218CSGIS^SA^;  218CSGIS^RA^ |  |  |  |  |  |  |  |  |  |  |
|  | Self-Esteem |  |  | 228CS;  205PC |  |  |  | 197STLD^N^ |  | **228CS** |  | **197STLD^▫^** | **228CS**;  205PC;  197STLD |  |  |  |  |  |  |  |  |  |  |  |  |  |  |  |  |  |  |  |  | **222PP** |
|  | Self-rated Health |  |  | 186CS |  |  | **186CS^D^** |  | **186CS** | **183CS**;  **211CS** |  |  |  |  |  |  |  |  |  |  |  |  |  |  |  |  |  |  |  |  |  |  |  |  |
|  | Self-rated Mental Health |  |  |  |  |  |  |  |  | **183CS** |  |  |  |  |  |  |  |  |  |  |  |  |  |  |  |  |  |  |  |  |  |  |  |  |
|  | Social Functioning (HRQoL) |  |  |  |  |  |  |  |  | **216CS** |  |  |  |  |  | **218CSGIS^SA^**;  218CSGIS^RA^ |  |  |  |  |  |  | **218CSGIS^SA◦^**;  218CSGIS^SA꙳^;  **218CSGIS^RA^** | **218CSGIS^SA^**;  218CSGIS^RA^ |  |  |  |  |  |  |  |  |  |  |
|  | Social Functioning (KINDL) |  |  | 205PC |  |  |  |  |  |  |  |  | 205PC |  |  |  |  |  |  |  |  |  |  |  |  |  |  |  |  |  |  |  |  |  |
|  | Stress Management Score | 206PC |  | **206PC^I^** |  |  |  |  |  |  | **206PC** |  |  | 206PC |  |  |  |  |  |  |  |  |  |  |  |  |  |  |  |  |  |  |  |  |
|  | Wellbeing |  |  |  |  |  |  |  |  |  |  |  |  |  |  |  |  |  |  |  |  |  |  | **210L^QN^**;  **210L^QL^** |  |  |  |  |  |  |  |  |  |  |
| **Cognitive Functioning** | Applied Problems Score |  |  | 224L^†^;  **224L^Ch,B‡^**;  **224L^P,G‡^**;  **224L^St,G‡^**;  225L |  |  | 224L^V,B^;  224L^V,G‡^;  **224L^V,G†^**;  225L^V^;  225L^C,G†,‖^;  **225L^C,G‡^**;  **225L^C,B†^** |  |  |  |  |  | 224L;  225L |  |  |  |  |  |  |  |  |  |  |  |  |  |  |  |  |  |  |  |  |  |
|  | Attention (hyperactivity/ inattention) |  |  |  |  |  |  |  |  |  |  |  |  |  |  |  |  |  |  |  |  |  |  |  |  |  |  |  | **208L** | **208L** |  |  |  |  |
|  | Attention Restoration |  |  |  |  |  |  |  |  |  |  |  |  |  |  |  |  |  |  |  |  |  |  |  | **187RCE^*^** |  |  |  |  |  |  |  |  |  |
|  | Cognitive Performance/ Functioning |  |  |  |  |  | 193CS^V^;  201L |  |  |  |  |  | 201L;  193CS |  |  |  |  |  |  |  |  |  |  |  |  |  |  |  |  |  |  |  |  |  |
|  | Digit Span Scores |  |  |  |  |  |  |  |  |  |  |  |  |  |  |  |  |  |  |  |  |  |  |  |  |  |  |  | 208L | **208L** |  |  |  |  |
|  | Directed Attention Capacity |  |  |  |  |  |  |  |  |  |  |  |  |  |  |  |  |  |  |  |  |  |  |  |  |  |  | **219L** |  |  |  |  |  |  |
|  | Letter-Word Score |  |  | 224L;  **225L^E,B‡^**;  225L |  |  | 224L^V,B^;  224L^V,G†^;  **224L^V,G‡^**;  225L^V,C^ |  |  |  |  |  | 224L^G^;  224L^B†^;  **224L^B‡^**;  225L |  |  |  |  |  |  |  |  |  |  |  |  |  |  |  |  |  |  |  |  |  |
|  | Passage Comprehension Score |  |  | 224L^G†^;  **224L^Ch,B‡^**;  **224L^Ch,B†^**;  **224L^St,B†^**;  **224L^P,G‡^**;  225L |  |  | 224L^V,B^;  224L^V,G‡^;  **224L^V,G†^**;  **225L^V,†^**;  225L^V,‡,‖^;  225L^C,B^;  225L^C,G†‖^;  **225L^C,G‡^** |  |  |  |  |  | 224L^B^;  224L^G‡^;  **224L^G†^**;  225L |  |  |  |  |  |  |  |  |  |  |  |  |  |  |  |  |  |  |  |  |  |
| **Academic Achievement** | Academic/ School Achievement/ Performance | 191CS |  | 180CS;  186CS;  **191CS**;  **191CS^Ch^**;  **191CS^St^**; |  |  | **186CS^D^**;  **191CS^C^**;  181L^V^ |  | **186CS**;  **191CS^Ch^**;  **191CS^P^** | **191CS** | **191CS** |  | 180CS;  181L | **191CS** |  |  | 188CS^Nat^ |  |  |  |  |  |  |  |  |  |  |  |  |  |  |  |  |  |
|  | English Grades/ Achievement |  | 184L |  |  | 184L | **177L^V^**;  184L | **184L^❖^** | **177L** | 184L |  | **177L** | 177L;  **184L** |  |  |  |  |  |  |  |  |  | 229CS^S^ |  |  |  |  |  |  |  |  | **229CS^S^** |  |  |
|  | German Grades |  |  |  |  |  |  |  |  |  |  |  | 177L |  | 212CSGIS^H,S,HS^ |  |  |  |  |  | 212CSGIS^H^;  **212CSGIS^S^**;  **212CSGIS^HS^** |  |  |  |  | 212CSGIS^H,S,HS^ |  |  |  |  |  | 212CSGIS^H,S,HS^ |  |  |
|  | Graduation Rates |  |  |  |  |  |  |  |  |  |  |  |  |  |  |  | 188CS^WIN^ |  |  |  |  |  |  |  | **188CS^*^** |  |  |  |  |  |  |  | 188CS |  |
|  | Maths Grades/ Achievement/ Score |  | **184L** |  |  | 184L | 181L;  177L^V^;  184L | 184L;  **204L** | **177L** | 184L |  | 177L | **181L^•^**;  **204L**;  **184L** |  | 212CSGIS^H,S,HS^ |  |  |  |  | 221QE | 212CSGIS^H,S,HS^ |  | 229CS^S^ |  |  | 212CSGIS^H,S,HS^ |  |  |  |  |  | **229CS^S^**;  212CSGIS^HS,S^;  **212CSGIS^H,Mun^** |  |  |
|  | Maths Growth |  |  |  |  |  |  | 204L |  |  |  |  | **204L** |  |  |  |  |  |  |  |  |  |  |  |  |  |  |  |  |  |  |  |  |  |
|  | Michigan Merit Award Recipients |  |  |  |  |  |  |  |  |  |  |  |  |  |  |  | 188CS^WIN^ |  |  |  |  |  |  |  | **188CS^*^** |  |  |  |  |  |  |  | **188CS^¤^** |  |
|  | Reading Growth |  |  |  |  |  |  | 204L |  |  |  |  | 204L |  |  |  |  |  |  |  |  |  |  |  |  |  |  |  |  |  |  |  |  |  |
|  | Reading Performance/ Score |  |  |  |  |  |  | 204L |  |  |  |  | 204L |  |  |  |  |  |  |  |  |  |  |  |  |  |  |  |  |  |  |  |  |  |
|  | Science Grades |  |  |  |  |  | 177L^V^ |  | 177L |  |  | **177L** | 177L |  |  |  |  |  |  |  |  |  |  |  |  |  |  |  |  |  |  |  |  |  |
|  | Students planning to go to college |  |  |  |  |  |  |  |  |  |  |  |  |  |  |  | **188CS^WIN^** |  |  |  |  |  |  |  | **188CS^*^** |  |  |  |  |  |  |  | **188CS** |  |
| **Other** | Connectedness to Nature |  |  |  |  |  |  |  |  |  |  |  |  |  |  |  |  |  |  |  |  |  |  |  |  |  |  |  |  |  |  |  |  | **222PP** |

***Notes.*** Study reference number and study design in brackets. Studies reporting an **unfavourable** association between the exposure and outcome are bolded. Studies reporting a **favourable** association between the exposure and outcome are bolded and underscored. Studies reporting no statistically significant association are not bolded.

*Study Designs*: CS = cross-sectional; CSGIS = cross-sectional geographic information systems study; EMAS = ecological momentary assessment study; L = longitudinal; PC = prospective cohort; PP = pretest-posttest design; QE = quasi-experiment; RCE = randomised controlled experiment; S = surveillance; STLD = surveillance time lag design.

*When results differ for subgroups*: B = boys; EE = Eastern Europe; G = girls; HME = children with mothers who have high education level; LME = children with mothers who have low education level; MA = in metropolitan areas; Mun = in Munich; NA = North America; NE = Northern Europe; Alc = in the presence of parental alcoholism; RA = in rural areas; SA= suburban/urban areas; SC = in small cities; SE = Southern Europe; WE = Western Europe; ‡ = White children; † = Black children; • = for those in the second SES quartile only; ◊ = association significant for 8^th^ & 10^th^ graders only; Kc = association was only significant for the ‘K Cohort’; Bc = association was only significant for the ‘B Cohort’; ▫ = for 12^th^ graders only; ‖ = Latino/a children.

*Green Time Exposure details:* CA = in childhood/adolescence; H = home; HS = home and school combined; Nat = nature; P = play; PA = physical activity; QL = quality; QN = quantity; R = residential; S = school; WIN = window area; * = green views; ¤ = when considering lawn landscaped areas; ◦ = grass/shrubs; ꙳ = dense vegetation.

*Screen Time Exposure details:* C = computer; Ch = chatting/communication; Con = game console; D = digital; E = for email use; I = and internet; IMing = instant messaging; N = to read/access the news; P = play; St = studying; T = texting; V = video; Vch = video chatting; W = for website use; WD = weekday ST; WE = weekend ST; ❖ = association was significant for 1.5 – 3 hours/day only.

*Psychological Outcomes:* SDQ = Strengths & Difficulties Questionnaire; HRQOL = Health-related quality of life.

*Study Notes*: Study 189L is not included in table as it compared cluster types; Studies 224L & 225L report many different associations (e.g., by screen activity, gender, and ethnicity); the majority (especially those highlighted as important by study authors) are presented.
